# The repertoire of olfactory C family G protein-coupled receptors in zebrafish: candidate chemosensory receptors for amino acids

**DOI:** 10.1186/1471-2164-7-309

**Published:** 2006-12-08

**Authors:** Tyler S Alioto, John Ngai

**Affiliations:** 1 Department of Molecular and Cell Biology, Functional Genomics Laboratory, and Helen Wills Neuroscience Institute, University of California, Berkeley, California 94720, USA; 2 Center for Genomic Regulation, Doctor Aiguader, 88, E-08003, Barcelona, Spain

## Abstract

**Background:**

Vertebrate odorant receptors comprise at least three types of G protein-coupled receptors (GPCRs): the OR, V1R, and V2R/V2R-like receptors, the latter group belonging to the C family of GPCRs. These receptor families are thought to receive chemosensory information from a wide spectrum of odorant and pheromonal cues that influence critical animal behaviors such as feeding, reproduction and other social interactions.

**Results:**

Using genome database mining and other informatics approaches, we identified and characterized the repertoire of 54 intact "V2R-like" olfactory C family GPCRs in the zebrafish. Phylogenetic analysis – which also included a set of 34 C family GPCRs from fugu – places the fish olfactory receptors in three major groups, which are related to but clearly distinct from other C family GPCRs, including the calcium sensing receptor, metabotropic glutamate receptors, GABA-B receptor, T1R taste receptors, and the major group of V2R vomeronasal receptor families. Interestingly, an analysis of sequence conservation and selective pressure in the zebrafish receptors revealed the retention of a conserved sequence motif previously shown to be required for ligand binding in other amino acid receptors.

**Conclusion:**

Based on our findings, we propose that the repertoire of zebrafish olfactory C family GPCRs has evolved to allow the detection and discrimination of a spectrum of amino acid and/or amino acid-based compounds, which are potent olfactory cues in fish. Furthermore, as the major groups of fish receptors and mammalian V2R receptors appear to have diverged significantly from a common ancestral gene(s), these receptors likely mediate chemosensation of different classes of chemical structures by their respective organisms.

## Background

The vertebrate olfactory system receives and decodes sensory information from a myriad chemical cues. The first step in this process is the recognition of these cues by receptors expressed by the primary sensory neurons in the olfactory epithelium (reviewed in refs. [1,2]). Receptor-mediated activity within the population of olfactory sensory neurons is then interpreted by the brain to identify the molecular nature of the odorant stimulus. A large multigene family thought to encode odorant receptors was initially identified in the rat [3] and belong to what is now referred to as the "OR" superfamily of odorant receptors (reviewed in [4]). The predicted structure of these receptors exhibits a seven transmembrane domain topology characteristic of the "A family" or rhodopsin class of G protein-coupled receptors (GPCRs). The size of the OR gene family in mammals is extremely large and is estimated to contain over 1000 individual genes in some species [5-9]. In the fish, the size of the OR repertoire appears to be much smaller and appears to contain only ~40 to ~140 genes, depending on the species examined [10,11]. More recently, members of the trace amine-associated receptor (TAAR) family were shown to be expressed in mouse olfactory neurons and are thought to mediate the reception of amine-based chemosensory cues [12].

Two types of GPCRs unrelated to the OR or TAAR families are expressed in the mammalian vomeronasal organ: the V1R receptors [13,14] and the V2R receptors [15-18]. The V1R receptors are expressed within the subpopulation of G_αi_-expressing VNO sensory neurons [14]. Genome-wide surveys have revealed the presence of approximately 100 V1R genes in the mouse genome [6,13]. The V2R receptors belong to the "C family" of GPCRs, which includes the metabotropic glutamate receptors (mGluR), extracellular calcium sensing receptors (CaSR), and GABA-B receptors [19]. Members of this receptor family are characterized by their long N-terminal extracellular domain, which contains the primary determinants for ligand binding [20,21]). The mouse and rat genomes each encode approximately 60 V2R genes [18]; these receptors are expressed in the subclass of G_αo_-expressing neurons in a pattern complementary to V1R/G_αi _expression [15-17]. Because of their expression in the vomeronasal organ – a structure specialized for the detection of non-volatile cues, including pheromones – the V1R and V2R receptors have been widely postulated to represent pheromone receptors (reviewed by [4]). Indeed, a number of studies have demonstrated that specific V1R- and V2R-expressing vomeronasal neurons respond to known pheromones [22-24]; however, formal proof that the V1R and V2R are pheromone receptors awaits a direct demonstration of ligand-receptor interactions between such compounds and these receptors.

In the fish, receptors belonging to the C family of GPCRs have been shown to be expressed in the olfactory epithelium [25-27]. The olfactory C family GPCRs are expressed by the subpopulation of microvillous sensory neurons in the fish's single olfactory organ, distinct from the ciliated sensory neurons which express members of the OR family of odorant receptors [25,27]. Significantly, two orthologous receptors from the goldfish and zebrafish (called receptor 5.24 and receptor ZO6, respectively) are activated by amino acids [27,28], which are potent feeding cues in fish [29-31]. These observations raise the possibility that the olfactory C family GPCRs as a group represent a family of amino acid-sensing receptors in teleost fish.

To gain insights into the evolution and function of olfactory C family GPCRs, we performed an analysis of this receptor gene family in the zebrafish, *Danio rerio*. Through genome database mining of the zebrafish genome sequence provided by the Sanger Institute *Danio rerio *Sequencing Project, we identified and characterized the complete repertoire of olfactory C family GPCRs. Our analysis identified 62 genes (54 encoding intact, full-length receptors), which can be divided into 21 subfamilies. Although two of the intact zebrafish receptor genes appear to be orthologous to mammalian V2R or V2R-like genes, the major group of zebrafish receptors form a clade distinct from the mammalian V2R receptors. In addition, our analysis of the zebrafish receptors revealed – in all family members – a conserved signature motif previously shown to be involved in ligand binding in other amino acid-sensing receptors [32,33]. In contrast, other amino acid positions predicted to form contact sites in the ligand binding pocket show marked divergence within the zebrafish receptor family. Together these observations suggest that the zebrafish olfactory C family GPCRs comprise a family of receptors that has evolved to recognize a diverse array of amino acid and/or amino acid-based ligands.

## Results and discussion

### Prediction of zebrafish OlfC genes

The second (Zv2) and third (Zv3) draft zebrafish genome assemblies [34] of whole genome shotgun sequence (5.7× coverage) were searched for *OlfC *gene sequences using homology to known fish OlfC proteins as a guide. A final search of the sixth draft assembly (Zv6) with the identified *OlfC *genes revealed no additional sequences. The resulting genes were then mapped to the Zv6 assembly.

In contrast to the typically uninterrupted coding sequence of *OR *genes, the intron-exon structure of the genes encoding the C family GPCRs comprises a minimum of 6 exons (e.g., see ref. [35]). Our gene prediction strategy was to combine a low-threshold BLAST search with profile Hidden Markov Model-(HMM) based gene prediction with the program Genewise [36]. This process was repeated in an iterative fashion, as follows. The zebrafish genome assembly was subjected to TBLASTN search using known full-length olfactory C family GPCRs from zebrafish, goldfish and fugu. Initial query sequences included goldfish olfactory receptors 5.24, 5.3, GFB1, and GFB8, zebrafish ZO6, and fugu Ca02.1, Ca09, Ca12, Ca13 and Ca15.1 [25-27]. The gene prediction program Genewise was then run on the genomic sequences surrounding each unique BLAST hit using a profile hidden Markov model (HMM) of the gene family (see Methods for details). The Genewise predictions were examined manually and extended to appropriate start and stop codons based on alignment of the amino acid translation to known or previously predicted olfactory C family GPCR sequences. Splice site predictions were also edited when possible to minimize the occurrence of gaps at splice junctions in the alignment and maintain intron positions with respect to the amino acid sequence alignment. At this stage, predicted genes highly similar to the CaSR, mGluRs and T1Rs were set apart from the putative olfactory sequences. In each round, the newly-predicted genes were added as queries for the next BLAST search, and a new profile HMM was constructed for use in the next round of gene prediction. The family of putative olfactory C family GPCRs is designated "*OlfC*" for Olfactory C family GPCR (see below for discussion regarding nomenclature).

*OlfC *gene sequences were considered intact if they 1) begin with a signal sequence, 2) end in a stop codon after the seventh predicted transmembrane domain, 3) show significant alignment to all six stereotypical OlfC exons, and 4) possess no internal frame shifts, significant deletions (excluding those caused by gaps in the genome assembly) or stop codons. Predicted genes were categorized as full-length pseudogenes if they at met the first three criteria but failed the fourth. Sequences were considered partial genes if they failed one of the first three criteria but met the fourth. Finally, predicted genes were considered partial pseudogenes if they failed all four criteria.

Using these criteria, our search identified 54 intact *OlfC *genes, 2 partial genes, 3 full-length pseudogenes and 1 partial pseudogene, for a total of 60 sequences. Two additional partial pseudogenes (too small to meet our length threshold) were recently reported by Hashiguchi and Nishida [37]; see below). We therefore included them in the total count of 62 *OlfC *genes (Table 1; [see Additional files 1, 2, 3, 4]) but excluded them from further analysis. All full-length genes exhibit a conserved gene structure, with the protein coding sequence encoded by 6 exons [see Additional file 5]. Variation in exon length is evident, even within individual subfamilies, although variation in exon length is greater between subfamilies [see Additional file 3]. Interestingly, however, the phases of intron/exon boundaries within codons are strictly conserved across all members of the family [see Additional file 3].

**Table 1:** Summary of zebrafish *OlfC *genes.

**Intact genes^a^**	54
**Partial genes^b^**	2
**Full-length pseudogenes^c^**	3
**Partial pseudogenes^d, e^**	3
**Total^e^**	**62**

^a ^Intact genes are defined as those sequences that 1) contain a leading signal sequence, 2) end in a stop codon after the seventh predicted transmembrane domain, 3) show significant alignment to all six stereotypical OlfC exons, and 4) possess no internal frame shifts, significant deletions (excluding those caused by gaps in the genome assembly) or stop codons.

^b ^Partial genes comprise those sequences that fail one of the first three criteria (above) but meet the fourth.

^c ^Sequences are categorized as full-length pseudogenes if they meet the first three criteria but fail the fourth.

^d ^Sequences that fail to meet all four criteria are categorized as partial pseudogenes.

^e ^Includes two partial pseudogenes identified in [37] but not identified in this study.

A recent study based on the 4^th ^assembly of the draft zebrafish genome (Zv4) identified a total of 89 OlfC sequences, including 38 partial sequences [37]. Forty of the 89 sequences appear to have frame shifts or stop codons. We ascribe the discrepancies with our results in part to the difference in genome assemblies used for the two analyses (Zv4 alone vs. the Zv2, Zv3 and Zv6 assemblies used here). In the present analysis, we found that many of the non-overlapping sequences previously identified as distinct genes [37] in fact map to a smaller set of common, intact genes (note the clustering of multiple, previous gene designations to single identified genes in [see Additional file 2]). We also found that all full-length *OlfC *gene sequences have a conserved gene structure [see Additional file 3]; previously reported variations to this organization [37] are likely due to sequencing errors in earlier genome assemblies and inaccuracies in gene predictions. Overall, our analysis identified all but two pseudogenes found in [37] plus an additional three intact genes, one partial gene and one full-length pseudogene. Based on these observations and the higher quality of our gene models (as evidenced by the greater proportion of complete gene sequences in the present analysis), we believe that our study provides a more accurate representation of the zebrafish *OlfC *gene family.

In addition to the 62 OlfC sequences described above, our analysis also identified other C family GPCRs in the zebrafish genome: one putative calcium sensing receptor and 4 T1R-like putative taste receptors. Our search for these other receptor sequences was not exhaustive, however; it is therefore possible that additional paralogs of these receptors escaped identification in our analysis. Nonetheless, it is interesting to note that the four T1R-like receptors are highly similar to the three previously-identified classes of T1R taste receptors in mammals [38-41]: T1R1 (zebrafish T1Ra), T1R2 (zebrafish T1Rb1 and T1Rb2), and T1R3 (zebrafish T1Rc) (see below).

The identity of the *OlfC *gene family as a bona fide olfactory receptor family was confirmed by RNA in situ hybridizations. Probes for 46 intact *OlfC *genes were hybridized individually to zebrafish olfactory epithelium (T. Alioto, P. Luu, E. VanName, J. Fan, J. Ngai, unpublished results; [see Additional file 2]). Of this group, 42 gave detectable signal in olfactory sensory neurons. Forty probes localized to cells in a punctate pattern consistent with the expression of one or a few receptor genes per neuron. Two receptors, OlfCa1 and OlfCc1, exhibited broad expression in a large fraction of neurons. These receptors are orthologous to goldfish receptors 5.24 and 5.3, respectively, which were previously shown to be expressed widely in the goldfish olfactory epithelium [27]. OlfCc1 and goldfish receptor 5.3 are orthologous to the mammalian V2R2 vomeronasal receptor, which is expressed in a large fraction of vomeronasal neurons and co-expressed with other "punctate" V2R receptors [16]. Together our results suggest that OlfCa1 and OlfCc1 – which comprise two clades distinct from the main group of zebrafish OlfC receptors (see below) – are the two broadly expressed receptors of the repertoire and are co-expressed with a single "punctate" receptor in each sensory neuron.

### OlfC nomenclature and classification

Olfactory receptors belonging to the "OR" superfamily have been classified into monophyletic groups, with family members sharing greater than 40% amino acid identity and subfamily members sharing greater than 60% amino acid identity [42]. In the case of the zebrafish *OlfC *genes, we found that a subfamily threshold of 65% amino acid identity worked best for the generation of monophyletic clades, which we believe correspond to groups of recently duplicated *OlfC *genes. Using this threshold as an operational guideline, we classified the zebrafish *OlfC *genes into 21 subfamilies, with 17 containing full-length *OlfC *gene sequences [see Additional file 2] (see below for phylogenetic reconstructions). The average percent identity between subfamilies is approximately 46%, with the maximum observed percent identity between any two OlfCs of different subfamilies being 61%. The average percent identity among members within a subfamily is ~81%, with the minimum identity between any two members of any subfamily being 72%.

To unify the naming for zebrafish *OlfC *genes, we propose a revised nomenclature based on the following rationale. First, the prefix "OlfC" was adopted as the designation for the Olfactory C family GPCRs identified in this and previous studies. In addition to reflecting the olfactory-specific nature of these receptors, this designation is consistent with our phylogenetic analysis (see below), which indicates that the OlfC receptors form a family distinct from other C family GPCRs, including the mammalian V2R receptors. Both newly-predicted and previously-described zebrafish *OlfC *genes were named (or re-named) according to subfamily membership. Subfamilies were designated by letters starting with "a" and ending with "y" (skipping "i," "l," "o," and "p"). Within subfamilies, *OlfC*s were numbered sequentially according to genomic position, if known. The new nomenclature showing subfamily membership and correspondence to previously-identified zebrafish *OlfC *genes is shown in Supplementary Materials [see Additional file 2].

### Genomic distribution of zebrafish OlfC genes

**Figure 1:**
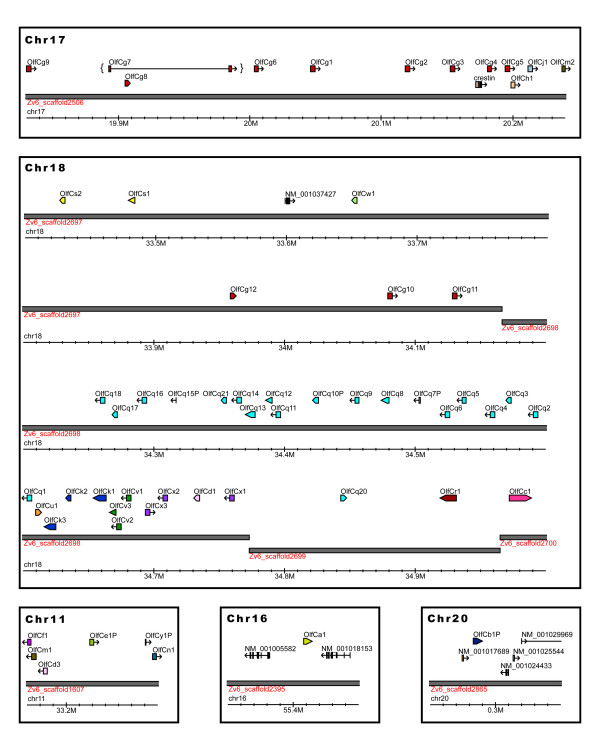
***OlfC *genes are clustered in the zebrafish genome**. Zebrafish *OlfC *genes were mapped to the Zv6 draft genome assembly. Shown are the positions of predicted *OlfC *genes in three clusters on chromosomes 11, 17 and 18 as well as the two singlets on chromosomes 16 and 20. Subfamily members are in most cases found in contiguous clusters and often share the same transcriptional orientation. The interrupted coding sequence of OlfCg7 (in brackets) is likely due to the unfinished state of scaffold 2506 in the Zv6 genome assembly. Also shown are non-OlfC RefSeq sequences and zebrafish mRNAs from the UCSC genome browser [78].

Previous studies have demonstrated that *V2R *and *OlfC *genes are clustered in the rodent and fugu genomes, respectively [18,26]. In the present study, we found that 59 of the 62 identified zebrafish *OlfC *genes map to known chromosomal locations (Figure 1; [see Additional file 2]). Fifty seven genes reside in three clusters, with 39 genes on chromosome 18, 12 genes on chromosome 17, and 6 genes on chromosome 11; one gene each localizes to chromosome 16 and chromosome 20. From our analysis of the Zv6 genome assembly, there is little evidence for large segmental duplications of receptor-containing loci; we find only a few instances of apparent interchromosomal duplications resulting in subfamily members residing on two chromosomes (subfamily g on chromosomes 17 and 18, subfamily d on chromosomes 11 and 18, and subfamily m on chromosomes 11 and 17). In most cases, genes within a given subfamily are found in the same chromosomal cluster and share a common transcriptional orientation. Together these observations suggest tandem duplication(s) as the primary mechanism for expansion of the *OlfC *gene repertoire.

A previous analysis placed the zebrafish *OlfC *genes on ten chromosomes, with two major clusters of 34 genes on chromosome 18, 10 genes on chromosome 17, and none on chromosome 11 [37]. However, in the present analysis using the more recent Zv6 genome assembly, we found that the *OlfC *genes with identifiable chromosomal locations map only to chromosomes 11, 16, 17, 18 and 20; many of the genes localized by Hashiguchi and Nishida [37] to the seven other chromosomes were partial sequences that ultimately map to the major clusters on chromosomes 11, 17 and 18 in the updated Zv6 genome assembly. In addition, Hashiguchi and Nishida identified two possible instances of recent tandem duplications [37]: one within the cluster on chromosome 18 resulting in a near-perfect 64 kb duplication with 3 *OlfC *genes, and another between chromosomes 17 and 20, yielding an apparent 60 kb duplication with 4 genes. However, our analysis of these regions using the Zv2, Zv3, Zv5 and Zv6 assemblies indicates that these apparent duplications represent artifacts caused by errors in the Zv4 genome assembly. With the exception of 2 pseudogenes, all of the 89 OlfC sequences identified previously [37] were detected with our gene mining pipeline and all of them (including the 2 pseudogenes) are accounted for in our analysis. Largely consistent with our gene predictions and chromosomal mapping, a more recent report by Hashiguchi and Nishida [43] based on the Zv5 assembly identified 57 zebrafish OlfC sequences (46 encoding potentially functional genes) that were localized to chromosomes 5, 17 and 18. We attribute the minor discrepancies between [43] and our study to differences in the Zv5 and Zv6 zebrafish genome assemblies.

### Phylogeny of zebrafish OlfC genes

**Figure 2:**
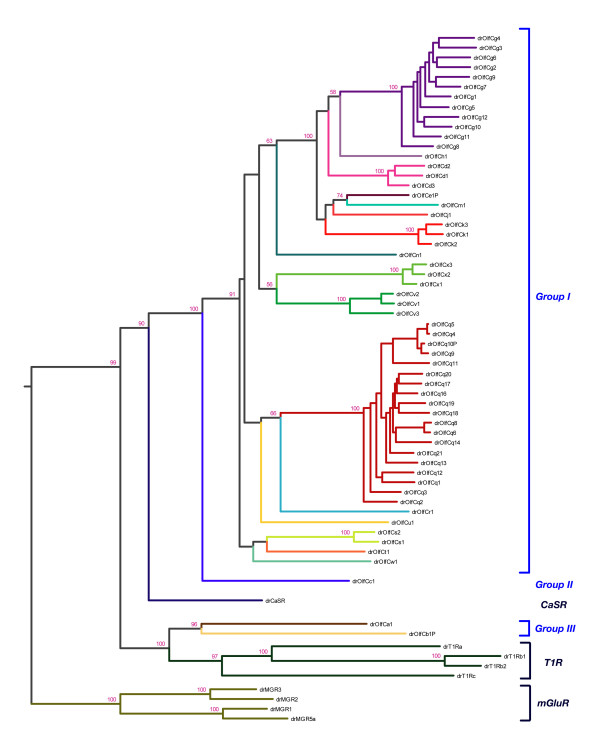
**Phylogeny of the zebrafish OlfC family**. A phylogenetic tree of 57 full-length OlfC sequences (54 genes and 3 pseudogenes) plus other C family GPCRs was constructed using a maximum likelihood (ML) algorithm (see Methods). Bootstrap support is indicated at each node. The full-length *OlfC *genes were placed into 17 subfamilies, forming clades with 100% bootstrap support. Average percent identity between subfamilies is 46%. *CaSR*: putative calcium sensing receptor; *mGluR*: metabotropic glutamate receptors; *T1R*: T1R-like putative taste receptors.

We performed a phylogenetic analysis of the zebrafish *OlfC *genes in order to determine the relationships between members of this gene family. To this end, phylogenetic trees of the 57 full-length OlfC sequences (54 intact genes and 3 full-length pseudogenes) and other C family GPCRs were constructed using maximum likelihood or neighbor joining algorithms (see Methods). Figure 2 shows the results of a phylogenetic reconstruction using maximum likelihood analysis, which classifies the full-length zebrafish OlfC genes into monophyletic clades representing 17 subfamilies showing on average 46% inter-subfamily identity. Assignment of genes at the subfamily level is identical using either maximum likelihood or neighbor joining methods, although some differences are manifested at deeper branches (data not shown). The subfamilies in turn cluster into 3 larger groups: Group I, the largest group, consisting of 52 intact genes and 2 full-length pseudogenes; Group II, consisting of a single intact gene; and Group III, containing one intact gene plus a full-length pseudogene. All OlfC groups form clades that are clearly distinct from the CaSR, mGluR, and T1R-like taste receptor families. Group I and Group II genes reside on a common branch of the phylogenetic tree; their proximity to the calcium sensing receptor (CaSR) suggests a previous duplication and expansion from a most recent common ancestral gene (MRCA) shared by the present-day CaSR.

Interestingly, Group III – which contains OlfCa1, an amino acid receptor activated by glutamate [28] – is divergent from Group I and Group II. Reconstructions using either maximum likelihood (Figure 2) or neighbor joining (data not shown) place Group III closer to the putative taste receptors than to the Group I and Group II OlfC receptors, suggesting an MRCA for the T1R and Group III receptors not shared with the Group I or Group II receptors. However, based on amino acid sequence similarity alone, Group III is roughly equidistant from Group I/Group II (average 31% identity) and the family of T1R-like putative taste receptors (average 29% identity). While this apparent discrepancy could be explained by the longer branch lengths of the T1R family, suggestive of accelerated functional/sequence divergence, conclusions about the origins of the Group III OlfC receptors should be tempered by these latter observations.

### Comparison of fish OlfC and mammalian V2R receptors

**Figure 3:**
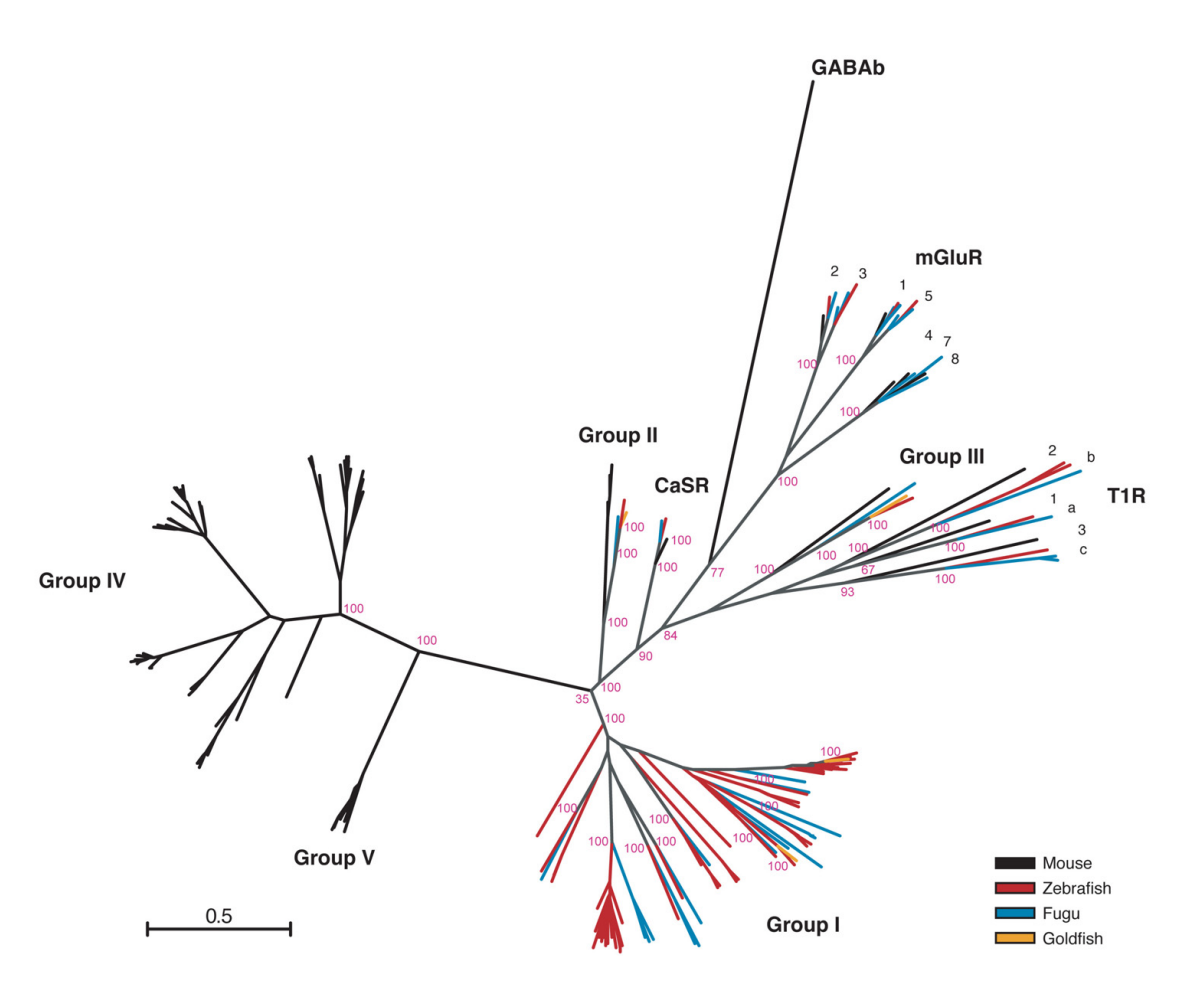
**Phylogeny of C family GPCRs from zebrafish, fugu, goldfish and mouse**. A phylogenetic tree of selected zebrafish, fugu, goldfish and mouse C family receptors was constructed using a maximum likelihood algorithm and displayed in radial format. Groups I – V all form clades distinct from the CaSR, T1R, mGluR and GABA-B families. Most of the fish OlfC receptors form a clade (Group I) distinct from the mouse V2R receptors (mmV2R) found in Groups II, IV and V. Group II contains both fish OlfC receptors (zebrafish: drOlfCc1; goldfish: ca5.3; fugu: 735220) and mouse V2R2-like (mmV2R2) sequences. Group III contains receptors from zebrafish (drOlfCa1), goldfish (ca5.24), fugu (179742) and mouse (mmGprc6a). Scale bar indicates the number of amino acid substitution per site.

To gain additional insight into the evolution of the OlfC receptor repertoire, we compared the zebrafish OlfC sequences to mammalian V2R receptors, OlfC receptors identified in two other teleost fish species (goldfish and fugu), and other C family GPCRs. Phylogenetic trees were constructed using intact genes from zebrafish (63 genes), mouse (72 genes), goldfish (4 genes), and fugu (34 genes) (see Methods for the list of genes included in this analysis). A phylogenetic tree generated using maximum likelihood is shown in Figure 3. This analysis reveals that the major group of fish *OlfC *genes (Group I, containing genes from zebrafish, goldfish and fugu) is distinct from the largest set of mammalian V2R sequences, which comprise two related clades designated here as Group IV and Group V (also referred to as "Family A" and Family B," respectively, in ref. [18]). Thus, the fish Group I OlfC receptors and mammalian Group IV/V V2R receptors appear to have diverged significantly from a common ancestral gene(s).

Group II contains genes from zebrafish (OlfCc1), goldfish (receptor 5.3), fugu (735220), and mouse (three genes closely related to and including V2R2). In addition to their sequence similarity, members of this group are expressed broadly in their respective organisms' sensory epithelium [27,44], reflecting a conserved mode of gene regulation. Group III appears to contain a single receptor in the zebrafish (OlfCa1), goldfish (receptor 5.24 [27]), fugu (179742), or mouse (gprc6a [45]); mammalian gprc6a shows about 40% amino acid similarity to the corresponding fish receptors in this group [45,46]. The zebrafish, goldfish and mouse receptors in Group III have all been shown to be activated by amino acid ligands [27,28,47,48]. However, while the zebrafish and goldfish genes are expressed in the olfactory epithelium, mammalian gprc6a is expressed in a number of non-olfactory tissues, but not in the olfactory system [45,46]. Thus, while these receptors appear to be orthologous, their regulation in tissue-specific gene expression has diverged significantly between fish and mammals.

The 19 identified fugu OlfC receptors [see Additional file 6] can be placed into 10 of the 21 subfamilies defined above for the zebrafish (subfamilies a, c, h, j, k, q, r, t, u and v). Four fugu receptors (744432, 611613, 594197, and 744222) cannot be classified into these subfamilies and thus form four fugu-specific subfamilies. We conclude from this comparison that the OlfC family had already diverged into the present-day subfamilies in the most recent common ancestor of the cyprinid (zebrafish and goldfish) and pufferfish lineages, prior to the cyprinid-pufferfish split (see also [43]). Subsequent differential gene expansion (mainly evident in the zebrafish branch) and gene loss (more prevalent in the fugu branch) probably occurred following speciation of these two teleost lineages.

### Patterns of sequence conservation and divergence implicate the OlfC receptors as a family of amino acid-binding proteins

**Figure 4:**
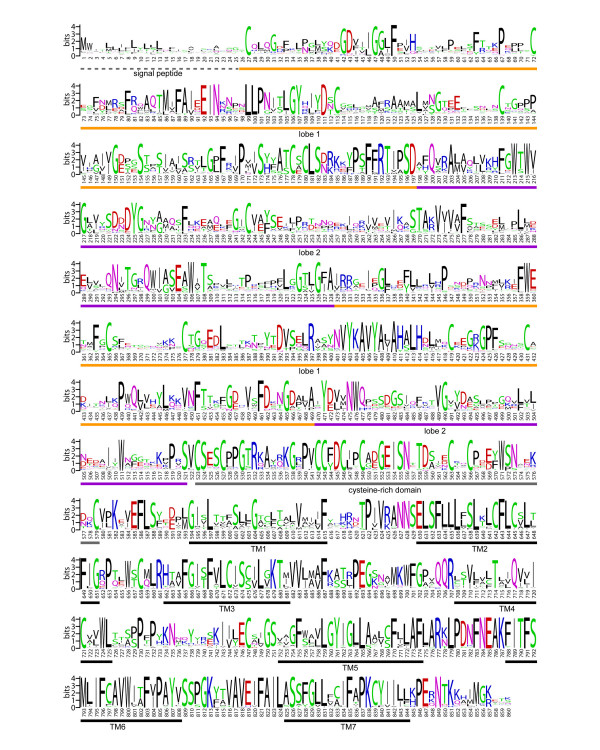
**Sequence logo of the zebrafish OlfC family**. Conservation of predicted amino acid sequence for the zebrafish OlfC repertoire is displayed as a sequence logo. In this representation, the relative frequency with which an amino acid appears at a given position is reflected by the height of its one-letter amino acid code in the logo, with the total height at a given position proportional to the level of sequence conservation. For this analysis, the subset of 54 full-length intact zebrafish OlfC amino acid sequences was extracted from the alignment of all 62 OlfCs and gaps were removed with respect to OlfCq6. The regions corresponding to the signal peptide, lobe 1, lobe 2, the cysteine-rich domain and the transmembrane (TM) domains are indicated. *Y axis*, information content. *X axis*, residue position with respect to OlfCq6.

**Figure 5:**
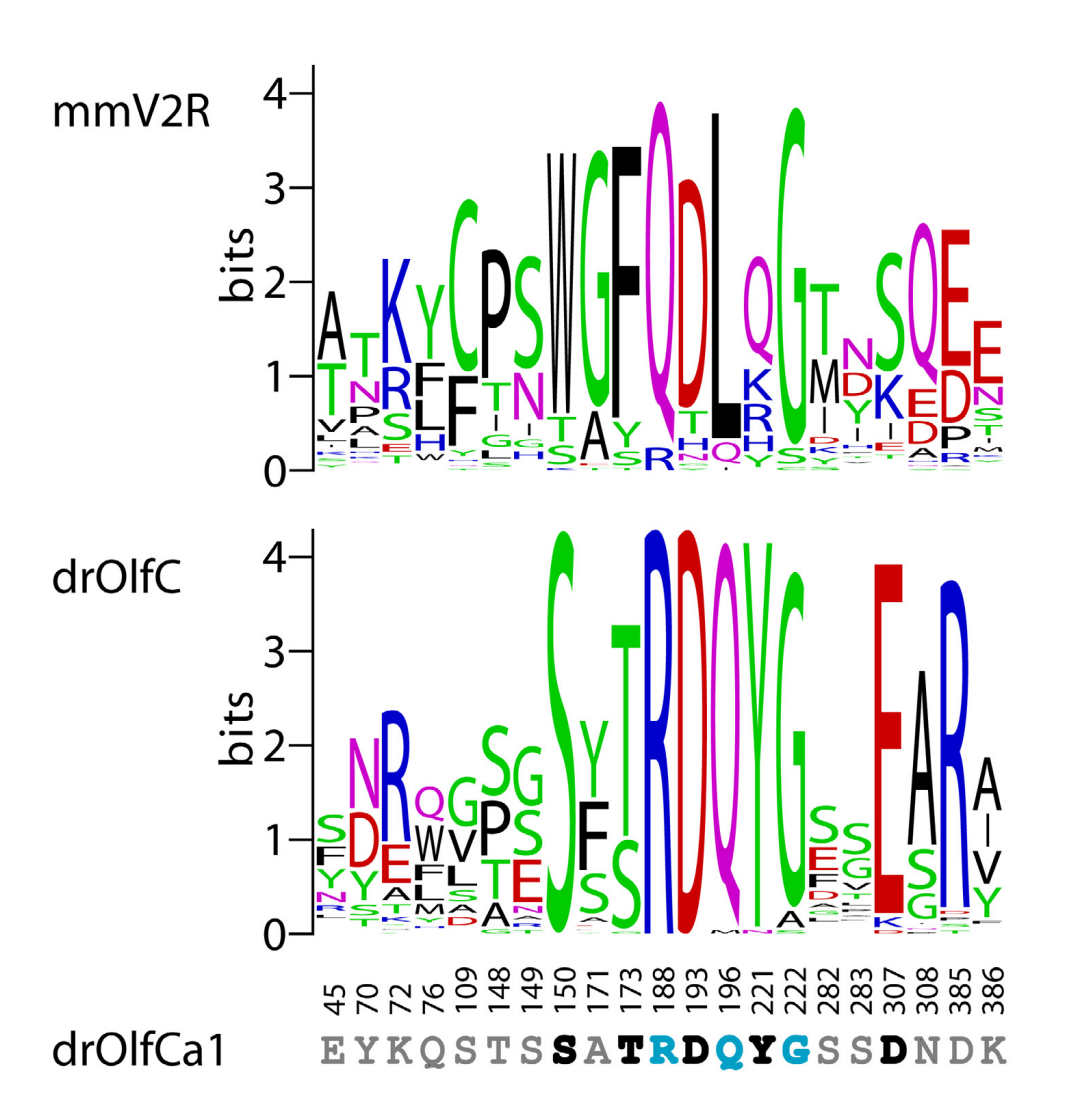
**Conservation of amino acid binding signature in the OlfC family**. Sequence logos were constructed for predicted binding pocket residues in the zebrafish OlfC (drOlfC) and mouse V2R (mmV2R) receptor families, based on an alignment of full-length, intact zebrafish OlfC and mouse V2R amino acid sequences. The over-representation of amino acids at positions equivalent to those predicted to interact with amino acid ligands in zebrafish OlfCa1 [28] is shown graphically. *Y axis*, information content; *X axis*, residue position with respect to OlfCa1 (note that positions are non-contiguous). Conserved proximal pocket signature motif sites that are thought to make contact with the ligand are highlighted in bold black, whereas predicted distal binding pocket sites are shown in grey. Additional signature motif residues thought to play a structural role but not contact ligand directly are shown in bold blue (bottom of figure; see the text for details).

We next examined the patterns of sequence conservation and divergence within the zebrafish OlfC receptor family in order to gain insights into the possible functions of these receptors. A sequence logo created from the alignment of full-length zebrafish OlfC amino acid sequences highlights a number of conserved motifs among members of the family (Figure 4). In addition to the clear conservation of the cysteine residues of the cysteine-rich domain and heptahelical transmembrane (TM) domains (both of which are important structural elements in this class of GPCR), several stretches of residues in the ligand-binding N-terminal domain (NTD) are also conserved. These NTD residues largely correspond to secondary structure elements such as the core alpha helices in lobe 1 and the beta strands in lobe 2, as well as sites predicted to interact with bound ligand (see below and Figure 5). Overall, the NTD shows less conservation than the cysteine-rich and TM domains, consistent with these latter domains' roles in receptor structure and coupling to intracellular signaling pathways [49,50].

What clues about receptor function or ligand specificity might be gleaned from an analysis of sequence conservation within the OlfC receptor family? It is instructive to consider this question in the context of the structure of C family GPCRs. The ligand-binding NTD of C family GPCRs is thought to adopt a conformation resembling a bilobate clamshell-like structure [49,50]. Stabilization of the closed conformation of the clamshell – through interactions between bound ligand and the inner faces of the two lobes (lobes 1 and 2) – leads to receptor activation. For amino acid-binding receptors, the binding pocket formed by the two lobes of the clamshell can be divided into two regions: a proximal pocket, comprising residues that interact with the glycine moiety of the amino acid ligand (α-carboxylate, α-amino and α-proton), and a distal pocket, comprising residues that interact with the ligand's R group side chain [49]. Studies on a wide variety of amino acid-binding proteins have identified a core "signature" motif of 5 residues within the proximal binding pocket that participate in critical interactions with the amino acid ligand ([32,33]; see Figure 5). Thus, the presence of the ligand-binding signature motif in a given receptor would be consistent with the receptor's possible function in amino acid sensing. An additional 3 conserved residues predicted to be involved in structural interactions in the hinge region have also been identified in the amino acid receptors ([32]; see Figure 5). Although still subject to experimental validation, together with the core 5-site motif these latter 3 sites have been proposed to comprise an 8 residue signature of amino acid receptors [32].

We have shown previously [28] that the amino acid-binding core signature motif is present in goldfish receptor 5.24, a Group III OlfC receptor that is activated by amino acids [27]; mutagenesis of any one of the 5 signature motif residues in receptor 5.24 results in a profound decrease in receptor activation by ligand [28,47]. The corresponding 5 amino acids in OlfCa1 (previously named ZO6), the zebrafish ortholog of goldfish receptor 5.24 that is also activated by amino acids, are fully conserved [28]. We were therefore interested in determining to what extent the amino acid-binding signature motif is conserved within the zebrafish OlfC receptor family, and by inference whether the OlfC receptors may constitute a family of amino acid receptors.

**Figure 6:**
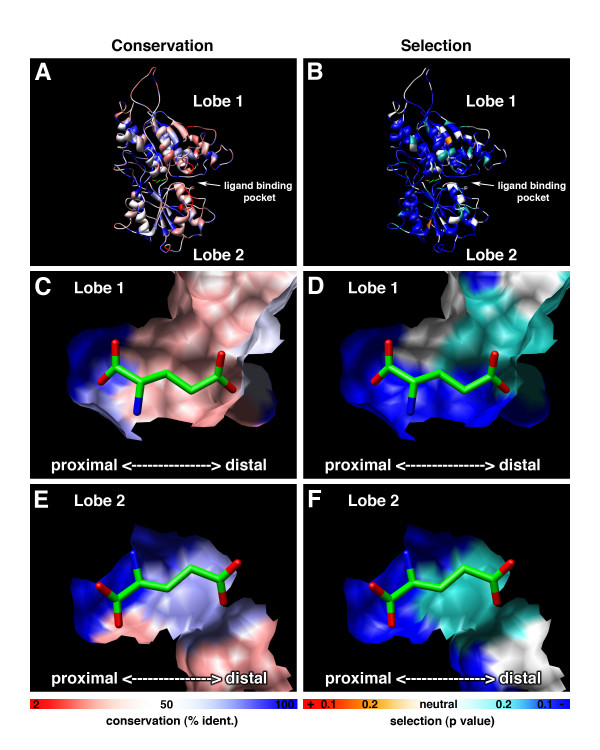
**Amino acid conservation and selective pressure displayed on a structural model of the OlfC N-terminal ligand-binding domain**. Percent identity **(a, c, e) **and inferred selective pressure **(b, d, f) **for the zebrafish OlfC family is displayed on a structural model of OlfCa1 [28]. The extracellular N-terminal domain **(a, b) **is represented by a ribbon. The inner, ligand-binding faces of lobe 1 **(c, d) **and lobe 2 **(e, f) **are shown as surface representations. Bound glutamate – the cognate ligand for OlfCa1 [28] – is shown docked in the binding pocket with the alpha carbon and proximal pocket oriented to the left and distal pocket to the right. Residues are colored by percent identity, from 2% (red) to 100% (blue) **(a, c, e) **or by selective pressure (positive selection shown in red (p < 0.1) or orange (p < 0.2), neutral selection in white, and negative selection in blue-green (p < 0.2) or blue (p < 0.1) **(b, d, f)**.

We addressed this issue using two tools for mapping amino acid substitutions to relevant sites in the receptor structure: (1) a sequence logo representation of residues predicted from our previous structure-function studies [28] to be involved in amino acid binding, including the aforementioned 8 residue signature motif (Figure 5), and (2) a representation of conservation and divergence overlaid on our structural model for the ligand-binding NTD of OlfCa1 (Figure 6; [28]). Three salient features are evident from this analysis. First, the 8 residue signature motif (S150, S/T173, R188, D193, Q196, Y221, G222, D/E307 in OlfCa1 coordinates) is highly conserved in the zebrafish OlfC family (Figure 5), as well as in the fugu OlfC receptors [see Additional file 6]. Similarly, a high level of conservation is seen for residues forming the surface of the proximal binding pocket (Figure 6). These observations suggest that the OlfC receptors as a group are capable of binding to amino acids and their derivatives. Second, a sequence logo of the mouse V2R receptors (Figure 5) shows that, with the exception of the Group II V2R2 receptor(s), the amino acid-binding signature motif is absent from the mammalian V2R receptors. Since the signature motif is strictly required for amino acid binding in all C family GPCRs identified to date [32], Group IV and Group V V2R receptors have likely evolved to receive non-amino acid stimuli; the probable functional divergence of the V2R and OlfC receptors mirrors the phylogenetic divergence of these two receptor families (Figure 3). What, then, are the cognate ligands for the V2R receptors? Recent studies have implicated the V2R receptors in detecting MHC peptides and other conspecific peptide cues [22,51]. We speculate that since such peptides are likely too large to fit within the conventional clamshell binding pocket, they may instead activate the receptor by allosteric interactions on the exterior of the NTD or cysteine-rich domain, similar to the allosteric activation of T1R sweet receptors by peptide sweeteners [52]. It should be noted that fish also appear to choose mating partners based on MHC haplotype, presumably via an olfactory-mediated process [53]; the OlfC receptors may therefore mediate chemoreception of peptide cues via such allosteric interactions, distinct from "orthosteric" ligand binding interactions within the conventional clamshell binding pocket.

Finally, residues predicted to comprise the distal binding pocket – i.e., those interacting with the amino acid's R group side chain – are not conserved within the OlfC receptor family (Figures 5 and 6). Together with the conservation of proximal pocket residues (including those comprising the signature motif), these observations suggest that the OlfC receptor family has evolved to detect and discriminate a diverse spectrum of amino acids and/or amino acid derivatives. Consistent with this multiplicity of putative amino acid receptors, physiological studies have provided evidence for multiple, overlapping pathways used by the fish olfactory system to detect this class of chemical cues [54-56]. Interestingly, behavioral studies in catfish [57] have shown that fish are able to discriminate between amino acids. The future elucidation of the ligand specificities of individual, cloned OlfC receptors should allow an understanding of how this receptor family subserves the physiologic and behavioral discrimination of amino acid-based chemical structures.

### Adaptive evolution of OlfC receptors

Our analysis of sequence conservation within the receptor structure revealed a striking degree of divergence in a considerable portion of the NTD – the region of C family GPCRs responsible for ligand binding [19,50]. While sequence conservation is usually a clear indicator of important function (for example, structural elements necessary for the correct folding of protein domains), lack of conservation is more difficult to interpret. On the one hand, the observed variation might represent relaxed selective pressure consistent with lack of sequence-related function. For example, some loop regions might only function to connect secondary structural elements – their length being important but not their particular sequence. The observed sequence diversity could therefore be the result of genetic drift, with polymorphisms in the population being fixed at a rate consistent with the absence of selective pressure. On the other hand, adaptive evolutionary processes may have driven diversification of protein function (e.g., to allow the recognition of a different chemical ligands by recently-duplicated receptors) via selection on specific residues or protein regions.

We used the relative frequency of non-synonymous vs. synonymous codon substitutions to assess the selective processes acting on the *OlfC *receptor genes [58]. In the absence of positive or negative selection, the number of non-synonymous changes relative to the number of possible non-synonymous changes (dN) is equal to the number of synonymous changes relative to the number of possible synonymous changes (dS) – i.e., dN/dS = 1. Significant deviations of dN/dS from unity reflect selection on the sequence; a dN/dS ratio > 1 indicates that a region has undergone positive selection, whereas a dN/dS ratio < 1 indicates negative or "purifying" selection [58]. For the present analysis, we aligned a set of 50 full-length intact zebrafish OlfC coding sequences and calculated dN and dS values, as previously described [11,58]. We first made these calculations separately for two broad regions of the receptor: the NTD and the combined cysteine-rich plus TM domains (CTD). Both the NTD and CTD appear to be under negative or purifying selection (dN/dS = 0.216 and dN/dS = 0.124, respectively). However, the NTD displays a significantly higher average dN/dS ratio than the CTD (p = 3.02 × 10^-112^). These observations are consistent with a scenario in which there is an overall relaxed negative selection on the N-terminal sequence, which may have permitted OlfC receptors to adapt their binding affinities to different odorants.

What evolutionary processes may have acted on individual sites within the receptor structure? We hypothesize that highly conserved sites within the proximal binding pocket – i.e., those involved in binding the common glycine moiety of all amino acid ligands – might be expected to have undergone negative or purifying selection. Conversely, sites comprising the distal pocket may have undergone positive selection as the receptors evolved to recognize amino acids with different side chains. To test these ideas, we performed a site-by-site analysis of dN/dS ratios using the Single Likelihood Ancestor Counting (SLAC) package [59] (see also [60]), as described previously [11]. A p value derived from a two-tailed extended binomial distribution was used to assess significance at each site. Tests on simulated data (S.L.K. Pond and S.D.W. Frost, methods available in [59]) show that p values less than or equal to 0.1 identify nearly all true positives with a false positive rate generally below the nominal p value; for actual data, the number of true positives at a given false positive rate is lower.

**Figure 7:**
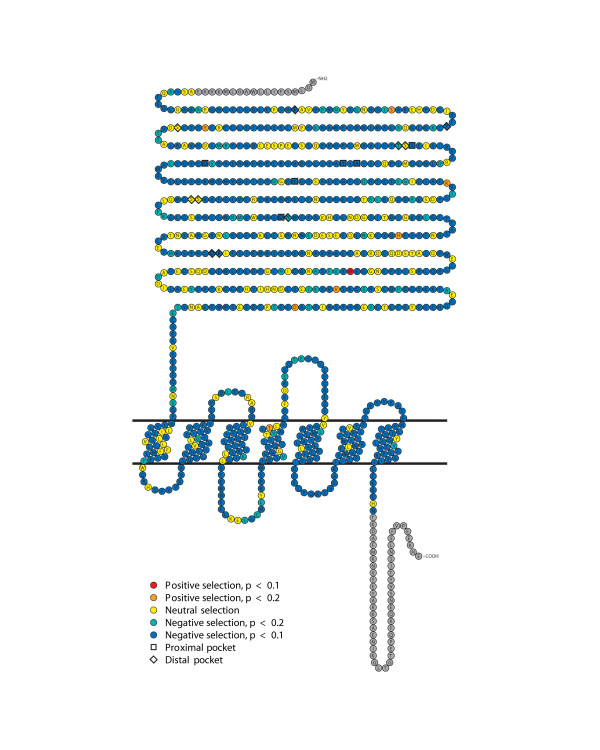
**Sites under positive and negative selection in OlfC coding sequences**. A schematic representation of site-by-site selective pressure is shown on the OlfCa1 receptor sequence. Nucleotide alignments were generated from the corresponding amino acid alignment [see Additional file 4]. SLAC analysis shows the probability of sites being under selective pressure (positive selection shown in red (p < 0.1) or orange (p < 0.2), neutral selection in yellow and negative selection in blue-green (p < 0.2) or blue (p < 0.1). The null hypothesis is that a site is neutrally evolving with dN/dS = 1.

Figure 7 shows the probability for each codon site being under positive or negative selection (dN/dS values different than dN/dS = 1.0) on a snake plot of a representative OlfC amino acid sequence, OlfCa1. By this analysis, only one site, M436 in OlfCa1, appears to be under positive selection (dN/dS >1 with p < 0.1). It is unclear what role this position may play in receptor function. Based on existing models of C family GPCR structure [49,61], this site resides within or near the hinge of the ligand-binding clamshell. The apparent positive selection acting on this site may therefore indicate its importance in modulating some aspect of ligand binding or receptor activation. It is also possible that the identification of this site represents a false positive from this analysis.

Most sites in the receptor appear to be under negative selection (dN/dS < 1 with p < 0.1), including all of the identified proximal binding pocket sites. Interestingly, many (but not all) of the predicted distal pocket sites appear to be under relaxed or neutral selection (Figure 7). These results are also shown overlaid on the structural model for OlfCa1 ([28]; Figure 6). In this representation, surface residues predicted to interact with the bound ligand's glycine moiety show clear evidence of negative selection (dN/dS < 1 with p < 0.1), whereas those lining the distal pocket show a relaxation of this negative selection (p > 0.1). Together these observations are consistent with the hypothesis that the proximal pocket sites are under strong purifying selection, which has maintained the ability of the OlfC receptors to bind amino acid ligands. The apparent relaxation of negative selection on distal pocket sites may reflect the adaptation of these receptors to recognize different amino acids or their derivatives.

Overall, our site-by-site analysis of dN/dS ratios reveals a striking absence of sites exhibiting signs of positive selection. This may be due to the dominating influence of negative or purifying selection throughout the receptor coding region. Alternatively, since non-synonymous substitutions are thought to occur only sporadically over evolutionary time, the signatures of less recent substitutions may no longer be evident. In addition, the power to detect adaptive evolutionary events at the level of individual codons decreases in proportion to the time since the event occurred due to saturation by synonymous substitutions. Thus, our inability to detect such events may indicate that they occurred early in the evolution of this gene family.

## Conclusion

We describe here a comprehensive analysis of the OlfC receptor family – the repertoire of C family GPCRs expressed in the zebrafish olfactory system. Fifty four intact genes comprise this family, which by phylogenetic analysis is distinct from other C family GPCRs. A comparison with OlfC receptors identified in fugu suggests that the 25 present-day subfamilies identified in zebrafish and fugu probably existed in the most recent common ancestor of the cyprinid and pufferfish lineages. Interestingly, the major group of fish OlfC receptors is distinct from the mammalian vomeronasal V2R receptors, suggesting that these two groups of genes evolved to accommodate different chemosensory cues and/or physiological functions in the fish and mammalian lineages. Consistent with this notion, our analysis of sequence conservation and selective pressure indicates that the zebrafish OlfC family retains a binding pocket signature motif common to all amino acid receptors characterized to date [32,33]; this signature motif is similarly conserved in the fugu OlfC receptors. Thus, the fish OlfC receptors likely evolved to allow the detection and discrimination of amino acids, which are potent olfactory cues for teleost fish [29-31]. By way of contrast, the amino acid-binding signature motif is not present in the vast majority of V2R receptors, suggesting that the mammalian V2R receptors became specialized to detect chemosensory cues of a chemical composition different from amino acids. The present results lay the foundation for future studies aimed at elucidating the ligand specificities and structure-function relationships of individual OlfC receptors.

## Methods

### Iterative data mining

Genome-wide searches of the second (Zv2) and third (Zv3) draft zebrafish genome assemblies [34] were performed several times using the predicted OlfCs from each previous round to increase our querying power. The final set of genes was mapped to the Zv6 assembly. Additional BLAST searches of Zv6 yielded no additional genes.

Briefly, the data mining protocol we employed is outlined as follows. Original protein queries for TBLASTN search (e-value cutoff of 1e-4) of the assemblies included goldfish 5.24 (AAD46570), zebrafish Zo6 (AAN19854), fugu Ca09 (BAA26124), fugu Ca12 (BAA26125), fugu Ca13 (BAA26126), goldfish GFB1 (AAC64075), and goldfish GFB8 (AAC64076). Gene prediction on the resulting sequences was performed using Genewise using a hidden Markov model (HMM) constructed using HMMER from the query sequences mentioned above. Protein translations of the predicted genes were aligned with ClustalW. In order to determine appropriate start and stop codons as well as correct mis-predicted intron-exon junctions, we visually inspected the genomic sequence in conjunction with the alignment of the translations. High quality predictions, defined as unambiguous well-aligned splice junctions and ungapped genomic sequence, were used in the next iteration (TBLASTN, HMM construction, Genewise prediction, hand annotation). Final mapping of genes to the Zv6 assembly was performed using Exonerate 1.0 [62] to align the predicted CDS sequences against the genome sequence.

### Alignment and tree construction

For multiple alignments of OlfC genes, MAFFT [63,64] was run with the "localpair" option (all pairwise local alignment information is provided to the objective function) and a maximum of 1000 iterations. Gaps were inspected manually and edited in XCED. Both MAFFT 5.0 and XCED are available in [65]. The sequence segments corresponding to the signal peptide (up to the first conserved cysteine residue) and C-terminal tails were trimmed for all alignments. The neighbor-joining algorithm [66] as implemented by PFAAT [67] (see also [68]) was used to generate unrooted phylogenetic trees from these alignments using the BLOSUM 50 similarity matrix; positions with greater than 50% gaps were excluded. One thousand bootstraps were performed to assess the support at each tree node. Maximum likelihood analysis was carried out using PHYML [69] (see also [70]) on the same processed amino acid alignments described above. Bootstrap analysis with 100 replicates was carried out using the JTT model of amino acid substitution. The consensus tree including bootstrap support for each node was plotted for each dataset using either ATV [71] (see also [72]) or *unrooted *[73] (see also [74]). Sequence logos were generated using the program WebLogo [75].

The C family GPCR amino acid sequences used for comparison to the zebrafish genes predicted in the present study included the set of intact mouse V2R vomeronasal receptors [18], a set of intact fugu C family GPCRs identified from version 4 of the Joint Genome Institute (JGI) fugu predicted protein set (JGI protein IDs: 594197, 744222, 594233, 581784, 584633, 589261, 716738, 571614, 744432, 611619, 611624, 611613, 735220, 179742, 557085, 557101, 602488, 602640, 128843, 581424, 572766, 715887, 556422, 618162, 577965, 559750, 710708, 709657; see below for details), and the following sequences from Genbank: mouse gamma-aminobutyric acid (GABA-B) receptor 1 (NP_062312.2); mouse metabotropic glutamate receptors mGluR1 (NP_058672), mGluR3 (NP_862898), mGluR4 (NP_001013403), mGluR5 (XP_149971), mGluR7 (NP_796302) and mGluR8 (NP_032200); mouse T1R taste receptors T1R1 (NP_114073), T1R2 (NP_114079) and T1R3 (NP_114078); mouse GPRC6A (NP_694711); mouse calcium-sensing receptor CaSR/GPRC2A (NP_038831); goldfish odorant receptors 5.24(AAD46570), 5.3 (full-length sequence corresponding to AF158964), GFB1 (AAC64076) and GFB8 (AAC64076); fugu putative pheromone receptors Ca02.1(BAA26123), Ca09(BAA26124), Ca12(BAA26125), Ca13(BAA26126) and Ca15.1(BAA26127); fugu calcium-sensing receptor CaSR(BAA26122); zebrafish metabotropic glutamate receptors mGluR1 (CAH68968), mGluR2 (XP_692887), mGluR3 (XP_693759) and mGluR5a (XP_696823).

The additional unpublished fugu C family GPCRs were identified by performing a BLASTP search of the JGI protein build with each zebrafish OlfC protein sequence using an E-value threshold of 10^-6^. After filtering out protein sequences less than 700 amino acids in length, 33 remained. These were then searched against the 6 published full-length protein sequences from fugu in order to remove redundant sequences. Twenty eight novel sequences remained and were included along with the six published sequences in our analyses. Note that the JGI genes were predicted by the gene prediction programs Genscan, Fgenesh and Genewise, and have not been subjected to rigorous annotation criteria.

### dN/dS analysis

The dN/dS ratios for multi-codon regions (i.e. the transmembrane domain and extracellular domain) of the OlfC receptor coding sequence were determined using previously published methods [58]. To make inferences about selective pressure (positive and negative selection) on individual codons (sites) within the coding sequence of the zebrafish *OlfC *genes, the Single Likelihood Ancestor Counting (SLAC) package [59], which implements the Suzuki-Gojobori method [60], was used. Fifty out of the 54 intact zebrafish genes (omitting OlfCr1, OlfCq17, OlfCm1 and OlfCg6) were used for all calculations. Details regarding both of these methods are provided in ref. [11].

### Tertiary structure prediction

A model of the zebrafish OlfCa1 receptor NTD [28] was used for structural predictions. Based on this structural model, molecular graphics images were produced using the Chimera package from the Computer Graphics Laboratory, University of California, San Francisco [76] (see also [77]).

### Sequences

Conceptual translations of the zebrafish genes described in this study are provided in Table S3 [see Additional file 7].

## Authors' contributions

TSA carried out the analysis. Both authors participated in the design of the study and writing of the manuscript.

## Supplementary Material

Additional file 1 Supplementary table and figure legends. Table and figure legends describing the information contained in Additional files 2, 3, 4, 5, 6, 7. Click here for file

Additional file 2 Table S1. The zebrafish OlfC repertoire. Click here for file

Additional file 3 Table S2. The conserved intron-exon structure of zebrafish OlfC genes. Click here for file

Additional file 4 Figure S1. Multiple sequence alignment of predicted zebrafish OlfC amino acid sequences. Click here for file

Additional file 5 Figure S2. Schematic representation of predicted OlfC gene structure. Click here for file

Additional file 6 Figure S3. Multiple sequence alignment of predicted fugu OlfC amino acid sequences. Click here for file

Additional file 7 Table S3. Conceptual translations of zebrafish OlfC, CaSR and T1R genes. Click here for file
