# Supplementary material for: The repertoire of olfactory C family G protein-coupled receptors in zebrafish: candidate chemosensory receptors for amino acids
Source: BMC Genomics. 2006 Dec 8;7:309. doi: 10.1186/1471-2164-7-309 (PMC1764893; doi:10.1186/1471-2164-7-309)
Supplement: Additional file 1 — Supplementary table and figure legends. Table and figure legends describing the information contained in Additional files 2, 3, 4, 5, 6, 7. [file 1471-2164-7-309-S1.pdf]

Supplementary table and figure legends

Table S1 [Additional file 2]. The zebrafish OlfC repertoire. Subfamilies were determined by examination of the maximum likelihood tree produced by PHYML and selection of monophyletic clades that conform to an approximate 65% identity cutoff. Partial sequences were assigned manually to subfamilies based on subsequent alignments, usually according to best alignment score. Putative pseudogenes are indicated with the suffix "P." Also provided in this table are the Genbank accession numbers, identity with previously published C family GPCR sequences and OlfC-encoding ESTs, classification as gene or pseudogene, classification as full-length or partial sequence, the number of coding sequence disruptions, Zv6 scaffold coordinates, Zv6 chromosomal coordinates, strand, and results of RNA in situ hybridizations on zebrafish olfactory epithelium (*nd*, not determined). Genes marked with an asterisk (*) were not identified in the present study but reported by Hashiguchi and Nishida (2005).

Table S2 [Additional file 3]. Conserved intron-exon structure of zebrafish *OlfC* genes. Alternating exon lengths and intron phases are shown for each *OlfC* gene.

Figure S1 [Additional file 4]. Multiple sequence alignment of predicted zebrafish OlfC amino acid sequences. Amino acid sequences were derived from the CDS sequences defined by the predicted translational start and stop codons. Conceptual translations were also made for pseudogenes with frameshifts and/or stop codons; an "X" indicates either a gap, frame shift, or a stop codon. Residues that are identical to the consensus (at least 50% identity required for consensus) are shown as white on black, while those that are similar to the consensus according to the BLOSUM62 matrix are shown as black on grey. Non-conserved residues are shown as black on white. The start

and end of the alignment used for tree construction and analysis of selective pressure are indicated (“clip” with arrows). Proximal pocket residues are marked with asterisks (*) and distal pocket residues are marked with plus signs (+). Transmembrane domains are labeled TM1-7.

Figure S2 [Additional file 5]. Schematic representation of predicted OlfC gene structure. Exons are depicted as rectangles and are colored according to the domains for which they code. The regions corresponding to the signal peptide, N-terminal ligand-binding domain, the Cysteine-rich domain, and C-terminal domain are indicated. Numbers between exons indicate conserved intron phases.

Figure S3 [Additional file 6]. Multiple sequence alignment of predicted fugu OlfC amino acid sequences. Amino acid sequences were obtained from the Joint Genome Institute’s protein predictions for the FUGU v4.0 assembly and from Naito et al. (1998) . The JGI genes were predicted by the gene prediction programs Genscan, Fgenesh and Genewise, and have not been subjected to rigorous annotation criteria. Residues that are identical to the consensus (at least 50% identity required for consensus) are shown as white on black, while those that are similar to the consensus according to the BLOSUM62 matrix are shown as black on grey. Non-conserved residues are shown as black on white. The start and end of the alignment used for tree construction and analysis of selective pressure are indicated (“clip” with arrows). Proximal pocket residues are marked with asterisks (*) and distal pocket residues are marked with plus signs (+). Transmembrane domains are labeled TM1-7.

Table S3 [Additional file 7]. Conceptual translations of zebrafish OlfC, CaSR and T1R genes. Conceptual translations of the zebrafish receptor gene sequences described in this study are provided in FASTA format.
