# Supplementary material for: The repertoire of olfactory C family G protein-coupled receptors in zebrafish: candidate chemosensory receptors for amino acids
Source: BMC Genomics. 2006 Dec 8;7:309. doi: 10.1186/1471-2164-7-309 (PMC1764893; doi:10.1186/1471-2164-7-309)
Supplement: Additional file 3 — Table S2. The conserved intron-exon structure of zebrafish OlfC genes. [file 1471-2164-7-309-S3.pdf]

Table S2

Gene Name	Exon 1 length	Intron 1 phase	Exon 2 length	Intron 2 phase	Exon 3 length	Intron 3 phase	Exon 4 length	Intron 4 phase	Exon 5 length	Intron 5 phase	Exon 6 length
OlfCa1	203	2	298	0	759	0	204	0	124	1	1016
OlfCb1P	197	2	292	0	756	0	204	0	118	1	1102
OlfCc1	242	2	292	0	810	0	228	0	124	1	1094
OlfCd1	221	2	289	0	789	0	228	0	124	1	899
OlfCd2	218	2	292	0	786	0	228	0	124	1	899
OlfCd3	221	2	292	0	786	0	228	0	124	1	914
OlfCe1P	200	2	286	0	786	0	225	0	124	1	898
OlfCf1			144	0	804	0	228	0	124	1	905
OlfCg1	209	2	289	0	756	0	228	0	124	1	917
OlfCg10	209	2	289	0	759	0	228	0	124	1	914
OlfCg11	206	2	289	0	753	0	228	0	124	1	905
OlfCg12	209	2	289	0	759	0	228	0	124	1	905
OlfCg2	200	2	289	0	756	0	228	0	124	1	911
OlfCg3	209	2	289	0	756	0	222	0	127	1	911
OlfCg4	209	2	289	0	756	0	228	0	124	1	902
OlfCg5	209	2	289	0	750	0	228	0	124	1	902
OlfCg6	206	2	289	0	756	0	228	0	124	1	911
OlfCg7	209	2	289	0	750	0	228	0	124	1	911
OlfCg8	209	2	289	0	738	0	228	0	124	1	911
OlfCg9	209	2	289	0	756	0	228	0	124	1	914
OlfCh1	203	2	289	0	822	0	228	0	124	1	896
OlfCj1	206	2	286	0	813	0	231	0	124	1	914
OlfCk1	230	2	295	0	786	0	222	0	124	1	914
OlfCk2	230	2	295	0	786	0	222	0	124	1	914
OlfCk3	230	2	295	0	786	0	222	0	124	1	914
OlfCm1	215	2	292	0	792	0	228	0	124	1	908
OlfCm2	215	2	292	0	792	0	228	0	124	1	
OlfCn1	203	2	286	0	822	0	222	0	124	1	911
OlfCq1	227	2	292	0	804	0	228	0	124	1	905
OlfCq10P	224	2	292	0	828	0	201	0	124	1	914
OlfCq11	221	2	292	0	813	0	225	0	124	1	914
OlfCq12	221	2	292	0	810	0	228	0	124	1	914
OlfCq13	218	2	292	0	810	0	228	0	124	1	911
OlfCq14	224	2	286	0	816	0	228	0	124	1	899
OlfCq15P	327	0									
OlfCq16	215	2	292	0	828	0	228	0	124	1	914
OlfCq17	221	2	292	0	828	0	228	0	124	1	914
OlfCq18	221	2	292	0	822	0	228	0	124	1	914
OlfCq19	221	2	292	0	828	0	228	0	124	1	914
OlfCq2	215	2	289	0	828	0	225	0	124	1	914
OlfCq20	224	2	292	0	822	0	228	0	124	1	914
OlfCq21	221	2	292	0	828	0	228	0	124	1	914
OlfCq3	209	2	292	0	804	0	228	0	124	1	911
OlfCq4	224	2	292	0	828	0	225	0	124	1	914
OlfCq5	224	2	292	0	828	0	225	0	124	1	914
OlfCq6	221	2	292	0	810	0	228	0	124	1	908
OlfCq7P									124	1	845
OlfCq8	221	2	292	0	810	0	228	0	124	1	908
OlfCq9	224	2	292	0	828	0	228	0	124	1	914
OlfCr1	251	2	277	0	831	0	210	0	124	1	902
OlfCs1	221	2	292	0	819	0	231	0	124	1	896
OlfCs2	206	2	292	0	819	0	231	0	124	1	899
OlfCt1	224	2	286	0	819	0	222	0	124	1	905
OlfCu1	227	2	307	0	816	0	228	0	124	1	905
OlfCv1	200	2	289	0	810	0	225	0	124	1	902
OlfCv2	197	2	289	0	810	0	225	0	124	1	902
OlfCv3	209	2	289	0	816	0	225	0	124	1	899
OlfCw1	221	2	289	0	837	0	231	0	124	1	899
OlfCx1	227	2	298	0	834	0	222	0	124	1	896
OlfCx2	227	2	298	0	825	0	222	0	124	1	896
OlfCx3	227	2	298	0	825	0	222	0	124	1	896
OlfCy1P											525
