# Supplementary material for: The repertoire of olfactory C family G protein-coupled receptors in zebrafish: candidate chemosensory receptors for amino acids
Source: BMC Genomics. 2006 Dec 8;7:309. doi: 10.1186/1471-2164-7-309 (PMC1764893; doi:10.1186/1471-2164-7-309)
Supplement: Additional file 4 — Figure S1. Multiple sequence alignment of predicted zebrafish OlfC amino acid sequences. [file 1471-2164-7-309-S4.pdf]

```
                                                         clip
                                                          |                                +                        +
OlfCd2     1 M-------ANTLSLLLLLLLHGHFL------PAT-VQICD-LV--GQSALPVL--SAERDINIGAIFSIH-RNALLRLY---PFTSKPEPTTCV-RLNLREFKFAQTFIFAIEEINNNT
OlfCd3     1 M------AKKIVSLPVLLLIVYGISV------SAL-AQVCR-LL--SQPALPLL--SAERDINIGAIFSIH-ISALLKMH---SFTFKPESTACI-SLSLREFKFAQTLIFAIEEINNNT
OlfCd1     1 M------TKKSELLLLMVLVTHGICV------PNL-AQVCR-LL--GQPALPLL--SEERNINIGAIFSLH-KSALLKSH---NFTSKPKQTTCG-GLNLREFKFVQTLIFAIEEINNS-
OlfCf1     1 --------------------------------------------------------------------------------------------------------------------
OlfCe1P    1 M-------------VFLHTWLFFTLV------RAA-NPPCK-LL--GPPNIPQF--SKDGDVTIGGIFSFH-NSWEEIMP---TFTSKPEQPKCK-SLSLREFQNAQTMIYAIEEINKRA
OlfCm1     1 M--------EPLFALIHVVMNIISFA------KAN-ETACT-LQ--GQPVYPQL--WKDGDIIIGGVFSFH-SSWEIRQL---TYSVTPPPLKCI-SLNYRDFQYVQSMLFAVEEINNSS
OlfCm2     1 M--------EPLVALLHMVIAIMTFS------KAN-ETTCT-LQ--GEPVLPEL--WKDGDIIVGGVFSFH-SSWEVRQL---TYTFVPPPLKCI-SLNFRDFQYAQSMLFAIEEINNSS
OlfCg9     1 M----------LLYLCTLLLFFKLKV------SVG-NASCK-IM--GEPKYPLL--FKDGDVTIGALFPVH-SIETAPSF---EFTQKPQLLSCS-SVNLRDFRLAQILIFAIEEINRSE
OlfCg7     1 M----------ILYFYTILFLRCFQA------KTE-NAFCQ-II--GEAKYPLL--SKDGDVTIGGIFAIH-SKETLPSF---EFQQKPQPLLCS-SVNLRDFRLAQIMIFAIEEINKSE
OlfCg1     1 M----------LLFLYIFLHFTLIRT------KAG-NGLCQ-MM--GDPKYPLF--SKDGDITIGALFAIL-SKETLPSF---QFTQKPQLLSCS-SVNLRDFRMTQIMIFAIEEINRSK
OlfCg5     1 M----------LILLSIFLLFNEPFT------KAE-DTICQ-KM--GDPKYALF--SKVGDVTIGGIFSIR-SVEILPSL---EFTQKPQLLSCS-SVNLRDFRMAQIMIFAIEEINRSE
OlfCg12    1 M----------SVFFCTLLIFFQLYA------KAE-KPICI-MM--GDPKYPLL--SKEGDISIGAVFPVH-SIETLPLF---KFTQKPQLLSCS-SVSIRDFRMAQIMAFAIEEINRNE
OlfCg11    1 M----------SVFLYTVLIFI-FYT------KAE-NPLCQ-MM--GNPKFPLL--SKDGDVNIGAIFSVH-STEILTSF---TYTQKPQLLSCS-SVSLRDFRMVQTMIYAIEEINRSL
OlfCg6     1 M-----------FLFYTILLFHLLHT------KVE-NSLCR-IM--GDSNYPLF--SKNGDVSIGGIFAIH-RKETLPSF---EFMQKPQPLSCS-SVNLRDFRLAQTMIFAVEEINRSK
OlfCg2     1 M----------LFFLHTLPLLYQLNT-----------NTPCQ-TM--GDPKDPLF--SKDGDVTIGGVFAIH-RKETLPSF---EFAQKPLPLSCS-SVNLRDFRLAQIMIFAIEEINKSK
OlfCg10    1 M----------VSFIWTLLVVPLQA------KAE-NSLCR-MM--GQNINPLI--SKEGEVTIGALFPIH-SIEILPSF---EFTVKPQLLSCS-SVNLRDFRMAQTMTFAIDEINKNQ
OlfCg4     1 M----------LLFIYILLLFHQLHT------KAG-NTQCR-IM--GDSTYPLI--SKNGDITIGALFPIH-STETLTSL---KFTRKPQPISCS-SVNLRDFRLAEIMIFAIEEINKSE
OlfCg3     1 M----------LLFLYTLLLFHHLHT------KAG-NTLCR-IM--GDPQYPLL--STDGDITIGALFPVH-STETLPSF---EFTKQPQLLSCS-SVYLRDFRLAQILIFAIEEINRSV
OlfCg8     1 M----------LLFLYSVLSLYHLNT------KAE-NTLCQ-MI--GNPKYPLL--SKDGDITIGALFVIH-SEVSLPSF---AFTQKPNLLSCS-SLNIKDFRLAQTMIFAIEEINKNT
OlfCj1     1 M-----------LLLATILTTIACTL------SAA-EPECE-AY--MTDELLYF--SKEGNVSIGGVFSFH-QNPVGVNP---TLRTNPGNIRCN-GLDPGELQYAITMIFAIEEINNRT
OlfCh1     1 M------------FGFGFLLLCSIKA------KEV-ESNCN-MI--GKPENPLL--SQDGDIIIGGAFSIH-NKINLIIP---SFTEKPHHLMCT-SLNLRELHFAQTMIFAIEEINSKR
OlfCk3     1 M----YNNVLLIVTLQCIITRLYVVY------GNR-LGSCI-LQ--GDPQPPVL--FDEGDFIIGGAFSIH-YYLRTEKH---TYTMRPQSLECSGSMDFRELRFARVLQFAIQEINNSS
OlfCk1     1 M----HVNLLLILTLLCIRRLFPAVC------GVH-LGTCI-LQ--GDPQPPSL--FSKGDFVIGGTFTIH-YYLRTEKR---TYTVRPQPLMCSGSMDFRELRFARVLQFAIQEINNSS
OlfCk2     1 M----NAHLLFTVKMLCIARLCIAVC------GVD-LGTCI-LQ--GDAQPPAL--SEDGDFIVGGAFTIH-YYVRTEKY---TYTRRPQPLECSGSMDFRELRFARALQFAIQEINNSS
OlfCq19    1 M------WVILNISIYLIFNFIMSAS------ILG-SNTCQ-LQ--GHFRLNEM--YHDGDVILGGLFEVD-FLTVFPDL---DFKTEPEPPYCV-QFDMESFQQAQTMAFAIDEINKNP
OlfCq17    1 M------CITLYLSLCLCFKHIYADS------IHR-LRSCQ-LQ--GRFKLNGM--YQDGDFILGGLFHVH-FFTVFPEL---SFRMEPQQPYCE-KFNMEGLQHAQTMAFAINEINKNP
OlfCq16    1 M------WTTLYI--FLLFRCFSSES------ALR-SGSCQ-LQ--GRFKLEGM--YQDGDFILGGLFHVH-FFTVFPEL---SFQTEPEPPYCE-KFNIEIFQQAQTMAFAVDEINRNP
OlfCq20    1 M------WITLCIYLYLSLNYICAHS------ILRTSDSCR-LQ--RRFHLNGM--YKDGDVILGGLFQVH-FFTVFPEQ---SFRTEPEPPFCE-KFDMESFQQARTMAFAIEEINKNP
OlfCq21    1 M------WISLNIFLYLSLNCIFAAS------VVN-PGTCQ-LQ--GHFKLNGM--YQDGDFILGGLFEVH-FFTLFPKL---SFKSEPEPPYCE-KFDMESFQQAQTMVFAIDEINKNP
OlfCq18    1 M------WFTLYIYICLSLNCISATL------ILK-PSMCL-LQ--RSFKLNGM--YQDGDFILGGLFEVH-FFTVFPDL---TFTTNPKSPYCE-IFNMEGFQHAQTMAFAIEEINKNP
OlfCq8     1 M------WITLLINIYLILKCISAAV------VLR-AGACQ-LQ--GRFRLNGM--YQDGDVILGGMFEAH-FFTLYPEL---TFRTEPAPPYCE-IFSMESFQNVQTMAFAINEINMNP
OlfCq6     1 M------WITLLINIHLILKCISAAV------VLR-AGACQ-LQ--GRFRLNGM--YQDGDVILGGLFEAH-FFTLFPEL---TFRTEPAPPYCE-IFNMESFQYAQTMAFAINEINRNP
OlfCq13    1 M------WSPLIICLYLSYNFKSAAS------IL--MGSCQ-LQ--GHFGLNGM--YQAGDVILGGLFEVH-LLAVFPEL---SFRSAPEPPYCE-QFDMASFQQAQTMVFAIDEINKNP
OlfCq14    1 M------WIILNIFLCLS-KWISADS------VLR-LDTCQ-LQ--GHFMLNGV--YKHGDVILGGLFDVH-LLTVFPQL---SFRVQPKPPYCE-KQVMENFKSSQTMAFTIDEINNNP
OlfCq12    1 M------WIIAKICLYLSFSCISVAS------IFR-SGPCQ-LQ--GQFRLNGM--FQEGDLILGGLFEVH-FLTVFPEQ---SFRTEPEPPYCE-QFDMASFQQAQTMVFAIDEINRNP
OlfCq1     1 M----DTWITLYLCFYLFFKSISASS------ISK-SGSCQ-LQ--GHFKLNGM--YQDGDLMIGGLFAFH-LITVFPEL---NFKREPEQTHCE-RFYMASFQQAQTMVYAINEINNNP
OlfCq10P   1 M------WAILLFCLFLSCNYICVTL------MVS-SGTCQ-QQ--GHFTLNGM--HQDGDFVIGGLFEIQRYLKVYPEI---SFRTQPNLPNCE-LFYMTSFQQALTMVFAISEINSNP
OlfCq9     1 M------WAILLFCLFLSCNFICVTL------MVS-SGTCQ-KQ--GHFTLNGM--HQDGDFVIGGLFEIQSYVKVYPEI---SFRTQPKLPNCD-LFYMTSFQQALTMVFAISEINNNP
OlfCq5     1 M------WAVLLFCLVLSCCYICVTL------IAS-TGTCQ-QQ--GHFTINGM--HQDGDFVIGGLFDVQTYLKVYPEI---SFRTQPKLPNCE-LFYMTSFQQALTMVFAISEINHNP
OlfCq4     1 M------WAVLLFCLVLSCYYICVTL------MAS-TGICQ-QQ--GHFTINGM--HQDGDFVIGGLFDVQTYLKVYPEI---SFRTQPKLPNCE-LFYMESFQQALTMVFAISEINHNP
OlfCq7P    1 --------------------------------------------------------------------------------------------------------------------
OlfCq11    1 M------WVILHIYFFISCNYMSVAL------MAS-SGTCQ-LQ--GHFTLNGM--YQNGDFLIGGLFEIQ-YLKAFPGL---SFRTEPKLPHCE-NFYMTSFQQAITMVFAINEINNNP
OlfCq3     1 M------CITLNICLHLSFIFIS----------VN-SDSCQ-IQ--GHFKLNRM--YKDGDYIIGGLFEVQ-HLKVFPEL---SFRMEPEQPKCE-EFYMSSFQQAQTMVFAIDEINKNP
OlfCq2     1 M------WITLNICLYMYFNHIS--G------VFT-SESCQ-LQ--GCFKLNGM--YQDGEFIIGGLFEVQ-NLKVFPEL---SFTNEPELPQCE-EFYMASFQQALTMVFAINEINSNS
OlfCr1     1 M------SIHVLSWLTLSVPVLLLWPVCGSGTEVTPLETCVYLITPQYSDELGT--YQDGDVIIGGLINLH-NLAATPDL---SFTRKPGLAQCL-EFQERTYRWFQAIVFTVEEINQNP
OlfCt1     1 M------AFLRLYLFLTVLLFSFFIS------SQL-PQICRLLR--RTDDLPVL--VSEGDIMIGALFPLH-DTILESPP---SFTTEPHPTQCS-GFNFRTFRWMQTLIFAIEEINRDK
OlfCs2     1 M-----------QLWIVGLIGEIIRL------CST-EQSCG-LQ--GRVISESL--YKEGDVIIGGLFPVY-NEAPVPNH---AFTQIQNRSRCQ-GVDLRSYRWLKTMLFTVEEINRDP
OlfCs1     1 M------MGFIIGLWLVGFIVGMVRV------CNA-NPTCS-LK--KSFVSESL--YKDGDVIIGGLFPVH-VQSPVPDP---AFTQQQHGSNCQ-GIDLRSYRWLRAMIFTVDEINRDP
OlfCv3     1 M----------TILLLWLLVYHLSVLN------PAQ-ASKCL-LQ--NEFE-PGL--MANGDFVIGGIFPLH-YNQEMPDL---NFTYKPGPVKCN-GFDTRAFRWAITMKLAVEEINKRV
OlfCv2     1 M--------------IYIFMSIFHLSS------ASM-ALICS-PH---DFDLPVF--MTSGDFTIGGIFPLH-YRVKLPPT---DYMKKPLTAQCR-GFDPRAFRWALSMKLAVEEINNRK
OlfCv1     1 M--------------IHIFLCIFHLSS------AAM-TSRCI-PR--GNFDLPVF--MTSGDFTIGGIFPLH-YRVELPPT---DYMKKPLTAMCR-GFDPMAFHWALTMGLAVEEINNRK
OlfCw1     1 M------KTPVLRAIHLVFFLFYPIQ------SNS-EANCK-LW--KELDLTVV--HKEGDVILAGMFPIH-SKGIDQEL---NFRNQPDQRKCW-GFNMRVFRWSQAMIFFIEEINRNP
OlfCn1     1 M------------KNFNILLFCSLLY------VQT-SSMCQ-LL--RTFEMPNI--FKVGDIMIGGIFPIF-NKQENIIG---SFERNMQRIKCT-GFDLRAFRWIQSMMFAVDEINKNE
OlfCu1     1 M----PVIELVSLGLALLLNFSVVLC------TSL-DKSCW-TL--GDFNSPVL--EQDGDIVIGGLFPMH-NIAPETDY---NFSDLPHYQDCS-RFDFRAFRWVQTMVFAIEEINSNS
OlfCx3     1 M----AGVYKVLISFWPLLCIGVQSQ------YKS-KENCV-FQ--GDEDTYSF--YQRGDVVLGGIFPLH-SSPVSSLF---SFRTKPKPTSYK-FFTPRALRWMQTMIFAVREINQRQ
OlfCx2     1 M----ALLCATLLMVWFLMYLSVQSQ------YNS-KVRCV-FQ--GDDDTNTF--YQGGDVVLGGLFPLH-FSPISSLS---SYKTKPTPTTYK-FFTPRALRWMQTMIFAVREINQRQ
OlfCx1     1 M----AQIYATLLTLCLLLYIRVQCQ------SNN-KYKCI-YQ--GDDDTYSF--YQGGDLVLGGIFPLH-SSTIPQFF---SFTTKPKLIQYK-FFTPRALRWMQTMIFAVREINQRQ
OlfCc1     1 MDLTGLSYEGRFLLVLCMISYLF--------TPTDAEGSCK-LK--AKFNLRGYKEVEKTTVVIGGMFPVH-RSLVSTDS---NTTDPPESVDCQ-GFNFRAFRWAQTMLFALKEINSRT
OlfCa1     1 M----------DLMSFILLWAGL---------------MKVAEASIAQFSQLGA--SAPGNIIIGGLFPIH-EAVVPVNYTGNNSISAPEHPDC-IRFYTKGLNQALAMINAVEMANKSP
OlfCb1P    1 M---------KAAGQLCVLGLMM-------------LSWVSWLRCDPVDSMCGA--YLNGDVNIAILSSIH-SKVRNL-----HQRTRPQPFIC-SDFDLMTFVQSLGAIHTVEEINNS-
OlfCy1P    1 --------------------------------------------------------------------------------------------------------------------
OlfCq15P   1 --------------------------------------------------------------------------------------------------------------------
```

```
                               +                                              ++*                    + *                   *
OlfCd2       97 QLLP-GVTLGYKIYDACNAISPTLVSGMALINGYEN-----TLSDTSCS--QP-PAVQAIVGESSSSPTTALAALVGPFNIPVISHFATCACLSNRKRYPSFFRTIPSDYYQSRALAQLV
OlfCd3       98 QLLP-GVSLGYKIYDSCGSIAQAIFSGMALMNGYEE-----TLSDTSCS--RP-PAVNAIVGESNSSPTIALASIAGPFSLPVISHFATCACLSNRKRYASFFRTIPSDYYQSRALAQLV
OlfCd1       97 SLLP-GASLGYKIYDSCGSVAQAIFSGMALMNGYEE-----TLSDTSCS--TP-PAVHAIVGESNSSPTIGLASVVGPFSLPLVSHFATCSCLSNRKRFRTFFRTIPSDYYQSRALAQLV
OlfCf1        1 --------------------------XMTLVNNQEQ-----SEPGANCS--KP-QTVQAIIGHSGSTPTMSFAKIVGNFHIPVISHFATCACLSNRKEYPSFFRTIPNDFYQSRALAQLV
OlfCe1P      91 DILP-GLSLGYRIFDSCGSIEMALRASLSLVNGE-------NASHLSCQ--RP-KTVQAIIAETSSTPTIAISATVGPLHLPVISHFATCACLSDRKKHPSFFRTVPSDYYQSRALAKLV
OlfCm1       96 TLIP-GVSLGYRIYDTCGSMAMAVRVTMALANAHEN-----TTSDGPCT--KQ-AYVQTILGDTTSSACMAMAKTIGPFNLPMISHYATCECLSDKVKYPSFLRTITSDYYQSRALAELV
OlfCm2       96 TLLP-GVSLGYKIYDTCGSVAVGVRAAMALANGHEK-----ISVEGPCT--KH-AEVQAILGDTTSSACMAITKSIGPFKLPLISHYATCECLSDKVKYPSFLRTIASDHYQSRALAELV
OlfCg9       94 SLLP-KLSLGYKIYDTCSSKLSSMSATMALMNSFE------FAGRDKCN--GQ-TSVHAIIGETESSATVILTRTTGPFKIPVISHAASCECLSSRKEYPSFFRTISSDYHQGRALAYIV
OlfCg7       94 NLLP-NVSIGYRIFDTCGSRLSSMSATMGLMNELK------FAAGETCN--GQ-SPIHAIIGETESSATVILSRTTGPFKIPVISHSASCECLSNRNDYPSFFRTISSDYHQGRALAYIV
OlfCg1       94 SLLP-NVSIGYRIYDTCGSRMSAMSATMALMNGPE------FTADKICN--GE-SPIHAIIGETESSATIILSKTTGPFKIPVISHSASCECLSNRKIYPSFFRTIASDYHQGRALAFIV
OlfCg5       94 SLLP-NVSIGYKIYDSCGSRLSSISATMSLMNDQE------FPKGNICN--GQ-YPIHAIIGETESSATVILSRTTGPFKIPVISHSATCECLSNRRDYPSFFRTIASDYHQSRALAYIV
OlfCg12      94 SLLP-NVSLGYQIYDTCGSGLSLMGANMALMNVQE------FASRGSCN--GQ-SPVHAIIGETESSNTVILSRTTGPFKIPVISPSASCECLSNRKEYPYFFRTIASDYHQSRALAYII
OlfCg11      93 GLLP-NITVGYQIYDACGSRLSAMSATMALMNGPE------FTWRDRCT--GQ-SPIHAIIGETESSATVILSRTTGPFKIPVISPSATCECLSNRKEYPSFFRTIASDYHQSRALAYIV
OlfCg6       93 SLLP-NVSIGYKIYDTCGSRLSTMTAIMGLMNGQD------FSTEDRCN--GQ-SRLHAIIGESESSATIVLSRTTGPFRIPVISHSSSCECLSNKKDYPSFFRTISSDYHMSRALVYIV
OlfCg2       91 SLLP-NIYIGYRIYDTCGSRLSTMSATMAVLSGQE------FRPRDRCN--DQ-PPLHAILGESESSATVILSRTTGPFKVPVISPSATCECLSNRKDYPSFFRTIASDYHQSRALAYIV
OlfCg10      94 SLLP-NVSIGYRIYDTCGSRLSSMSATMALMNGKE------FSAEDKCN--GQ-SAIHAIIGETESSATVILSRTTGPFKIPVISHSATCECLSNRKNHPSFFRTIASDYHQSRALAYIV
OlfCg4       94 SLLP-NVTIGYQVYDTCGSRLSTMSAIMGLMNSQG------FDAEDGCK--GQ-TPIQAIIGDSESSATVILTRTTGPFKIPVISHSASCECLSNRNDYPSFFRTTASDYHQSRALVHIV
OlfCg3       94 QLLP-NISIGYRIYDTCGSRQSTMSAIMGLMNGQE------FGAGERCN--GR-SPIHAIIGESESSATVILSRTTGPFKIPVISHSASCECLSNKKDYPSFFRTMASDYHQSRALAYLV
OlfCg8       94 NLLP-NISVGYQIYDTCGSRFHSMSATMALMNGPK------NSEGYTCN--EQ-SSVHAIVGETESSNTIILSRTTGPFKIPVISPTATCECLSNRKEFPSFFRTIASDYHQSRALAYIV
OlfCj1       93 DLLP-GFILGYRIYDSCPSIPLSVGASLTLMNGQ-------METKKSCA--SP-SAVQAVIGETTSTSTIDIARTIGPFKIPVLSHSATCACLSNRQQYPSFFRTIPSDYYQSRALVKLV
OlfCh1       92 SLLP-NISIGYQIFDSCGSTLASMRSSMALINGQE------LTAEHTCS--GK-PAVKAIIGESESSTTIVLSRAAGPFNIPVISHFATCACLSSRKQFPSFFRTIPSDYYQSRALAQLV
OlfCk3      101 DLLP-GITLGYRIYDSCGSVPMAVKLSFQLANGLDLI----FNDNDSCS--KS-AAVAALVGESGSTPSISISRLYGPFGIPQVSHYATCACLSDKRQHPTFFRTIPSDHHQAAALARMV
OlfCk1      101 NLLP-GITLGYHIYDSCASVPMVIKVAVQLANGLQLA----FNDTDSCA--QS-SEVLALVGESGSTAAITTSRLFGPFGIPQVSHYATCACLSDKRQHPTFFRTIPSDHHQAAALARMV
OlfCk2      101 DLLP-GIFLGYHIYDSCGSVPMAIKVALQLANGLDPK----YNDTDSCA--KS-AAVLALVGDSASTPAISISRIFGPFEIPQVSHYATCACLSDKRQHPTFFRTIPSDHHQAAALARMV
OlfCq19      98 NLLP-NITLGYHLFDNCVMLGMAFRAAISMASGTEE-----FFNNINCT--GP-PPVIGIVGDSSSTVSIAISSILGLFRVPIVSHYATCSCLSDRKKYPSFFRTIPSDAFQVRAMVQIL
OlfCq17      98 TLLP-NITLGYHLYDNCVMLRMAFRAAMSLASGTED-----SYSNLNCT--GP-PPVIGIVGDASSTPSIAISSVLGLFRVPIVSHYATCSCLSDRKKYPSFFRTIPSDAFQVRAMVQIL
OlfCq16      96 NLLP-NITLGYHLYDNCVRLGIAFRAAISLASGTEE-----SFSNLNCT--GP-PPVIGIVGDPSSTPSIAISNILGLFRVPIVSHYATCSCLSDRKKYPSFFRTIPSDAFQVRAMIQIL
OlfCq20      99 NLLP-NITLGFHLYDNCVRLGMAFRAAISLASGTEE-----SFQNLNCT--GP-PPVIGIVGDPSSTPSIAISSLLRLFRVPIVSYYATCSCLSDRKKYPSFFRTIPSDAFQVRAMVQIL
OlfCq21      98 NLLP-NITLGYHLYDNCVMLGMAFRAAISLASGTEE-----YFSNLNCT--GP-PPVIGVVGDPGSTPSIAISSVLGLFRVPIVSHYATCSCLSDRKKYPSFFRTIPSDAFQVRAMVRIL
OlfCq18      98 KLLP-NITLGYHLYDNCVMLGMAFRAAISLTSGTEE-----SFLNLNCS--GP-SPVIGIVGDPSSTPSIAISSVLGLFRVPIVSHYATCSCLSNRKKYPSFFRTIPSDAFQVRAIVQVL
OlfCq8       98 NLLP-NIILGYHIHDNCVTFGMALRAAMSLVSGIEE-----SFINLNCT--GP-PPIIGIVGHPSSTPSIAISSVLGLFRVPIVSHYATCSCLSDRKKYPSFFRTIPSDAFQVRAMIQLI
OlfCq6       98 SLLP-NISLGYHLYDNCVMLGMALRAAMSLVSGIEE-----SFLNLNCT--GP-PPIIGVVGDPSSTPSIAISSVLGLFRVPIVSHYATCSCLSDRKKYPSFFRTIPSDAFQVRAMIQLI
OlfCq13      97 KLLP-NITLGYHLYDNCVMLGMAFRAAISLVSGTEE-----LFSNLNCT--GS-PPVIGIVGDSNSTPSIAISSVLGLFRVPIVSYFATCSCLSNRKQYPSFFRTIPSDAFQVRAMVNIL
OlfCq14      97 NLLP-NITLGYHLYDNCVRLAMAFRAAMSLASGTEE-----SFSNLNCT--GP-PPVIGIVGDPSSTPSIAISSILGLFHVPIVSHYATCSCLSDKKKYPSFFRTIPSDAFQVQAMVQVL
OlfCq12      98 NLLP-NITLGYYLYDNCVKLSVAFRAAMALVSGTEE-----SFSSLNCT--GP-PPVIAIVGDPGSTHSIAISSILGLFRVPMVSYFATCSCLSDRKTYPSFFRTIPSDAFQVRAMVQIL
OlfCq1      100 NLLP-NITLGYHLYDNCVKLGVAFRSATALVSGTEE-----SFNVLNCT--GP-PPIIGIVGDPGSTHCIAISSVLGLFRIPMVSYYATCSCLSDRSKYPSFFRTIPSDAFQVRAMVLIL
OlfCq10P     99 NLLP-NITLGYQIYDNCLRLGVAFRAATALISGTEE-----TISDLNCK--GP-PPVIGLIGDPGSTHSIAISSVLGLFRLPMISYYATCSCLSDKKKYPSFFRTIPSDTFQVRAMVQTL
OlfCq9       99 NLLP-NITLGYQIYDNCLRLGVAFRAATALISGTEE-----TISDLNCK--GP-PPVIGVIGDPGSTHSIAISSVLGLFRLPMISYYATCSCLSDKKKYPSFFRTIPSDTFQVRAMVQTM
OlfCq5       99 NLLP-NITLGYQIYDTCLRLRVAFQAATALISGTEE-----TISDFNCK--GP-PPVIGLIGDPGSTHSIAISSVLGLFRMPMISYYATCSCLSDKKKYPSFFRTIPSDTFQVRAIVQTL
OlfCq4       99 NLLP-NITLGYQIYDTCLRLRVAFQAATALISGTEE-----TISDFNCK--GP-PPVIGLIGDPGSTHSIAISSVLGLFRMPMISYYATCSCLSDKKKYPSFFRTIPSDTFQVRAIVQTL
OlfCq7P       1 ------------------------------------------------------------------------------------------------------------------------
OlfCq11      98 NLLP-NITLGYQMYDNCLRLGVAFRAATALISGTEE-----TLSDLNCK--GP-PPVIGVIGDPGSTHSIAISSVLGLFRMPMISYYATCSCLSNRKKYPSFFRTIPSDAFQVRAMIQIL
OlfCq3       94 NLLP-NITLGYHLYDNCLKLVVAFRAATTLISGTEE-----TFSNFNCT--GP-PPVIAIVGDPGSTHSIAVSSVLGLFHVPLISYYATCSCLSNRKKYPSFFRTIPSDAFQVRAMVQIL
OlfCq2       96 KLLP-NITLGYQIYDNCLRLGVAFRAAMSLVSGTEE-----S-SNLSCT--GP-PPVIGIVGDPGSTQSITISSVLGLYRVPMVSYYATCSCLSDRKKYPSFFRTIPSDAFQVRAMVQIL
OlfCr1      108 SLLP-GVKLGYHIMDSCSRYPHSLTAAMSMISGG----------NKTCG--PT-KPAKLLIGDSSSTQSILLSTTLVPLKIPMISYLAGCPCLSDRQKYPNFFRTIPSDFYQARTMVQIA
OlfCt1       99 QLLG-NLTLGYKIYDSCSTHFHALRTALTLMNGNEE-----IAGISECR-----GGVPVVIGDGGSTLSIVVAQFLGVFHVPQISYFSSCACLSNKLDFPAFLRTIPSDFFQVDALVQLV
OlfCs2       93 FLLP-NIILGYLVADTCLAESTTLSAALAIMTGQEE-----TVSDTECI--VA-PRVPVIVGDARSSASIGVADTLGVFDIPMVSYFASCACLGDNHRFHTFLRTVPSDAFQAKAMARLL
OlfCs1       98 VLLP-NVTLGYLVADTCLAESTTLSAALALVTGQET-----TVFSTECN--GA-PMVPVIIGDARSSASVVVADTLGVFDIPMVSYFASCACLSDKHRFHTFLRTVPSDAFQAKAMARLL
OlfCv3       94 DLLP-NYTLGYKIFDSCAYPLTGQRSAVAVLNGPN------ELESPLCA--DA-APLLAVIGESGSAQSIVVSRILQPFGIPMISYFSSCACLSDRREFPTFFRVIPSDAYQVKAIAKLL
OlfCv2       90 DLLN-NFTLAYKIFDSCSTPVTAQKAVLAVMNGQE------VVQSSMCS--GA-GPLIGLIGESGSSQSIVLSRTVQAFQIPMISYFSTCSCLSDRKQFPTFFRVVPSDDYQVKAIAQLL
OlfCv1       91 DLLP-EHTLAYRIFDSCATPVMAQKAVLAALNGQD------VVQSFMCS--GA-SPLLGLIGESGSSQSIVVSRTLEPFRIPMISYFSTCSCLSDRKQFPTFFRVVPSDDYQVKAIAQLL
OlfCw1       98 TLLP-NITLGYRLYDTCGLIALSLRTALSVVSQPMK-----RSSSEMCS---S-PSIPIIIGDSGSTLSMAISRLLNLFHIPLVSYFASCACLSNKHQFPYFFRTIPSDVNQANALARLV
OlfCn1       92 ELLP-HISLGYKIMDSCASPTNVLRAVFSLVSEQKE-----QEFTSQCH-----LPLTALVAESGSSQSLAVAGMLGPFRVPMVSYFSTCACLSDRKKYPSFFRTIPSDFYQAKALASLV
OlfCu1      100 SLLP-GVALGYRILDSCDHVHTSLRSALFLLNGTFGQTQTDAVGTANCL--ST-APVPAVIGLASSSPTRAVAQTLGPFGIPLISYFATCTCLTDKKEYPSFLRTVPSDRFQVQGLVQLV
OlfCx3      100 DLLP-NLSLGYHIRDSGDDIPVSVKRSLLLVNGQPE-----KGSGQSCEDIQM-QPSPVIVGDAASGVSMTVLRTLGSFKIPLVSYFASCSCLSNKREFPAFMRTMPSDLFQIKALVKLI
OlfCx2      100 DLLP-NLSLGYHIRDSGDDIPVSVKRSLLLVNGQPD-----KDSGQSCEDTRK-QPSPVVVGEASSGISMAVLRSLGCFKIPLVSYFASCSCLSNKKEFPAFMRTMPSDLFQIKALAKLV
OlfCx1      100 DLLP-NLSLGYHIRDSGDDIPVSVKRSLLLVNGQPE-----KGSGQSCEDTRK-QPSPVIVGEASSGVSMAVLRTLGTFQIPLVSYFASCSCLSNKREFPAFMRTMPSDLFQIKALVKLV
OlfCc1      105 DLLP-KTELGYVIYDSCFTISKAVEGTLTFLTGQDE-----AVPNYRCG--NG-PPLSALVGAGGSDLSIATARILGLYYFPQVSYESSCSVLESRFQYPTFLRTIPSDENQSVAMAKLV
OlfCa1       92 MLSSLNITLGYRIYDTCSDVTTALRAVHDIMRPF-------SDCESPEDSSQPVQPIMAVIGTTSSEISIAVARDLNLQMIPQISYASTATILSDKSRFPAFMRTVPSDEYQTCAMAKLL
OlfCb1P      89 NFLP-GIKLGYKICDPCASPTKALHCLEHLLAIN-------GSLPALLDYSDFCPPSKAIMGERYSELSIAIAKLLSLYLVPQVSTSSSSPVLSDKLRYPSFMRVIPSDVYQAQALVKLM
OlfCy1P       1 ------------------------------------------------------------------------------------------------------------------------
OlfCq15P      1 ------------------------------------------------------------------------------------------------------------------------
```

```
                               *                                                            ++                        *+
OlfCd2     208 KHFGWTWVGTVRSRSDYGNNGITAFEDAAKQEGICIEYSEAILRTDPQ-----EQFRKTVEVI-KKGTSKVVVAFISFGDFAPLVKVIAENNIT-GLQWVGSESWITS-RNLAE-TKEYS
OlfCd3     209 KHFGWTWVGTVRSRSDYGNNGIATFEEAAKKEGICIEYSEAILRTDPQ-----DQFLKTVEVI-KKGTARVVLAFVALRDFYPLLKEIAQQNIT-GLQWVGSESWITS-RTVAE-TKEYS
OlfCd1     208 KHFGWTWVGTVRSRSDYGNNGIATFEEAAKQEGICIEYSEAVFKTDPE-----VQFLKTIEVI-KKATTKVVVAFMAFGDFVLLLKVMAQQNIT-GIQWIGSESWITS-RNLAE-TKEYH
OlfCf1      87 RHFGWTWVGALSNKNDYGIDGITTFIKSATEEGVCIEYIEAFESTSQY-----TEILRIVNII-RSSTSKVIMAFMSHREIKLLVDELYKQNVT-GLQWIGSDAWITD-DSLAD-IYGHT
OlfCe1P    200 KYFGWTWVGALCSDNDYGNNGMNTFIKAATEFGVCVEFSEAFFRTHPR-----EEILRIVDVV-KKSSSKVIVAFVSYSDMEVLLLELAKQNIT-GLQWIGSESWISD-MNIAT-GKWQY
OlfCm1     207 RHFGWTWVGAIRTDDDYGNNGMATFTKVAEQMGICLEYSVPFFRTYAE-----DKVIRIIEQI-KSSTSRVIVAFLVHWDLEVLLHKFVEYNIT-GYQWVGTEGWISD-SVIAT-MDTHH
OlfCm2     207 RHFGWTWVGALRTDDDYGNSGMATFTKVAEQMGICLEYSLPFFRTYTE-----DKVMRIIEQI-KSSTSRVIVGFLAHWDLEVLLHKFVEYNIT-GYQWVGTEAWISD-SVIAS-MDTHH
OlfCg9     204 KHLGWSWVGAVNSDNDYGNYGMAIFLNTAHKEGICVEYSEKFYRTEP------EKLKKVVDTI-KKSTAKVIVAFISFLEMRLLVEQLSAENIT-GLQIVGVEGWTTS-KSLIT-PKTLN
OlfCg7     204 KYLGWSWVGAVNSDNDYGNYGMAIFLETAQKEGICVEYSVKFYRTET------EKLKRVVDTI-KKSTAKVIVAFVSFIEMGLLIEQLSIQNIT-GFQIIGVEGWITS-KNYIT-TNSFH
OlfCg1     204 KHFGWSWVGTVNSDNDYGNNVMAIFLNTAQKEGICVEYSVKFYRTEP------EKLKKVVETI-KKSTAKVIVAFVSLVEMGLLIDQLSIQNIT-GFQVIGGEGWITT-KSLIN-SKSFH
OlfCg5     204 KYFGWSWVGAVNSDNDYGNNGMAIFLKTAQEEGICVEYSVKFYRTEP------EKLQKVVETM-KRSTTKIIIAFLSRVEMVNLLEELSIQNIS-GFQVIGVEAWTTA-KSLIT-PNSFH
OlfCg12    204 KYFGWSWVGAVNSDNDYGNHGMAIFLSTAEKEGVCVEYSVKFQRTEP------EKLKKVVDTI-KKGTSKVIVAFLTEFEMKNLLEYLIIQNVT-GLQVIGVEAWITA-NSMIK-PNSFH
OlfCg11    203 KHFGWSWVGAVNTDNDYGNNGMTTFLNTAQEEGICVEYSVKFYRTEP------EKLQKVVETI-KKGTAKVIVAFLTSSEMYNLLEQLSIQNIT-GLQMIGVEGWITA-KSLIT-PNSFH
OlfCg6     203 KHLDWSWVGAVNSDNDYGNNGMAIFLKAAHEEGICVEYSVKFYRTEP------EKLKKVVDTI-NKGTAKVIVAFVSFVEMGLLIDQLSIQNIT-GIQMIGVEPWITA-NTYIT-SNSFR
OlfCg2     201 KHFGWTWVGAVSSDNDYGNNGMAIFQKIAQEEGICVEYSVKFYRTES------DKLKKVVNEI-KKGTAKVIVAFVSFVEMGLLIDQLSIQNIT-GFQLIGVESWITT-KNYFT-PNSFS
OlfCg10    204 KHFGWSWVGAVNSDNDYGNNGMAIFLNTAQEEGICVEYSEKFYRTDP------EKLRKVVDTI-KNSTAKVIVAFLTSLEMENLLQELTKANIT-GLQIIGVEAWITA-NSLLT-PNSFR
OlfCg4     204 KHLGWTWLGAVSSDNDYGNYGMSIFQKIAQEEGICMEYSVKFYRTEQ------EKLQKVVETM-KKGTAKVIVAFVSFLEMGLLIDQLSVQNIT-GLQMIGVKSWITS-ENYIT-PKSFH
OlfCg3     204 KDLGWSWVGAVSSDNEYGNYGMSIFLKIAQEVGICVEYYAKFYRTEA------ENLHKAVNMI-KKGTAKVIVAFVSLPEMGILVDQLSIENIT-GLQMIGVKSWITV-KSFIT-PSSFH
OlfCg8     204 KHFGWTWVGAVNSDNDYGNNGMATFQKTAKEVQICVEYSVKFIRTET------EKIRNVVNVI-KKGTAKVIVAFLTGFEMKSLLEQLSSQNIT-GLQMIGVEAWITS-KTLIT-SKSFH
OlfCj1     202 TYFGWKWVGAVRSMGDYGNNGMATFLEAAEKEGICVEYSVSIYRTNSR-----EKILEVTDII-KKSTSKVIVAFADGNDLDMLIKELYYQNVT-GYQWVGSEGWITY-RFLAT-AINYA
OlfCh1     202 KHFGWTWIGAVRSDNDYGNNGMATFVEVAEKEGVCIEYSEAISRTNSK-----DKIAKVVEVI-KKGTAKVLMAFLAQGEMDVLLEELIRQNVV-GLQWVGSESWITS-SYLAT-ERTLN
OlfCk3     213 KRFGWTWIGAVRSDSDYGNNGMASFLKAAEEEGICVEYSEAYYRTQPR-----SKLMRVADVI-RRSTARVIVAFMASGDMRLLLEELSQQPLP-PMQWIGSEAWFTD-PEMLR----FN
OlfCk1     213 KRFGWTWIGAVRSDSDYGNNGMASFLKAAEVEGICVEYSEAYYRTQTR-----NKLQRVADVI-RRSTARVIVAFMAAGDMRFLLEELSQQPPP-PMQWIGSEAWVTD-PQMLR----FN
OlfCk2     213 KRFGWTWIGAVRSDSDYGNNGMASFLKAAEKEGICVEYSEAYYRTYPL-----SKLKRVADVI-RRSTARVIVAFVAAGDMRFLLEELSKEPLP-PMQWIGSEAWVSD-PQMLR----FN
OlfCq19    209 KYFRWTWVGLIYSDDDYGVYAAQSFQQEMQLFGGCVAFSEVLPHDNNL-----EDIKHITKMI-QASTARVIVVFSTPSFLIPLIDELVLQNMT-DRQWIASEAWATS-FVHHS-PRLLP
OlfCq17    209 KHFKWTWVGLLYSGDDYGVYAAQMFHKEMQLSGHCVAFSEILPNDHNP-----KDIQRIIRVI-QGSTARVVVVFALSSFLIPLMDEVVLQNMT-GCQWIASEAWATS-LEYHT-PRFLP
OlfCq16    207 RYFGWTWVGLIYSDDDYGIYAAQSFQQEMLLFGYCVAFSAILPHDNNH-----RDIQRITAII-QASTARVVVVFSTSSFLIPLMEEVVVQNMT-GRQWIASEAWTTS-PVYHT-PRFLP
OlfCq20    210 RHFGWTWVGLIYSDDDYGVYAAQSFHLEMQMFGHCVAFSEILPHNNNQ-----RDIQRIMGVI-QASTARVVVVFSTSSFLLPLIDEVASQNMT-GRQWIASEAWATS-PVYHS-PRLLP
OlfCq21    209 SHFGWTWVGLIYSDDDYGIYASVSFQQEMQQFGSCISFSEILPHDNNP-----KDIQRIMEVI-KASTASVVVVFSTSSYLMPLIDEVVLQNLT-GRQWIASEAWATS-PVFHT-PRYLP
OlfCq18    209 KHFKWTWVGLLYSDDDYGVYAAQSFQQEMRQFGLCAAFSEFLPHDNNP-----RTIQHIMGVI-QGSTAKAVVVFAPSSFLIPLMNEVVLQNMT-GRQWIASEAWATS-LESHI-PSFQP
OlfCq8     209 SHFGWTWVGLLYSNDDYGTYAAQSFHQEMQLFGICIAFSEPLRYDSNP-----RDIQRIMAVI-QASTSRVVVVFSPSTLVIPLINEVVLQNMT-GRQWIASESWATS-PVFYT-PHFLP
OlfCq6     209 SHFGWTWIGLLYSDDDYGTYAAQSFHQEMQLFGFCIAFSEPLRYDSNP-----RDIQRLMEVI-QASTSTVVVVFSPSTLVIPLMNEVVLQNMT-GRQWIASESWATS-PVFYT-PRFLP
OlfCq13    208 KHFGWTWVGLIYSDDDYGNYAAQSFLQDIQIYGGCVAFTEILPLNNNR-----KYIEHVVGVI-QASTARVVVVFSTSTYVLPLMDEVVLQNVT-NRQWIASEAWATS-PMFHT-QRLLP
OlfCq14    208 RHFGWTWVGLLYSDDDYGTYAAQSFQKEVQLFGGCIAFSTILPQ-NNP-----REIQNIMGVI-RASTARVIVAISTSSYLLPLMDEVVLQNLT-GRQWIASEAWATT-PAFRT-PRFLT
OlfCq12    209 KRFGWTWVGLLYSDDDYGIYAAQSFQKEMQLFGACVGFSEMLPRDNNH-----EDIQSIVEVI-QTSTARVVVVFSTEAYLLPLMDEVALKNVT-GRQWIASEAWATS-PVFHT-QHLLP
OlfCq1     211 KHFGWTWVGLLYSDDDYGINAAQSFQKEVQLFGGCVSFSEILPLDNNH-----MDIQRIVQVI-QASTAIVVVVFSTEAYLLSLMDEVVLQNVT-GRQWIASEAWATS-SVFHT-KRLLP
OlfCq10P   210 RHFGWTWVGLIYSNNDYGIYAAQSFHQEMQLFGHCVAFSEILPQDNNP-----RVIDHIMGVI-QASTARVVVVFSASSVLIPLMNEVVLQNLT-GRQWIASEAWVTS-AVFRT-PYFQP
OlfCq9     210 RHFGWTWVGLIYSNNDYGIYAAQSFHKEMQLFGHCVAFSEILPQDNNP-----RVIDHIIGVI-QASTARVVVVFSASSLLIPLMNKVVLQNLT-GRQWIASEAWVTS-TVFRT-PYYQP
OlfCq5     210 RHFGWTWVGLIYSNNDYGIYAAQSFHQEMQLFGHCVAFSEILPQDNNP-----RVIDHIMGVI-QASTARVVVVFSASSLLIPLMNKVVLQNLT-SRQWIASEAWVTA-AVFRT-PYFQP
OlfCq4     210 RHFGWTWVGLIYSNNDYGIYAAQSFHQEMQLFGHCVAFSEILPQDNNP-----RVIDHIMGVI-QASTARVVVVFSASSLLIPLMNKVVLHNLT-SRQWIASEAWVTA-AVFRT-PYYQP
OlfCq7P      1 ------------------------------------------------------------------------------------------------------------------------
OlfCq11    209 RHFEWTWVGLLYSDDDYGINAALSFHHDVQQFGGCVAFSEILPNDNNQ-----MAVQHIVRVI-QNSTAKVVVVFSTSSYLLPVIDEMLLKNIT-GRQWIASEAWSTS-PVLLN-PRLRY
OlfCq3     205 KYFGWTWVGVLYSDDDYGIYAAQSFQQEMQRFKGCVAFSEIVPYDNH------RDIQRIVAVI-KASTARVVVAFSTD--LLPLMEELLQQNVT-GRQWIASEAWSTS-PVLHL-PRFVP
OlfCq2     206 RHFGWTWVGLLYSNDDYGIYAAQSFQQEMQILGGCVAFSEMLPYDNNG-----RDIQRIAGVI-QKSTAKVVVAFSTD--LASLMDELLLQNVT-GKQWIASEAWTTT-PVLQT-PQYLP
OlfCr1     214 KRFGWTWIGAVIADSDNGHGTLQALEEEIKGTGICLAFYHTLYRERLQ-----KDVAFAARTV-QASSARVILVLAWYTDVEAFLLELMRRNVT-DRLFLASEIWSTSYNIMAN-PQLYT
OlfCt1     208 QHFGWSWIGTFAGDDAYGRGGAQIFNEKVTKLGACIAFYEIIPKNHQQ-----TEMSRIVERI-LESRSHVILVFALEQDARALFLEVLHHNLT-GIQWLASEAWITA-AILST-PEFHS
OlfCs2     204 HLLDWTWVGVVAGDDEYGKSGVQLLLKELEHVGVCVDYLEFIPKSHSQ-----RRIRRTVETI-QSSTAHVVVTFAIAPDIEVLFKEVVVQNVT-NRQWIATEAWSTS-VQFLD-PASIP
OlfCs1     209 HLLDWNWVGVVAGDDEYGKSGSHLLLKELEGSGVCVDYVEVIPKSHSQ-----SRIKQIMERI-QSSTASVVVTFAIGPDLEVIFKEMVVQNVT-NRQWIATEAWSTT-VQYSD-PESIP
OlfCv3     204 RHFNWTWIGVIRGDHEYGRFALQGLLKELEGTGICVAYQKMIPLLYDR-----QKALEIIHVM-SHSTARVVVVFSAEGELTPFLRDYMEQNVT-GIQWIASEAWVTS-SVFAG-SEFDP
OlfCv2     200 KRFDWTWIGVVTEDHDYGRFALQGLKREIENTNICLAYHEMIPKDYTQ-----ERVLKILKVM-KESTAKVVVVFSVEGEFYPVLREFVAQNIT-GIQWIASEAWVTA-SMLAE---TYS
OlfCv1     201 KRFDWTWIGVVTEDHDYGRFALQGLKREIENTKICLAYHEMIPKDYTQ-----ERVLKILKVM-KESTAKVVVVFSGEGEFYPFLTEFVAQNIT-GIQWIASEAWVTA-SMLAE---TYS
OlfCw1     208 KHFGWTWVGTVGADDAYGRTGIDLFTAAVTQLGVCVSYRIIIPKLPTQ-----QQLQDIVRTI-RDSSAHVLVAFAIEEDIKPVVDEIVLQNVT-GKQWVASEAWVTS-TLIST-KENFP
OlfCn1     201 KQFGWTWIGALQSDNDYGRNGISAFTKEVEKMGVCIAFVGTILRTYPQ-----SKITEVVEMI-KESTVKVILAFVPEGDLYPLMKEVVNRNIT-GIQWIASEAWVTA-ARPST-PEMFK
OlfCu1     216 SHFGWRWVGTVGTDDDYSHYGIQAFTEQLERLGSCIAFHQTIPKSPSQ-----AQIRSILNSL-ESSTAQVIIAFATEGELLELLVEVARRNLT-RLQWVASEAWVTA-KLLTI-PELHP
OlfCx3     213 NYFQWTWVGLIGVNSDYAHFAIQLFLKESEQFNICPAYVHFYPVVLME-----DALEELVNIL-KASSATVIINFSGESELHSILKECRRQNVT-HLQWIASEAWATS-KSLWE--EFGD
OlfCx2     213 HYFQWTWVGVIGVDTDYARFAIQLFLKESQQLNICLAYVQLYPVALNQ-----DSAEELVRML-KSSSATVIISFSVDSYLYGILKECRRQNVT-HLQWIASEAWATS-KSLWE--EFGD
OlfCx1     213 YYFKWTWLGVIGVDTDYARFAIQLFLKESEKYNICPAYVHIYPVALTQ-----NTVEELVKIL-KSSSARVILNFSVDSYLYGVLKECRRQNVT-NLQWIASEAWATS-KVLWD--DFGD
OlfCc1     216 LRFGWTWVGTIAAEDDYGKYGIKRFKEVVEEAGVCISFSETLPKISNP-----EAIQRIVQTV-HDSTAKIIVVFSSDVDLSPLVEALLQSNVT-NRTWIASEAWVTS-AAISRQPNVLS
OlfCa1     205 KSNKWSWVGIIITDGDYGRSALEGFIQHTETEGICIAFKAILPDSLADQQKLNTDIENTLNIIENNPKVRVVISFAKSSQMQLLFKGLQSRNISNNMVWVASDNWSTA-KHILN-DGSIT
OlfCb1P    201 SHFSWNWVGVVYGDDDYGRGAYQSFMEESQGK-ICADFEKVVPHYLDQ-VDVDKYIQEAANAI-RSSSANVTLLILKPQLVEKLFKEMIKTNTS--RVWIASDAWSMF-RPLTQ-MKDIN
OlfCy1P      1 ------------------------------------------------------------------------------------------------------------------------
OlfCq15P     1 ------------------------------------------------------------------------------------------------------------------------
```

```
                                                                                                             ++
OlfCd2     319 FLSGAVGFAIVNAKLVGLKEFLVNVH-PDQEPK-NKLLKEFWETAFQCSFS-NSD------------S----ALCTGSEKLGELKNEYTDVSELRIEHKAYAAVYAVAHTLHNVFKDVK-
OlfCd3     320 FLSGAVGFSIANVKLVGLLDFLVNVH-PDHEPK-NKLLKEFWETTFQCSFN-NRG------------S----VGCTGSEKLANLQNEYTDASELRIANKVYTAVYAVAHTLHNIFQDFK-
OlfCd1     319 FLSGAVGFAIANSKPVGLREFLMNVH-PDKELN-NELLKEFWETVFLCSFR-NSS------------S----GGCTGSEKLSELQNEYTDFSELRIENKVYTAVYAVAHSLHNVLKGFKF
OlfCf1     198 LLTGSIGFAVRNAQIPGLGSFLQKVN-PSQFPN-SIFVKDFWEHVFDCSFT-----------TSKL-T----KRCNGSENLSDVQNSFTDLTDLRFSNNIYKAVYAVAHAINNLLLCEEN
OlfCe1P    311 ILRGSMGFAIPKAEIQGLREFLIKIH-PSSN---IYLYKELWESVFQCRLS-TEQ-------SSES-K----SMCTGNESLNNVQNQYTDVTELQIANNVYKAVFAIAHALNSSVGCSKW
OlfCm1     318 ILQGAVGLAIPKTEVTGLKEYILNIK-QLKSSG-STIFSELWESLYQCKYP-NKD-------DSVS-M----NACTGNEELSQMQNSFTDMSLMPIFSNVYKGVYAVAHTLHELLGCKE-
OlfCm2     318 ILQGAVGLAIPKTKVTGLQEFILNIT-PLKSSG-GAIFSEFWEALFQCKYS-NKD-------TSVS-I----NACTGKEELSQVENLFTDMSLMPIFSNVYKGVYAVAHALHELLGCKD-
OlfCg9     314 VLRGSLGFAMRKIYIEGFAEYV---------------LKPFWDTAFPCIPN-QRN-------DSWV-I----LNCSRYQDLLVLKNYIEDVPEHRFSINVYNAVYAVAHALHSLFKCKE-
OlfCg7     314 SMGGSLGLALRKIHLEGFLDYV---------------TKSFWSTAFPCSQT-EGI-------LP---T----VGCSKYKDLLPLKNYTEDVPEHRYSSHVYKAIYAVAHSLHSLLKCKE-
OlfCg1     314 VLGGSLGFAFRKIIIEGFADYV---------------IKTFWERDFPCLTN-EGN-------YSQY-A----LTCSSYQDLFTLKHYNEDIPEQRYASNVYKAVYAVAHSLHNLLKCKE-
OlfCg5     314 ILGGSLGFAVRKIDTEGFLDYV---------------IKSFWDSAFPCLIV---N-------SSQL-K----VNCSSYKDLLVLKNDNEDVPEQRYASNVYKAVYAVAHSLHSLLNCKE-
OlfCg12    314 VLGGSLGFAVKKLNIEGFEDYV---------------TKAFWETAFPCSQTSQKE-------NSQY-K----LICNIYRDLLVLKNDNKDVPEQRYASNVYKAVYAVAHSLHNILKCRE-
OlfCg11    313 VLGGSLGFAVRKTAIEGFADYV---------------IKSFWETAFPCTMT-IGN-------SSQY-S----LSCGIYQDLLLLKNYNEDVPEQRYSTNVYKAVYAVAHSLHSLLKCKE-
OlfCg6     313 AMGGSLGFATKYIYIEGFAEYV---------------MTPFWNTAFPCSES-DRN-------HSHY-E----LICSRYEDLLALKNDNKDVNEHRYSSNVYKAVYAVAHSLHGLLNCKE-
OlfCg2     311 SLRGSLGFAVRKINIEGFIDYV---------------IKAFWDTAFPCTKT-EGN-------SSQY-S----ISCTRSEDLLELKNYTEDVLEQRYASNVYKAVYAVAHSLHSLLGCEN-
OlfCg10    314 VLGGSLGFAVPKVNIQGFSNYV---------------IKDFWETAFPCSET-EIN-------VSQYSS----LSCNSYDDLLLLKNYNEDVPEQRYASNVYKAVYAVAHALHSLLKCKV-
OlfCg4     314 VLGGSLGFAMRKMYIEGFSDFA---------------LKTFWEKHFQCSQT-KPN-------ASNY-A----SSCSRYNDLLMLKNYKEDVTEHRYSSNVYEAVYAVAYSLHSLLKCKE-
OlfCg3     314 VLKGSLGIAVKKISVEGFAEYA---------------INEFWKKGFPCLKS-EVN-------SSRY-A----LSCSRYEDLFVLKSISEDVPEQRYASYVYKAVYAVAHSLHSLFNCKE-
OlfCg8     314 VLGGSLGFAVRKIQIEGFADYV---------------MKAFWDTAFQSF--------------SNA-D----LNYSQYQDLLLVKNYNEDVLEQRFVSYVYKAVYALAHSLHSLLRCTE-
OlfCj1     313 VVGGAIGFAVPNAYIPGLKEFITGSQ-PSLRPG-NTGLVELWESVFDCTLN-SQT--------HNA-S----KICNGQESLANINTRFTDVSDASLLNNVYNAVYAVAHAVEELLTCEKG
OlfCh1     313 ILGGAIGFTIIKSKIPGLKEFLLKVG-PSQNLS-NALLGEFWEMVFGCCLS-PTVCP-----NSEH-A----TFCDGSENLTNVSNAFTDVSELRISNNVYKAVYAIAYALHNTITCKNS
OlfCk3     321 MCIGGVGFAIPRSVIPGFRTFLLDLS-PQRAMK-FPLLTEFWESSFSCSLK-QQTG------PSTG-M----PACDGTEDLGRLKNPYTDTSQLRISNMVYKATYAIAHALHGII-CN--
OlfCk1     321 LSIGAVGFAIPRSVIPGFRKFLLDLS-SEQALK-IPVLKEFWESSFGCSLK-QHTG------YFSG-M----PACDGTEDLGTLKNPYTDTSQLRISNMVYKATYAIAHALHGIV-CN--
OlfCk2     321 LSIGTMGVAVPRSLIPGFRKFLLDLS-PYKVLK-FPLLTEFWESSFSCSLQ-QQTD------PSTG-M----PACDGTEDLGRLKNPYTDTSQLRVSNMVYKATYAIAHALHGIV-CN--
OlfCq19    320 FLKGTLGIAIRRGEIQGLYEFLLRLQ-PTNDPK-NNMIRIFWEIMFGCNFE-TGGKKNS---GDQV-I----NICTGQEDLRTTKTPYTEVSELRASYNVYKAVYALAHALNDLMQCEDG
OlfCq17    320 FLGGTLGIAIRRGEIEGLHEFLLRIR-PSNDTS-QNIVRIFWENLFGCRFE-TGGLKTG---REQE-K----MECTGQEDLSTTNTPYTDVSGLRASYNVYKAVYALAHGLHDLMQCEEG
OlfCq16    318 ILGGTLGIAIRRGEIEGLHDFLLRLI-PSNDKK-NSIIRIFWENIFGCSFE-KWGTETF---GEQV-K----NICTGQEDLSTTDTPYTDVSGLRAAYNVYKAVYALAHGLHDLMQCEEG
OlfCq20    321 FLGGTLGIAVRHGEIPGLHDFLLHLG-PGNESR-NNMLRIFWENMFGCSFE-PGVKD-----TNSE-I----KLCTGQEDLSTTNTPYTDVSGLRAAYNVYKAVYALAHGLHDLMQCEKG
OlfCq21    320 FLGGTLGIAIRRGEILGLQEFLLHIH-ASNNPK-DNMLKIFWENMFRCSFE-TRSKDTI---GEQE-K----SICTGHEDLSTTKTPYTDVSGLRASYNVYKAVYALAHALHDLMECEEG
OlfCq18    320 FLRGTIGIAIRRGEIQRLHDFLLRIR-PSNDPK-NYMLRIFWENMFGCSFG-KGDTE-----GEQV-I----KVCTGQEDLSTTNTPYTDVSGLRAAYNVYKAVYALAHALHDLMECEEG
OlfCq8     320 FLGGTLGIAIRRGEIEGLREFLLQLR-PQNDPR-NNMPKIFWENMFGCSFE-T---------GAQA-K----NVCTGQEDLSTTNTSYTDVSELRAANNVYKAVYALAHALHDLMKCEEG
OlfCq6     320 FLGGTLGIAIRRGEIEGLREFLLQLR-PKNDPR-NNMLKIFWENMFGCSFE-T---------GPHV-K----NVCTGQEDLSTTNTPYTDVSELRAANNVYKAVYALAHALHDLMKCEEG
OlfCq13    319 FLGGTLGIAIRRGEIQGLHEFLLHLR-PKNDQH-NNMVRIFWEKMFGCRFN-P---------GGKG-D----KQCSGQEDLSSIDTAYTDVKELRASYNVYKTVYALAHALHDLTECDEE
OlfCq14    318 ILRGTIGIAIRRGEIQGLLEYLLRLR-PSSDPR-NNLVRIFWENMFGCSFA-S---QTV---GEQV-K----KLCTGQEDLSITNTAYTDISGLRGPYNVYKAVYALAHALHGLMQCEEG
OlfCq12    320 FLGGTLGIAIRRGEIQGLKEFLYYLH-PDSNTR-NNIVRIFWENIFGCSFE-I---------GGRE-R----KVCTGQEDLKSTNTAYTDVSGLRASYNAYKAVYALAHALHDLMQCEDG
OlfCq1     322 FLGGTLGIAIRRGEIQGLRDFLLSLH-PDSNLR-NNMVKIFWENMFECSFD-TV--------GRKG-E----TMCTGQEDIRTTNTPYSDVSELRASYNVYKAVYALAHALHDLIQCEEG
OlfCq10P   321 FLKGTLGIAIRRGEIQGLHSFLLRLH-PKSDQR-NNIVRIFWETMFGCSFE-TGDEVTF---VQQM-K----KVCTGQEDLSITNTPYTDVSGLRAPYNVYKAVYALAHALHDLMQCKEK
OlfCq9     321 FLKGTLGIAIRRGEIQGLHSFLLRLH-PNSDQR-NNIVRIFWETMFGCSFE-TEDKETF---GQQI-K----KVCTGQEDLSITNTPYTDVSGLRAPYNVYKAVYALAHALHDLMQCKEK
OlfCq5     321 FLKGTLGIAIRRGEIQGLHSFLLRLH-PNSDQR-NNIVRIFWETMFGCSFE-TGDKETF---GQQM-K----KVCTGLEDLSTTNTPYTDVSGLRATYNVYKAVYALAHALHDLMQCKEK
OlfCq4     321 FLKGTLGIAIRRGEIQGLHSFLLRLH-PNSDQR-NNIVRIFWETMFGCSFE-TGDKETF---GQQM-K----KVCTGLEDLSTANTPYTDVSGLRATYNVYKAVYALAHALHDLMQCKEK
OlfCq7P      1 ------------------------------------------------------------------------------------------------------------------------
OlfCq11    320 VLGGTLGIAIRRGEIEGLDNFLLRLR-PDNFSQ-NSMMRIFWENMFECNFD-TI--------GGLR-T----KLCSGQEDLRSKFTPYTDVSELRASYNVYKAVYALAHALHDLMHCEEG
OlfCq3     313 LVRGTLGIAIHRGKIKGLHEFLLHIQ-PDNDPT-NNMVRIFWENMFGCSFE-KG--------NGYG-E----KMCTAQEDLNITVNEYNDVSELRASYNVYKAVYALAHALHDLMQCEEG
OlfCq2     315 LLGGTLGIAIRRGQIQGLYEFLKNLR-PDKNPK-NSTIRIFWETMFGCKFE-VGGKKVDEQ-QERG-R----NKCSGQEDLNNSETAYTDVSELRASYNVYKAVYALAHALHDLVKCEEG
OlfCr1     326 ISKGTLGVALRSAPIPGFSAHLQQLH-PSHYPE-DKFLRTLWENTFGCSPT-ISSSKI----IQNS-L----PPCSGRESLEG-HIEFTDTSHLTVSYNIYLAVYAAAHALHSLLECNSQ
OlfCt1     319 VLQGSLGYAIRRADIPGLQPFLLRLH-PSKYPQ-DPFVEQFWEEMFKCSLG-IINR------SSSI-R----PPCDGSEVLVNINNIYSDVSQLRISYNVYKAVYAIAHALDLMLHCEPG
OlfCs2     315 LLAGTLGFALHRADIEGLGAYLSQLN-PAKQSN-EPFVKDVWEEIFGCSLA-QDWQ------PSFK-R----PKCTGSENVEIHGGIYTDVSQLRVTYNVYKAVYAIAYAIHNMIACQPG
OlfCs1     320 LLAGTIGFALRRAEIKGLGAFLTQLN-PVKQSN-EPFVKDVWEEIFRCSLA-HDRQ------PSFK-R----PKCTGSESVEKHGRIYTDVSQLRVTYNVYKAVYAIAYALHNMIACLPG
OlfCv3     315 FLGGTIGFGIRQGQIPRLKEYLTTVN-PERYPT-NPLVYELWGALYGCSPS-WSN-------LSSH-L----PSCTGKETVRLQYSAYLNTSSPRISYNVYKAAYAIAYSLHNLIYCTPG
OlfCv2     309 FLDGTIGFAIRQGHVPGLQDYIRTVT-PEKYPS-IPQVQELWEALYGCSPS-TST-------LSSH-L----PSCTGKETLRKEYSAYMNTSSPRVTYNVYKAVYAFAHSLHNLIECRNG
OlfCv1     310 FLDGTIGFAIRQGHVPGLQDYIRTVT-PERYPS-IPQVQELWEALYGCSPS-TST-------LSSH-L----PSCTGKEILRKEYSAYMNTSSPRVTYNVYKAVYAFAHSLHNLIECRNG
OlfCw1     319 SLSGTIGFAIRRAEIPGLKHFLDSIQ-PLADPY-NVFAREFWETQFQCTLN-TSLPTSSTMDPVNY-S----HSCTGMERMQDTQSIFNDVSQLRVTYNMYKAVYTVAHALHNLLLCQRE
OlfCn1     312 SFGGTVGFVVRKMAMLKLRPYLENIS-PYSPTQ-SAFVSDFWETVVGCKPC-LNCEPSAN--STLN-G----QMCTGQEKLTFTDK-FFDVTQVRVTYNVYQAVYAIAHAIHKVLYCQGD
OlfCu1     327 VLIGTVGFSFRGTAIPGLAEFLFRVR-PSSRPE-SAFTNMFWEELFGCRLG-YEDS------NDSV-L----PLCTGSENLLETESSYTDVSRVRISYNVYKAVYAIAHALHQLLQCGSK
OlfCx3     323 LLKGTLGFAIHRADIPHLGSYLKEIR-PQ---T-SHFLSEFWEETFRCRLN-GSLNTHAHGEQAQN-W----PACNGSENLDNVYTTYSDVSQLRVSYNVYKAVYLIAHALHDMSTCVPG
OlfCx2     323 LLKGTLGFAIHRADIPHLGSYLKEIR-PQ---T-SHFLSEFWEETFRCRLN-GSLNTHAHGEQAQN-W----PACNGSESLDDVYTTYSDVSQLRVSYNVYKAVYLIAHALHDMSTCVPG
OlfCx1     323 LLKGTLGFAIRRADIPHLGSYLRSVS-SSVAQT-SPFFTEFWEETFHCRLN-GSLNTHVHEEASYN-W----PACNGNETLDDVYTLYSDVSQLRVSYNVYKAVYLIAHALHDMSTCDPG
OlfCc1     328 LLGGTIGFAVKRAEIPGLKKHLLSIS-PF---N-DSLTEEFWGIVFNCTTN-YTL-------ILKG-M----RRCTGEEMLGTVDNTYSDVSQLRITYNVYKAVYAVAHALHNLEQCKTG
OlfCa1     323 DIGKVLGFTFKSGNFTSFHQYLKNLQFESEDEMNNSFLKEFLKL--------------------------------NAGNASNTVLELMKSTNLDKIFSIEMAVTAVANAVAKLCA----
OlfCb1P    314 KVGPIFGFSFSLGNIPGFEDYLRNLR-PTPGGK-NDFIEEYQQLRLNCSLW-P-------------------SNCTTDD--------VLYAVELREAYRERVAIYAIAHGLRELLKCN--
OlfCy1P      1 ------------------------------------------------------------------------------------------------------------------------
OlfCq15P     1 ---GA-HIAIRRGEILGLQEFLLYSH-ASINPK-NNMLKIF-ENMFRCSFE-TRG-------KDTI-GEQEKSICTGHENLSTTKTPYTDVLGLRASYNVYKTVYALAHALHDLMEFEEG
```

```
OlfCd2    419 -------APNNSKRELPTPQKVLQYMRNLSFTIK-TGENIFFDASGDPVARYDLVNWQTAEDGS-LQFKHVGIYDSSLPSKQRLQVNQE-IMLWAGK-SGLLPLSVCTESCLPGTRKAVQ
OlfCd3    420 -------SANRSKLKQPTPQMVLNYMKDVRFTVK-TGEEIFFDTSGDPVARYDLVNWQPSGDGS-LQFKNVGIYDSSLPSEKCLQVNQE-HVLWADN-SRQLPVSVCSESCPLGTRKAVQ
OlfCd1    420 -------FTNSSKKQLPTPQKVLEYLRDVNFTVN-TNENIFFDASGDPVARYDLVNWQPTKDGS-LQFKLVGIYDSSLPSEQRLQINQE-SMLWAGN-SGQLPVSVCSESCPPGTRKAVQ
OlfCf1    300 KSSS---PNACLKLAEIKPWQLLNSLQSVNFTTP-GGESVFFDSNGDSPARYELINLQSVDRGK-IDVETIGYYDASLPKDQRFSMNNV-KVVWREG-GDQVPVSVCSTSCPSGTRKAMQ
OlfCe1P   414 DTN----QRECNT---WQNLQVLQALREVSFFTE-TGEKVFFDKNGDPAARYDLLNWQQGEEGA-TKFVKVGFYDASLQPEFQLSFNNI-TIMWA-----KVPVSVCSESCPMGTRKAVK
OlfCm1    422 ---------KCASKKQLDPITFLKHLRKVRFKTK-DGEDVYFDENGEAVARYEVINWHPSNGKH-DQFVTVGLYDASLPVKDRLAVNVA-SIVWSNN-ATKVPVSVCSESCSPGTRKAVK
OlfCm2    422 ---------KCALKKQPDPVTFLKHIRKVHFKTK-DGEEVYFDENGDPVAKYDIINWQQSTKQH-YEFVTVGFYDASFMGMDRLAVNMS-SIFWAIN-STKVPVSVCSESCPHGTRKAVK
OlfCg9    405 -------QEGCEKDLLTQPQQVVDALKKVNFTVK-MGDRVWFDSTGATLAQYEVVNWQQNSDGS-VNFKKVGYYDASLPPDQRFVLNIK-EILWAGG-NLKKPRSVCSESCPPGTRKAAQ
OlfCg7    403 -------GEDCEKGHATQPQQVVEALKKVNFTVK-FGDRVWFDRTGATVAHYEVVNWQQDTDGS-FQFKQVGYYDASLPPDQRFVLNIE-SIIWPGG-NLEKPRSVCSESCSPGTRKAAQ
OlfCg1    405 -------NEGCEKSLTMQPQQVVEALKNVNFTLK-FGDRVWFDSTGGAVAHYEVVNWQQDSDGS-FQFKKVGYFDASLPPDQSFMLNTK-NIIWSGG-QLERPRSVCSESCPPGTRKASQ
OlfCg5    403 -------KKGCQKNLTIQPYQMVEALKKVNFTIK-FGDRVWFDSTGATIAQYEVVNWQQDSDGL-IQFKPVGYYDASLPPDKRFVVKTE-NIIWAGG-KLKKPKSVCSESCPPGTRKAAQ
OlfCg12   406 -------NGACETDMKIQPQQVVEALRKVNFTIK-MGDCVWFDSTGAVVAQYEVVNWQPDYNGS-IQFKPVGYYDASLPPNQRFVINTE-NIIWAGG-QLKKPRSVCSENCPPGTRKAVQ
OlfCg11   404 --------DGCKKGLAIQPQQVVGALKKINFTLK-LGDIVSFDSTGATVAQYEVVNWQKDANES-IKFKPIGYYDASLPPHQRFVLHTE-NIIWAGG-QLDRPRSVCSESCPPGSRKAAQ
OlfCg6    404 -------QEGCEKGLTIQPQQVVKALKNINFTIK-SGDSVWFDNTGSVVALYEVVNWQKDSDGS-FQFKSVGFYDAMMPPYKNLRLNTK-NIVWAGG-QLEKPRSVCSESCAPGTRKAAQ
OlfCg2    402 -------QGLCDKNKPIRPQQMVEALQKVNFTIQ-MGDQVWFDSTGGTVAHYEVVNWQQDFDGS-FQFKTVGYYDASLPPQQRLMLNTK-NIIWAGG-QQKKPNSVCSESCPPGTRKAAQ
OlfCg10   406 -------NEGCKKDLKIQPQQVVDTLKEINFTIN-MGDRVWFDSTGATIAQYEVINWQQGSDGS-IQFKTVGYFDASLPHDQRFVLNTE-SIIWTGG-QLEKPKSVCSENCPAGTRKAVQ
OlfCg4    405 -------RVKCEKGMTIQPQQVVEALKKVNFSVK-FGDRVWFDSTGSTVAQYEVVNWQRISDES-FQFKTVGYYDASLPPNQRFLLNTE-NIIWAGG-MLEKPRSVCSESCPPGTRKASQ
OlfCg3    405 -------HEGCEKDLTIQPQQVVEALKKVKFTIK-TGDHVWFDSTGAAVAQYDVVNLQLDSYGS-TLFKSVGYYDASLPPEQQFVLNTT-NIIWAGG-E-QKVRSVCSESCPPGTRKATQ
OlfCg8    399 -------QGGCGKALTIQPHQLVEALRKVNFTVK-MGDQVWFDSTGGVIAQYDVVNWQQNSDGS-VQFQSVGYYDASLSPDQRFMLNTE-KIVWAGG-QLEKPRSVCSESCPPGTRKASQ
OlfCj1    417 KGPFH--KKTCAEKGKIQPWQVLYYLTQVNFTTK-NGENVHFDKHGDPVARYTLVNWQMSYEGI-ITFESIGLYDASKPEGQEIQMRDDIEAIWAGN-QKKVPLSVCSETCLPGTRQAFV
OlfCh1    420 NGGNE--NITCGDVDLLVSSQVLHSLQNVNFTMD-SGETVYFDKNGDPMAKYELVNWQKNGAGE-TKFITVGQYDASLSSEQQFVINSF-DIIWAGD-SPTKPISVCTESCQPGFRQAVI
OlfCk3    424 -------EKKCNKNIKIEPREVLDKLKQVKFSK--NNYSVSFDAYGDPVAVYELVNWQLQKDGS-IDFVTVGKYDSSQPKGKEFSLSR--AIIWYDG-TEEVPVSVCSESCPPGTRKAVQ
OlfCk1    424 -------GKLCDKNIKVEPRKVSDQLKQVNFSK--NNYSVSFDANGDPVAVYELVNWQLQGDGS-IDFVTVGKYDASQPKGQEFSLNR--AIIWYDG-TEKVPVSVCSESCPPGTRKAVK
OlfCk2    424 -------ETQCSKNIKVKPRQVFDQLKQVNFSK--NNYSVSFDANGDPVAVYELVNWQLQGDGS-IDFVTVGKYDASQPEGQEFSLNK--AIIWYDG-SEKVPVSVCSENCPPGTRKAVK
OlfCq19   429 RGPFS--VNSCADITNLKPWQLVHYLQNVNFTTD-FGDHVSFDKNGDALAIYDVLNWQPSSDGS-IRAQTVGVVNEEVATGMVLTLDED-ALYWNFE-SKKPPSSVCSESCAPGTRLATI
OlfCq17   429 RGPFI--GNSCADITSLKPWQLVHYLQNVNFTTG-FGDHVTFDKNGDALAIYDVLNWQPSSDGS-ITVHKIGVVYEGATTGMMLTLDAD-AIYWNFE-TKKPPLSVCSESCPPGTRQATR
OlfCq16   427 RGPFN--GNSCAETTNLKPWQLVHYLQNVNFTTG-FGDQVSFDKNGDALPIYDVLNWHPSTDGS-IKLHTVGLVNKGAAMEMVLTLDDD-AIYWNFE-TRKPPQSVCSESCPPGTRQARR
OlfCq20   428 RGPFR--GESCADITNLKPWQLVHYLQKVNFTTG-FGDHVSFDENGDALAIYDVLNWQPSSDGS-IQIFTVGVVKERTETGMVLTIDED-AIYWNFE-TKKSPQSVCSESCSPGTRRATR
OlfCq21   429 RGPFD--GNSCGNITNLKPWQLVHYLQKVNFKTG-FGDHVSFDENGDALAIYDVLNWHPSSDGS-IRLHTVGVVNEGAGTGKVLSLDES-ALYWNFQ-TKNPPRSVCSASCPPGTRQAMR
OlfCq18   427 KGPFS--KNSCADKTNLKPWQVVHYLQNVNFTTG-FGDYVSFDKNGDALAIYDVLNWQPSSDES-IRIYTVGVVKEGTETGMVLTLDED-AIFWNFE-TRKPPRSVCSESCPPGTRRATR
OlfCq8    423 KGPFS--GNSCADITNLKPWQLVHYLQKVNFSTR-FGDHVSFNKNGDALAIYDVMNWQPGSDRS-IRIHTVGVVREEPEKGLMLTLDED-AIYWNFE-TKKPPQSVCSESCPRGSRQATR
OlfCq6    423 KGPFS--KNSCAEISNLKPWQLVHYLQKVNFSTR-FGDHVSFNKNGDALAIYDVMNWQPGSDRS-IRIHTVGVVSEELEKGLMLTLDED-AIYWNFE-TKKPPQSVCSESCPRGSRRATR
OlfCq13   422 RGPFS--ENRCADITNLQPWQLVHYLQKVNFTTG-FGDHVSFDKNGDVLAIYDVMNWHPSSDGS-ISVRTVGVVNEGAASGKVLTLDED-AIYWNFK-IKKAPQSVCSESCPPGTRQATK
OlfCq14   424 RGPFS--GNSCADITNLKPWQLIHYLQKVNFITG-FGDRVSFDKNGDALPIYDVMNWQPSSDGS-IRVQTVGVVNKGVTSGMVLNLVED-AIYWNFE-TKKPPQSMCSESCPLGTRKARR
OlfCq12   423 RGPFS--GNKCADQINLQPWQVVHYLQKVNFTTG-FGDHVSFDENGDALAIYDVMNWQPSSDGA-ISVSTVGVVNEGASMKMVLTLKEN-SIFWNFK-NRKPPQSVCSESCPPGTRQVRR
OlfCq1    426 KGPF-----RCDGITNLKPWQLVHYLQKVNFTTG-FGDHVSFDENGDALAIYDVMNWHPSSDGS-IVVRTVGVVDEGASTGKVLTLEED-EIYWNTA-KNKPARSVCSESCPTGTRRTRR
OlfCq10P  430 RGPLS--ENSCADITDLKPWQLVPYLKKVNFTTG-FGDPVSFDNNGDALAIYDVLNWQPSSEES-IKLHTIGVVNDEVATGMVLRLNED-EXXXXXX-XXXAPRSVCSESCPPGTRKAMR
OlfCq9    430 RGPLS--EKNCADITDLKPWQLLSYLKKVNFTTG-FGDPVSFDNNGDALAIYDVLNWQPSSEGS-IKLHSIGVVNDEVATGMVLRLNKD-EIYWNFE-AQKAPRSVCSESCPPGTRKAMR
OlfCq5    430 RGPFS--GNSCADITDLKPWQLVHYLKKVNFTTS-FGDSVSFDNNGDALAIYDVLNWQPSSEGS-IKLHNIGVVNE-VATGMVLTLNND-EIYWNFE-AQKPPQSVCSESCLPGTSRAMR
OlfCq4    430 RGPFS--GNSCADITDLKPWQLVHYLKKVNFTTS-FGDSVSFDNNGDALAIYDVLNWQPSSEES-IKLHNIGVVNE-VATEMVLTLNND-EIYWNFE-AQKPPQSVCSESCLPGTRRAMR
OlfCq7P     1 ----------------------------------------------------------------------------------------------------XPPQSVCSESCLPGTRRVMR
OlfCq11   424 RGPFS--ENRCVDISNLKPWQLVHYLQRVKFTTG-FGDHVSFDKNGDALAIYDVLNWQLSSEES-VTVRRIGVVDEGVTTGKVFTLDEN-AIYWNFE-T-NPPRSVCSESCPPGTRQATR
OlfCq3    417 RGPFS--GNSCADITKLKPWQMVPYLQKVNFTTG-FGDHVSFDENGDALAIYDVMNWHPSSDGL-IVVRTVGVVDEGASAGRVLTLDED-AIYWNFE-TKKPPRSVCSESCPPGTRRAMR
OlfCq2    426 KGPFT--GNSCADISNLKPWQLVHYIQNVKFTTG-FGDHVSFDENGDALAIYDVMNWHPRSDGS-IIVHVVGVYEE-AILGKVLTLDED-ALFWNFE-TKKPPDSVCSESCPPGTRRARK
OlfCr1    433 NHTNSKIKPKCSSPDNITPAQLLQHVKDVHYTTQ-LGEEFYFLEGGIP-PVYDLVNWQIAPDGS-LQYAFIGHVDG-----NQLSINDS-AITWPGD-SGKVPISVCTSECPPGTRKAIK
OlfCt1    425 KGPFA--GGLCPNFSSMHPWQLLDYLKHVHFTND-FGEDTKFDINGDPVAMYDLINWQLSGKQE-MQYVTVGKYDETMK--PKLVIEEK-NIIWSGN-QKQVPLSVCSTSCPPGTRQATR
OlfCs2    421 RGPFE--NGQCPDVNQIKPRQLLHYLNAVNFTTP-VGELVYFEDNGEPSASYDIMNWHVDESGA-VNFVQVGQFDAANGPGQELNINIK-KVVWGGGWSDQVPVSVCSVSCLPGTRKSVQ
OlfCs1    426 RGPFE--NGQCPDATQVKPRQLLHYLNAVNFTTP-VGELVYFENNGEPSASYDIMNWYVNKSGE-VNFVQVGQYDAAKGPGQELNINIE-KVIWGGGWGNQVPVSVCSVSCLPGTRKVVQ
OlfCv3    420 KGPFS--NSTCANAPHIYPWQLQQYLQEVSFTIS--GEKVNFDMKGDSIPSYDLINWQRGSAGN-IEFINVGMFDGALESGQELVIKEE-AIMWPGH-QTEVLVSVCSNSCAPGFRKAVR
OlfCv2    414 HGPFE--NFSCANLNNVFPWQLKHYLEDVSFSIS--GQNVNFDNKGDSVPYYDLINWQRSASGD-MQFVKVGLYDGAQHSGKELVIEEQ-AITWSNQ-QTKVPVSVCSYRCAPGFRTAAC
OlfCv1    415 HGPFE--NLSCANLNNVFPWQLQHYLQEISFSIS--GEEVNFDIKGDAIPSYDLINWQRSASGD-MQFIKVGLYDGAQHSGKELVIEKQ-AIAWSNQ-QTKVPVSVCSNGCTPGYRKAVR
OlfCw1    431 HRSAL--TQQCPDIHNLQPWQVIEVLRKVNYTNM-FGDLIYFDENGDPVGSYDIVNWQKGGDDVPVQYITVGRFDSSLPKGQQLVLNQN-KIVWHGG-TNKVPVSVCSASCPPGYRKVRL
OlfCn1    421 NNL----SKMCLNVSQITPKQVSDQLERVNFIDE-YGENVFFDENGDPPASYELINWQLN-QGE-VQHVTVGYFSKSPDGTYKLTIKED-NVHWST--ENLIPKAVCSDTCPKGTRKAQI
OlfCu1    433 EQGS---DHSCNTESKFSPLQLLRYLKKVHFTNQ-FNEKVYFDTNGEPVPLYDIINWQKNARGT-INFQLVGTYDGSAPHGQQLKIEEG-LIRWTGG-QTKVPVSLCSPPCPPGTRQATR
OlfCx3    432 KGPFQ--NGTCGSLYQIQPWQLLYYMKQANFTI--LGEEVRFDENGDPIASYDLMNWQRGSDSS-LQLVRVGFYDASLEDDKDLVVEES-VIMWHR--AEKAPKSMCSQSCLPGTRKARQ
OlfCx2    432 KGPFQ--NGTCGSLFQIQPWQLLYYMKQTNFTT--LGEEIRFDENGDPIASYDLMNWQRGSDSS-LQLVRVGFYDASLEDDKDLVIDES-LIMWHR--AEKAPESLCSKSCLPGSRKARQ
OlfCx1    435 KGPFK--NGTCGSLYQILPWQLLYYMKRTNFTT--LGEEVRFDKNGDPIASYDLMNWQRESDGS-LQLVRVGIYDASFKDDKDLVIDES-VIMWHR--GDKAPESLCSKSCLPGSRKARQ
OlfCc1    430 SGPFE--NDTCADITNFEPWQLMYYLIHLRFTVPHTGEELFF-TNGEVEGFYELLNWQSDSNGG-ITYTHIGYYNSTAAPEDKLVINNN-SIIWNNN-VLKAPRSVCSERCQPGTRMGIR
OlfCa1    407 -------ERQCQDSTALQPWELLRQLRSITF-EN-GGKMYKFDANGDINLGYDLFLWEGDQSDE-HADDIIAEYDPTKGGSLYIHNDLS---------EIEKVVSRCSNSCQPGQYKKTA
OlfCb1P   402 -------DTTCSSETNFPPWQLVASMRRVNF-TL-DGNSYFFNENGDFTDGYDVIMWKENNE-E-RMIEPVGKFLIKKGDVEIFS-----EYHWI---NETLLSSSCSQFCQPGTVK--K
OlfCy1P     1 ------------------------------------------------------------------------------------------------------------------------
OlfCq15P  105 RGPS--------------------------------------------------------------------------------------------------------------------
```

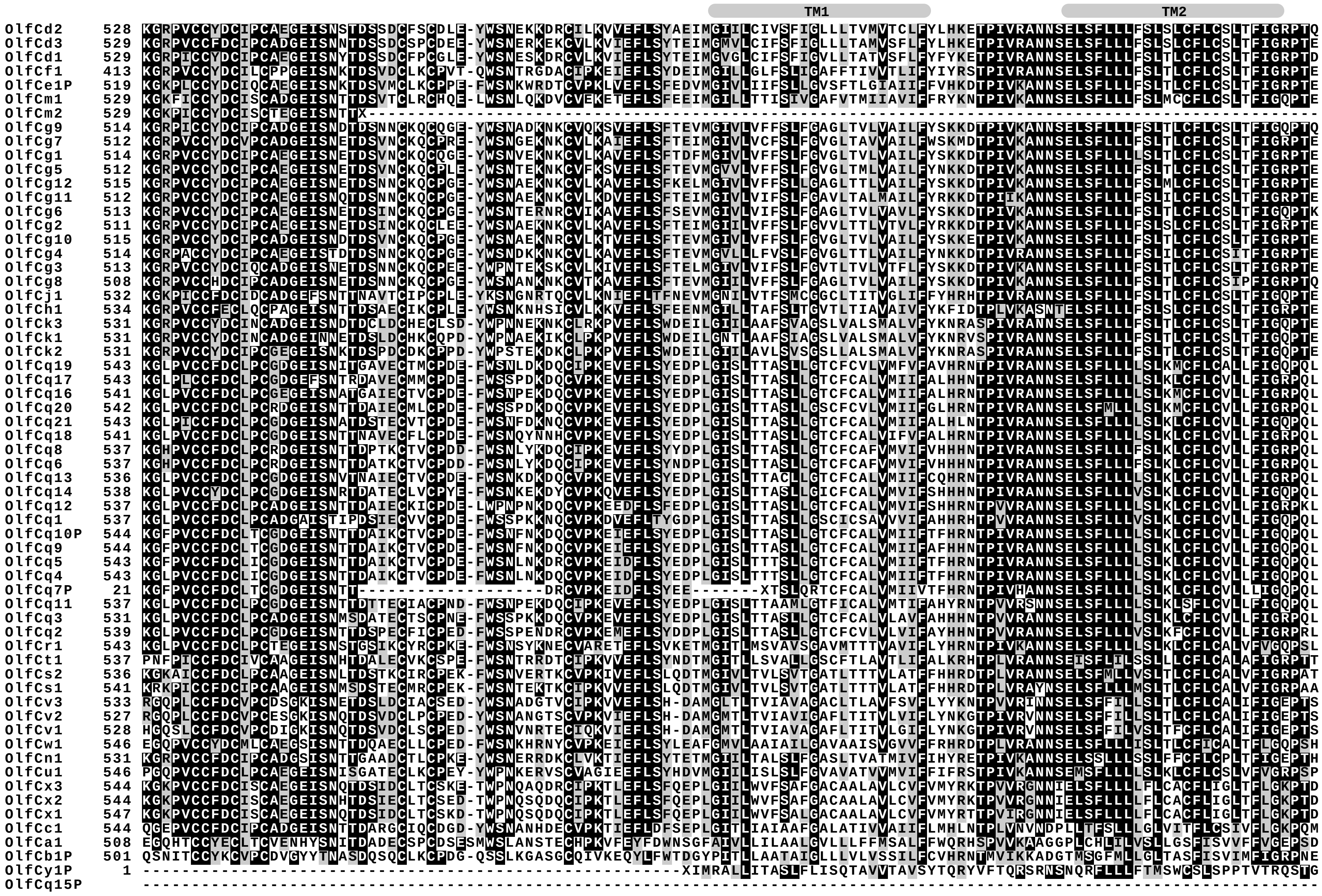
TM1
TM2
OlfCd2 528
OlfCd3 529
OlfCd1 529
OlfCf1 413
OlfCe1P 519
OlfCm1 529
OlfCm2 529
OlfCg9 514
OlfCg7 512
OlfCg1 514
OlfCg5 512
OlfCg12 515
OlfCg11 512
OlfCg6 513
OlfCg2 511
OlfCg10 515
OlfCg4 514
OlfCg3 513
OlfCg8 508
OlfCj1 532
OlfCh1 534
OlfCk3 531
OlfCk1 531
OlfCk2 531
OlfCq19 543
OlfCq17 543
OlfCq16 541
OlfCq20 542
OlfCq21 543
OlfCq18 541
OlfCq8 537
OlfCq6 537
OlfCq13 536
OlfCq14 538
OlfCq12 537
OlfCq1 537
OlfCq10P 544
OlfCq9 544
OlfCq5 543
OlfCq4 543
OlfCq7P 21
OlfCq11 537
OlfCq3 531
OlfCq2 539
OlfCr1 543
OlfCt1 537
OlfCs2 536
OlfCs1 541
OlfCv3 533
OlfCv2 527
OlfCv1 528
OlfCw1 546
OlfCn1 531
OlfCu1 546
OlfCx3 544
OlfCx2 544
OlfCx1 547
OlfCc1 544
OlfCa1 508
OlfCb1P 501
OlfCy1P 1
OlfCq15P

```
                    TM3                                                                          TM4
OlfCd2    647 WSCMLRHTAFGITFILCISCVLGKTIVVL--MAFRA-----------------------------TLP-GSNVMK-WFGPLQQRLSVVSLTLVQVIVCLLWLTLSPPYPYMNL-SYYREK
OlfCd3    648 WSCMLRHTAFGITFVLCISCVLGKTIVVL--MAFRA-----------------------------TLP-GSNVMK-WFGPTQQRLSVVFLTLIQVIICVVWLTVSPPFPYKNL-SYYREK
OlfCd1    648 WSCMLRHTAFGITFVLCISCVLGKTIVVL--MAFRA-----------------------------TLP-GSNVMK-WFGPLQQRLSVVSLTLIQVIICVLWLTMSPPFPFLNL-SYYREK
OlfCf1    532 WSCMLRHTAFGITFVLCISCVLGKTLVVL--MAFKA-----------------------------TLP-GSNVMK-WFGPLQQRLSVVSFTFIQLLICALWLILSPPFPYMNM-KHYQEK
OlfCe1P   638 WSCMLRHTAFGITFVLCISCVLGKTIVVL--MAFKA-----------------------------TLP-GSNVMK-WFGPLQQKLSVITFTLLQVVICVLWLTLSPPFPYMNM-NYYQER
OlfCm1    648 WSCMLRHTAFGITFVLCISCVLGKTIVVL--MAFKA-----------------------------TLP-GSNVMK-WFGPPQQRLSVLGFTLIQVLICVLWLTISPPFPFKNF-NYFKEK
OlfCm2        ------------------------------------------------------------------------------------------------------------------------
OlfCg9    633 WSCMLRHTAFGITFVLCISCVLGKTLLVL--MAFKA-----------------------------TLP-GSNIMK-WFGPVQQRLSVLAFTFIQVLICVLWLSISPPFPHKNM-KYYEEK
OlfCg7    631 WSCMLRHTAFGITFVLCISCVLGKTIVVL--MVFKA-----------------------------TLP-GSNVMK-WFGPTQQRLSVLAFTFIQVLICVLWLTISPPFPNKNM-TYYKEK
OlfCg1    633 WSCMLRHTAFGITFVLCISCVLGKTIVVL--MAFKA-----------------------------TLP-GSNVMK-WFGPVQQRLSVLAFTLIQVLICVLWLTMSSPFPYKNM-KYYKEK
OlfCg5    631 WSCMLRHTTFGITFVLCISCVLGKTIVVL--MAFKA-----------------------------TLP-GSNIMK-WFGPVQQRLSVLAFTLIQVIICVLWLIISPPFPYKNM-KYYKEK
OlfCg12   634 WSCMLRHTAFGITFVLCISCVLGKTIVVL--MAFKA-----------------------------TLP-GSDVMK-WFGPIQQRLSVLVITIVQVLICVLWLAVSPPLPYKNT-KYFKEK
OlfCg11   631 WSCMLRHTAFGITFVLCISCVLGKTIVVL--MAFKA-----------------------------ALP-GNNIMK-WFGPVQQRLSVFALTLIQVLICVLWLTMSPPFPHKNL-KYYQEK
OlfCg6    632 WSCMLRHTAFGITFVLCISCVLGKTIVVL--MAFKA-----------------------------TLP-GSNVMK-WFGPVQQRLSVFAFTLIQVLICVLWLTISPPFPYKNM-KYYKEK
OlfCg2    630 WSCMLRHTVFGITFVLCISCVLGKTIVVL--MAFKA-----------------------------TLP-GSNVMK-WFGPVQQRLSVLAFTLIQIIICVVWITLSPPFPYKNM-EYYKEK
OlfCg10   634 WSCMLRHTTFGITFVLCISCVLGKTVVVL--MAFRA-----------------------------THP-GKDIMK-WFGPVQQRLSVIALTLIQVLICVLWLTISPPYPYKNM-KYFKEK
OlfCg4    633 WSCMLRHTAFGITFVLCISCVLGKTIVVL--MAFKA-----------------------------THP-GSNVMK-WFGPVQQRLSVLVFTFIQVIICVLWLTISPPFPYRNM-KYYKEK
OlfCg3    632 WSCMLRHTTFGITFVLCISCILGKTIVVL--MAFRA-----------------------------TIP-GNNMIK-WFGPVQQRLSVLAFTLIQILICVVWLTISPPFPSKNI-KYYKEK
OlfCg8    627 WSCMLRHTAFGITFVLCISCILGKTIVVL--MAFKA-----------------------------TLP-GSNIMK-WFGPVQQRLSVLAFTLIQGLICVIWLTKSPPFPYKNM-KYFQEQ
OlfCj1    651 WSCMLRHTAFGITFVLCISCVLGKTLVVL--MAFRA-----------------------------TLP-GSNVMK-WFGPPQQRLSVFVFTFIQLLICMLWLTMSPPFPNKTT-NNYKDK
OlfCh1    653 GSCMLRHSSFGVTFALCMSCVLTRTIAVV--MAFKT-----------------------------TVP-GSGLPH-CSLPL-QRISVFCCTVFQVMICILWLALARPMPYKNS-MYSLDK
OlfCk3    650 WSCMLRHTAFGITFVLCISCVLGKTIVVL--MAFKA-----------------------------TLP-GSNVMK-WFGPSQQRLSVFGFTLVQIIICVLWLTISPPFPYKNM-QHYKDK
OlfCk1    650 WSCMLRHTAFGITFVLCISCVLGKTIVVL--MAFKA-----------------------------TLP-GSNVMK-WFGPPQQRLSVFGFTLVQIIICVLWLTISPPFPYKNM-QHYKDK
OlfCk2    650 WSCMLRHTAFGITFVLCISCVLGKTIVVL--MAFKA-----------------------------TLP-GSNVMK-WFGPPQQRLSVFGFTLVQIIICVLWLTISPPFPYKNM-QHYKDK
OlfCq19   662 WTCQLRHVVFGISFVLCISSILVKTMVVI--AVFKS-----------------------------SRPESKGAMK-WFGAVQQRCTVLVLTAIQVVICAVWLSTASPAPHKNN-QYIRSK
OlfCq17   662 WTCQLRHAVFGISFVLCVSSILVKTMVVI--AVFKS-----------------------------SRPEGKGAMK-WFGASQQRCTVLVLTALQVVICAVWLSTASPTPHKNN-HYFRSI
OlfCq16   660 WTCQLRHAVFGISFVLCISSILVKTMVVI--AVFKA-----------------------------SRPEGKGAMK-WFGATQQRCTVLVLTALQVVICVVWLLTASPTPHKNN-QYIRSK
OlfCq20   661 WTCQLRHAVFGISFVLCISSILVKTMVVI--AVFKS-----------------------------SRPEGKGAMK-WFGAAQQRCTVLVLTVLQVVICSVWLSTASPTPYKNN-QYIRSK
OlfCq21   662 WTCQLRHVVFGISFVLCISSILVKTMVVI--AVFKS-----------------------------SRPEGKGTMK-WFGAAQQRCTVLVLTALQVVICAVWLSNASPTPIKNN-QYIRSK
OlfCq18   660 WTCQLRHVVFGISFVLCMSSILVKTMVVI--AVFKS-----------------------------SRPEGKGAIK-WFGAVQQRSTVVVLTVLQVLICAVWLSTASPTPYKNN-QYIRSK
OlfCq8    656 WTCQLRHAVFGISFVLCISSILVKTMVVI--AVFKS-----------------------------SRPEGKKAMK-WFGAAQQRCTILVLTAIQVVICAVWLSTASPTPHKNN-LYIRSI
OlfCq6    656 WTCQLRHAVFGISFVLCISSILVKTMVVI--AVFKS-----------------------------SRPEGKMAMK-WFGAAQQRCTILALTAIQVVICVVWLSTASPTPHKNN-LYIRSI
OlfCq13   655 WTCQLRHAVFGISFVLCVSSILVKTMVVI--AVFKS-----------------------------SRPEGKGSMK-WFGTTQQRCTVLILTALQVVICIVWLSNSSPAPHKNS-QHISSK
OlfCq14   657 WTCQLRYAVFGISFVLSVCSILVKTMVVI--AVFKS-----------------------------SRPEGKDAMK-WFGLLQQRCTILVLTTIQVVICTVWISNASPTPHKNH-QYIRSK
OlfCq12   656 WTCQLRHAAFGISFVLCVSSILVKTMVVI--AVFKS-----------------------------SRPESKSAMK-WFGVAQQRGTVMALTTLQIVICTVWLSMASPKPYKNS-LYISSK
OlfCq1    656 WTCRLRHAVFGISFVLCISSILVKTMVVI--AVFKS-----------------------------SRPEGKSAMK-WFGSHQQRCTVLVLTALQVVICAVWLTNASPKPYKNN-QYTSSK
OlfCq10P  663 WTCQLRHAVFGISFVLCISSILVKTMVVI--AVFNS-----------------------------SRPEGTGAMK-WFGAAQQRCTVLVLTALQVVICAVWLSTSPPTPHKNS-QYVRSK
OlfCq9    663 WTCQLRHAVFGISFVLCISSILVKTMVVI--AVFNS-----------------------------SRPEGKGAMQ-WFGAAQQRCTVLVLTVLQVVICAVWLSTSSPTPHKNN-QYVRSK
OlfCq5    662 WTCQLRHAVFGISFVLCISSILVKTMVVI--AVFKS-----------------------------SRPEGSGAMK-WFGTAQQRCTVLVLTALQIVICAVWLSTSSPTPYKNN-QSIRSK
OlfCq4    662 WTCQLRHAVFGISFVLCISSILVKTMVVI--AVFKS-----------------------------SRPEGSGAMK-WFGTAQQRCTVLVLTALQIVICAVWLSTSSPTPYKNN-QSIRSK
OlfCq7P   115 WTGQLRHAVFGISFVLCISSILVKTMVVILXAVFNS-----------------------------SRPKGXGAMK-WFGTAQQRCTVLVLTALQIVICAVWLSTSSPTPCKNN-XIVRSK
OlfCq11   656 WTCQLRHAVFGISFVLCVSSILVKTMVVI--AVFKS-----------------------------SRPEGKNAMK-WFGAAQQRGTVLILTALQVLICAVWLSTASPTPHKNS-RYIRSI
OlfCq3    650 WTCQLRHAMFGISFVLCVSSILVKTMVVI--AVFKS-----------------------------SRPEGKSAVK-WFGAVQQRGTVLVLTAVQIVICVVWLSTASPTPHKNI-LYVRSK
OlfCq2    658 WTCQLRHAVFGISFVLCISSILVKTMVVI--AVFKT-----------------------------SRPEGKTSIK-WFGSAQQRGTVIALTSAQVAICTVWLSSASPTPHKNN-QYIHSK
OlfCr1    662 WSCRIQQAAFGISFVLCISCILVKTFVVL--IAFHS-----------------------------TRPESSALIK-WFGLGKQRGIVLVFTCVQVVICAIWLCVSPPLPYQNF-GMHRSK
OlfCt1    656 WSCGLRHTAFGIAFSLCLSCVLGKTLVVL--MAFKA-----------------------------SLP-GSNVMR-WFGPLQQRGIIFMCTAVQVVICCTWLCVAPPIPHMLM-THETAR
OlfCs2    655 WNCMLRHTLFGVSFVICIACILSKTVVVL--VAFQA-----------------------------TLP-GSNLMQ-YFGPIQQRAGILVCTMVQVVICLLWLLLAPPLPTERAGGEFGAR
OlfCs1    660 WNCMLRHTLFGVSFVTCIGCILSKTVVVL--VAFRA-----------------------------TRP-GSHLMQ-YFGPIQQRAAIFICTLVQVVICLLWLLLDPPQPTESA-GELGAR
OlfCv3    651 WSCMLRHTAFSITFSLCISCILGKTLVVL--AAFTA-----------------------------TRP-GNNLMK-WLGPTQQRIIIFCCTLIQVLICTVWLVASPPFPYRNT-KYQQSK
OlfCv2    645 WSCMLRHTAFSITFSLCISCILGKTLVVL--AAFTA-----------------------------TRP-GNNIMK-WLGPTQQRIIIFCCTLVQVLICTVWLVVSPPFPYRNT-KYQQSK
OlfCv1    646 WSCMLRHTAFSITFSLCISCILGKTLVVL--AAFTA-----------------------------TRP-GNNIMK-WLGPTQQRIIIFCCTLVQVLICTVWLVVSPPFPYRNT-KYQQSK
OlfCw1    665 WACPLRRISFGLTFALCLSCLLSKTLVVL--IAFKS-----------------------------TLP-GNNTAR-WFRPPQQRLGVFICSLLQGGVCIAWLTTASPYPVKNT-WLYRDR
OlfCn1    650 WSCMLRHTAFGLTFALCISCVLGKTIVVV--TAFRA-----------------------------TLP-GNKLSG-KFGPVQQRAIVFLCTAIQIVICALWLLIKPPFPDKAL-RYDHKK
OlfCu1    665 LTCRARQAAFGISFVLCISCILVKTIVVL--LAFRS-----------------------------TVP-GSISLK-VFGPPQQRVFIFCCTTGQVILCACWLALAPPYPYKNT-SYQDGR
OlfCx3    663 WLCQIRYPAFGISFTLCISCILAKTVVVL--MAFRA-----------------------------TIP-GNNVMK-WFGPVKQRSSVILCTCVQALICIIWLTTKPPLASYNS-KFMSET
OlfCx2    663 WLCQIRYPAFGISFTLCISCILAKTVVVL--MAFRA-----------------------------TIP-GNNVMK-WFGPVKQRSSVILCTCVQALICIIWLTTKPPLASYNS-KFMSET
OlfCx1    666 WLCQIRYPAFGISFTLCISCILAKTVVVL--MAFRA-----------------------------TIP-GNNVMK-WFGPVKQRSSVILCTCVQALICIIWLTTKPPLASYNS-KFLSAT
OlfCc1    663 WSCMTSQMALAVGFALILSSLMGKSALLM--LRARAVKAVKAAAKAAKAAAAASEQSPDTAVFAPAIP-QKNDVD-PIHPRHQRAIMILCTLIQVVGCTAWLILMPPHPVKNT-GVQNIK
OlfCa1    628 LTCRARQVIFGFSFTLCVSCILVKSLKIL--LAFEM-----------------------------NFE-LKELLC---MLYKPYMIVSVGMGVQIIICTVWLTLYKPFKDKEV---QTDS
OlfCb1P   620 HLCRAQQAVYSLGFTLCVSCILVKAFRTF--LAFLV-----------------------------FNP-QKQHE--LKKLYKPLIILVLLTGGQAIILLFWLILKSPYPDPLW-PGSGLI
OlfCy1P    66 CSCKLHHTAFGITFVLCLSCVLGKTIVVL--MAFKA-----------------------------TFP-GSDVINKWFGPLPHRLSVFALIQI---------------------------
OlfCq15P      ------------------------------------------------------------------------------------------------------------------------
```

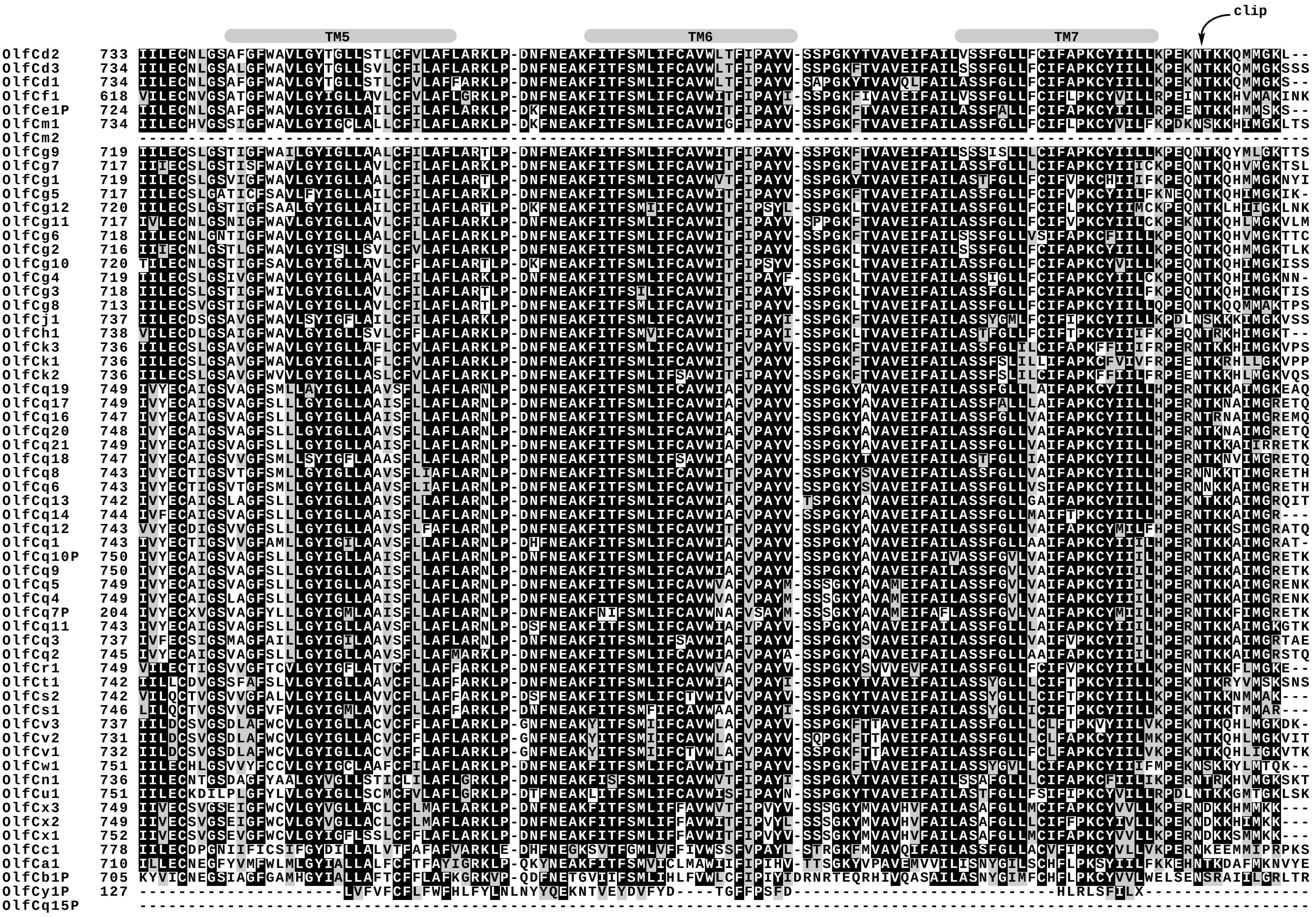
clip
TM5
TM6
TM7
OlfCd2 733
OlfCd3 734
OlfCd1 734
OlfCf1 618
OlfCe1P 724
OlfCm1 734
OlfCm2
OlfCg9 719
OlfCg7 717
OlfCg1 719
OlfCg5 717
OlfCg12 720
OlfCg11 717
OlfCg6 718
OlfCg2 716
OlfCg10 720
OlfCg4 719
OlfCg3 718
OlfCg8 713
OlfCj1 737
OlfCh1 738
OlfCk3 736
OlfCk1 736
OlfCk2 736
OlfCq19 749
OlfCq17 749
OlfCq16 747
OlfCq20 748
OlfCq21 749
OlfCq18 747
OlfCq8 743
OlfCq6 743
OlfCq13 742
OlfCq14 744
OlfCq12 743
OlfCq1 743
OlfCq10P 750
OlfCq9 750
OlfCq5 749
OlfCq4 749
OlfCq7P 204
OlfCq11 743
OlfCq3 737
OlfCq2 745
OlfCr1 749
OlfCt1 742
OlfCs2 742
OlfCs1 746
OlfCv3 737
OlfCv2 731
OlfCv1 732
OlfCw1 751
OlfCn1 736
OlfCu1 751
OlfCx3 749
OlfCx2 749
OlfCx1 752
OlfCc1 778
OlfCa1 710
OlfCb1P 705
OlfCy1P 127
OlfCq15P

```
OlfCd2         -------------------------------------------------------------------
OlfCd3     852 KTI----------------------------------------------------------------
OlfCd1         -------------------------------------------------------------------
OlfCf1     736 -------------------------------------------------------------------
OlfCe1P        -------------------------------------------------------------------
OlfCm1     852 N------------------------------------------------------------------
OlfCm2         -------------------------------------------------------------------
OlfCg9     837 KFY----------------------------------------------------------------
OlfCg7     835 KVQ----------------------------------------------------------------
OlfCg1     837 HTEK---------------------------------------------------------------
OlfCg5         -------------------------------------------------------------------
OlfCg12    838 -------------------------------------------------------------------
OlfCg11    835 -------------------------------------------------------------------
OlfCg6     836 SL-----------------------------------------------------------------
OlfCg2     834 SY-----------------------------------------------------------------
OlfCg10    838 KSY----------------------------------------------------------------
OlfCg4         -------------------------------------------------------------------
OlfCg3     836 KN-----------------------------------------------------------------
OlfCg8     831 KI-----------------------------------------------------------------
OlfCj1     855 RVF----------------------------------------------------------------
OlfCh1         -------------------------------------------------------------------
OlfCk3     854 KAL----------------------------------------------------------------
OlfCk1     854 KAL----------------------------------------------------------------
OlfCk2     854 KSY----------------------------------------------------------------
OlfCq19    867 NT-----------------------------------------------------------------
OlfCq17    867 NK-----------------------------------------------------------------
OlfCq16    865 NK-----------------------------------------------------------------
OlfCq20    866 KR-----------------------------------------------------------------
OlfCq21    867 KR-----------------------------------------------------------------
OlfCq18    865 NK-----------------------------------------------------------------
OlfCq8     861 -------------------------------------------------------------------
OlfCq6     861 -------------------------------------------------------------------
OlfCq13    860 K------------------------------------------------------------------
OlfCq14        -------------------------------------------------------------------
OlfCq12    861 IK-----------------------------------------------------------------
OlfCq1         -------------------------------------------------------------------
OlfCq10P   868 NN-----------------------------------------------------------------
OlfCq9     868 KY-----------------------------------------------------------------
OlfCq5     867 NK-----------------------------------------------------------------
OlfCq4     867 NK-----------------------------------------------------------------
OlfCq7P    322 NK-----------------------------------------------------------------
OlfCq11    861 NK-----------------------------------------------------------------
OlfCq3     855 K------------------------------------------------------------------
OlfCq2     863 NK-----------------------------------------------------------------
OlfCr1         -------------------------------------------------------------------
OlfCt1     860 -------------------------------------------------------------------
OlfCs2         -------------------------------------------------------------------
OlfCs1         -------------------------------------------------------------------
OlfCv3         -------------------------------------------------------------------
OlfCv2     849 -------------------------------------------------------------------
OlfCv1     850 -------------------------------------------------------------------
OlfCw1         -------------------------------------------------------------------
OlfCn1     854 SL-----------------------------------------------------------------
OlfCu1     869 -------------------------------------------------------------------
OlfCx3         -------------------------------------------------------------------
OlfCx2         -------------------------------------------------------------------
OlfCx1         -------------------------------------------------------------------
OlfCc1     896 RDIAAAAASSASLATTSSSGNPNGTTVSTLSLEE---------------------------------
OlfCa1     828 YARKSAENIKGLTGTEPQFKQENSVYTISNLSFVPEEKHE---------------------------
OlfCb1P    824 RIRDEMTSADIAVVTVSGIICEEVSAEISPVSKDPALKSVELFHAERGGAEQTVTHRVQTRRRHITK
OlfCy1P        -------------------------------------------------------------------
OlfCq15P       -------------------------------------------------------------------
```
