# Supplementary figures and images for: The repertoire of olfactory C family G protein-coupled receptors in zebrafish: candidate chemosensory receptors for amino acids
Source: BMC Genomics. 2006 Dec 8;7:309. doi: 10.1186/1471-2164-7-309 (PMC1764893; doi:10.1186/1471-2164-7-309)

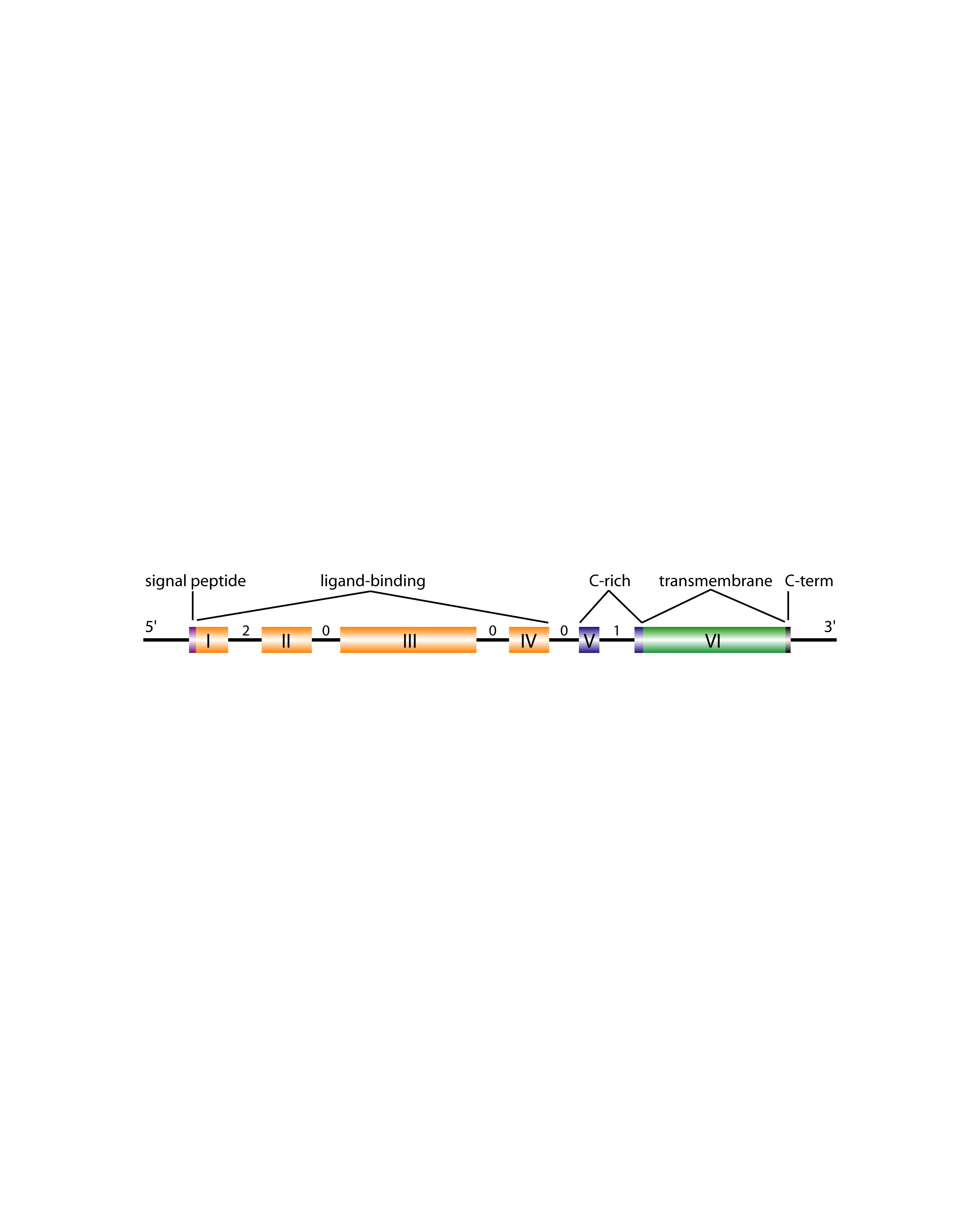

Supplement: Additional file 5 — Figure S2. Schematic representation of predicted OlfC gene structure. [file 1471-2164-7-309-S5.jpeg]
