# Supplementary material for: The repertoire of olfactory C family G protein-coupled receptors in zebrafish: candidate chemosensory receptors for amino acids
Source: BMC Genomics. 2006 Dec 8;7:309. doi: 10.1186/1471-2164-7-309 (PMC1764893; doi:10.1186/1471-2164-7-309)
Supplement: Additional file 6 — Figure S3. Multiple sequence alignment of predicted fugu OlfC amino acid sequences. [file 1471-2164-7-309-S6.pdf]

```
                                                                                           clip
                                                                                            +
594197    1 M------------WPEKVWGLFLVL-----------------------SFS------QAC-GQVCR-HIGEVEKPQL--SKEGDVILGGIFYFHNRWKTRENAYTAKPLPTECR-SL
744222    1 M----------PQVIAELFFIGLLKL----------------------TSA------QVR-ASSCQ-ILGSPEFPLL--SREGDVVIGGAFSVHSKVTQPSLSHQEKPAQISCS-SV
594233    1 M---------------QLAVGVLFVTL---------------------MAV------RGG-DLVCQ-TYGTKELSQF--SMEGDINIGGIFSFHQNPVTIDPALHFNPEMIQCE-GL
Ca12      1 M-----------PVCVCVMLLFALFHG---------------------AFG------AED-NLKCK-MLGRPEFPLL--SQEGDITIGGAFTLHSQMSKPSLSFEETPEDLTCS-RI
Ca02.1    1 M------------EISVIFLCFISLFDLNSAGDLKAPENSLKQQVGPREDTTGA------AAP-SDICR-LQGSARLPAF--SKDGDFVIGGVFSIHRYTVTVNHNYTTMPEPFRCR-VI
581784    1 M------------EISVLFLCFISLFDLNSAGDLKAPENSLKQQLGPREDTTGA------TAP-SVKCR-LQGSARLPAF--SKDGDFVIGGVFSIHRYTVTVNHNYTTMPEPFRCR-VI
584633    1 M------------EISVLFLCFISLCDLNSAGDLKAPGNSLKQQLGLREDTTGA------TAP-SVICR-LQGFARLPAF--SKNGDFLIGGVFSIHHYKVTVNHNYTTMPEPFRCRGSI
Ca09      1 M-------ADITGTLGLFFTLITLFVS---------------------SST------SFN-APTCK-LWRKFQLNEM--HEPGDVLLGGLFQVHYSSVFPEWTFTSEPHQPVCT-RF
589261    1 M-------------------------------------------------------------------------------------------------------------------
716738    1 M----------------------------------------------------------------------HKPGDMILGGLFEVHYTSVFPDLTFTSEPNQLVCQ-GF
Ca15.1    1 M----SQLSRIFTLIVGFGGRELGLGG---------------------VLQ------VVQ-ALTCS-QWSTPTEQGL--FQDGHVVVGGLFNLHYTPPDTANNFTQQSHYKACT-GL
571614    1 M--------------------GARA-----------------------GLD------VAG-AMLCS-HWGQRSDRNL--SADGDVMIGGLFNLYYIPSAVQQEYTQLPHYERCS-SL
744432    1 M------------ARTTVVLFIYCLLL---------------------MRT------MSK-TQTCT-VTGQTGFMEF--LKEGDLIIGGVFSMSSTRVLVDNDYMAIP-SAYCT-RW
Ca13      1 MSWMGWIPSQRGVQLLCLLCCMIIPVV---------------------IAL------LDQ-SQHCRVIPGSMSLPVL--EKRGDIILGGLFSLHDMVVEPNLPFTSTPPPTQCT-RF
611619    1 M-----------------------S-----------------------SVT------SPS-SSLCR-LQNSFQ-PGF--EANGDFIIGGMFPLHFNQEMPDLNNTYRPPVKCN-GF
611624    1 MDRSFLSCWGFTLLLWAISSIFFPVFS----------GLQHIAKEKNDTHGLEAGDGNDASPS-LPRCV-KTVDTQRPAL--HSRGDVMIGGIFPLHYSASVSQQKYINKPELITCS-GF
611613    1 M-----------------------------------------------------------------GDPENPQL--SKDGDIILGGIFSFHSSWINRRDTYMHKPLPLQCI-SL
735220    1 M----------DIGPFLLAFCLFPL-----------------------------------------HVSADSTCK-LKAKFNLSGYKNVEKKKVVIGGMFPVHMRVSSSGGNTSRLPVSSGCE-GF
179742    1 A-------------------------------------------------------------------------------PGDIIIGGIFPIHEDVDKE--TESFEPHIRPC-IRF

            +                                                     +                                      ++*                     + *
594197   73 NFRGFQFAQAMLFTINEINKSTDLLP-DVIVGYKIYDSCANIVNSIKLVLALTNRQDKES--------ASD-EESCT--KP-AQVQAIMGESSSSPCTAIASVIGPFHIPVISHFATCAC
744222   76 NLREFRFAQSMIFAIEEINKSDFLLP-NVSIGYRIYDTCGSTLSSVRAAMALMNGE------------STA-GKNCS--YR-SSVHAIIGESESFSTIVLSRLTGPFEIPVISHSATCEC
594233   71 DPGELQYAFTMMFAINEINNSSDLLP-GLILGYRIFDSCPSVPLSIRASLNLMNRY------------ESG-GDSCS--KL-SNVHAVIGETTSTSTIGIARTMGPFLIPVISHSATCAC
Ca12     75 NLREFRFAQTMIFAIEEINNSSSLLP-NISIGYKVFDTCGLTLPSTRAVMALMNGKT-----------RTP-EGGCS--SR-TSVHAIIGASESSSTIVMLQISGIFQIPVISHFATCAC
Ca02.1   98 DHHELQLSHAMVFAIEEINNSTELLP-GIKLGYQIHDSCAAVPIAVNVAFQLLNTLDPV---------FVT-GDNCS--QS-GMVMAVVGESGSTPSISISRVIGSFDIPLVSHFATCAC
581784   98 NHRELRLSHAMVFAIEEINNSTELLP-GIKLGYQIHDSCAAVPIAVHVAFQLLNTLDPV---------FVT-GDNCS--QS-GMVMAVVGESGSTPSISISRVIGSFDFPLVSHFATCAC
584633   99 DHHELRLSHAMVFAIDEINNSTELLP-GINLGYQIHDCCAAVPIAVHVAFQLLNTLDPV---------FVT-GDNCS--QS-GMVMAVVAESGSTPSISISRVIGSFDIPLVSHFATCAC
Ca09     79 DILGFRHAMTMAFAVQEINKNPDLLP-NLTLGYRLYDNCGALVVGFSGALALASGQEEA---------FAL-QGGCA--GS-PPVLGIVGDSLSTFTIASASVLGLYKMPMVSYFATCSC
589261    2 ---------TMAFAVEEINRNSNLLP-NLTLGYSLFDNCGALVVGFSGALSLASSPEMQ---------FLL-EEDCE--GP-PPVVGIVGDHYSTFSIAISSVLGLYKIPIVSYFATCSC
716738   39 DLAGFRHAMTMAFAIDEINRNISLLP-NVTLGYSLYDNCATLVIGFSAALSMVSGQQEQ---------FLQ-QEKCL--GT-PPVLGIVGDSFSTFSIATSDVIGLFKLPIVSYFATCSC
Ca15.1   82 ENLPLQYIYAMVFAVEEINHSAALLP-GVKLGYHIRDSCALHPWTTQAALALVAGDSASCELATPADYSAE-TSEEK--GA-ASVPLIIGGASSNAAKILLGTLSPL---SISYTASCPC
571614   64 DIESLKYMYTMVFAVEEINRDDSLLP-GVRLGYGIRDSCFRYPWALDGALSLVTGDSNSCNVAASAG-GNT-GAVEA--GE-KVVPLIIGAASSTTGIMLSSILQSL---SISYLASCPC
744432   73 NDRELKFARTAIFTVEEINRDGKLLP-GVTLGYRLYNGCGSENL-IRAAVEAVTGE-----------------GCS-----SQVQALLGHSSSGVSEDINLILSPFSIPQVSHLSTCAC
Ca13     87 SFRTFRWMQTMIFAVEEINRNAEILP-NITLGYKIYDSCSTPHQSLKAAIDLMGSEKDSQFEG-----KLQ-REGCD----GNVPAVIGDGGSTQSLVVARFLGVFHVPQVSYFSSCAC
611619   60 DPRAFRWAQTMKLAVEEINQRTDLLP-DHVLGYKLFDSCGYPLTGQRAALSLLNGPS-----------TDG-SSTCT--GA-APLLAVIGESSSSLSVMLSRILQPFKIPVISYFSSCAC
611624  106 DHRAFRWMMTMVFAVNEINNNSSLLP-GVKLGYRILDGCDHVPTSLQALLSLSMTWKQV---------EEM-IPACL--AD-SPVAAVIGLASSSPTGAAAHILGSFNIPLVSYFATCTC
611613   47 NFRGFQYAQAMLFAIDEINNSSDLLP-GITLGCKLYDSCRSIARGVRASLALINGGET----------TFKLSDECT--KP-AQVQAIMGETSSSPNMAVATVIGPFHVPLVP-VSVCSE
735220   74 NLRTFRWTRTMLFAIDEINKREDLLP-DTDLGYVIYDSCFTISKAVEGTLTYLTGQDE----------AVP-NYRCG--NG-PPLAALVGAGGSDLSIATARILGLYHFPQVSYSSTCSA
179742   35 QQSGFVLALAMINAIEDMNKSPPLADANITLGYRILDSCSDVSTALRATNDLMQQ-------------GNC-NSSGSSSSCGQPIMAVVGASYSETSIAIARQLTLPMIPQISYSSSAVL

                               *                     *
594197  180 LSDKNKYPSFLRTIPSDHYQSRALAQLVKYFGWTWVGAVRSNDDYGNNG-----------------MATFIETAEELG-ICVEYSVAVF-RTDPM----IKILQI-------------
744222  179 LSDRKEHPSFFRTIASDLYQSRALAQLVKHFGWTWVGAVNSDSDYGNNG-----------------MAIFLTAAQEEG-VCVEYTEKFH-RAEP-----EKLLKV-------------
594233  174 LGNRRDYPAFFRTIPSDIYQSQALAKLVKHFGWTWVGAIRTNSDYGNGG-----------------MTAFLKAAEKEG-VCVEYSVAIY-RTDPR----KWFLEV-------------
Ca12    179 LSNRKEYPSFFRTIPSDFYQSRALAKLVKHFGWTWVGAVKSDNDYGNNG-----------------LATFIMAAEQEG-VCVEYSEGFS-WTDPS----EQIARV-------------
Ca02.1  204 LSDKQKYPSFFRTIPSDQFQADALVKLIKHFGWTWIGAVCSDSDYGNNG-----------------MAAFLHAAQKEG-ICVEYSESFY-RTHPH----SRIKRV-------------
581784  204 LSDKQKYPSFFRTIPSDQFQADALAKLIKHFGWTWIGTVCSDSDYGNNG-----------------MAAFLHAAQKEG-ICVEYSESFY-RSHPH----SRIKRV-------------
584633  205 LSDKQKYPSFFRTIPSDQFQADALAKLIKHFGWTWIGTVCSDSDYGNNG-----------------MAAFLHAAQKEG-ICVEYSESFY-RTHPH----SRIKRV-------------
Ca09    185 LTNRQRFPSFFRTIPSDDFQVRAMIQILKHFGWTWVGLLVSDDDYGLHV-----------------ARSFQSDLVQSGQGCLAYLEVLP-WDNYL----SENRRI-------------
589261   99 LSDHQRFPSFFRTIPSDAFQVRAMIQILKHFGWTWVGLLVSDDDYGLHV-----------------ARSFQSDLVQSGQGCLAYLEVLP-WDGDP----SEIRRI-------------
716738  145 LSDRQRFPSFFRTIPSDAFQVRAVIQILKHFRWSWVGLLVSDDDYGLHV-----------------ARSFQSDLTRSGGGCLAYLEILP-WGYNP----GELTQV-------------
Ca15.1  194 LSDRHRYPTFFRTMASDIYQAQALAQLVLRFNWTWIGAVVANNDYGHVA-----------------VKVFQEQTQGKG-VCLAFVETLQ-RETIV----ADAVRA-------------
571614  175 LSDRAKFPTFFRTIPSDIYQARAMAQLAIRFRWTWLGAVVVNNDYGQLA-----------------IQIFQEEIRGKE-MCMEFIETVN-RETLT----TDARRI-------------
744432  168 LSDKKKYPTFFRTVPSDHFQISGLVQLLKFFDWRWVGIVYATGSYSDDG-----------------TAHFVKEAEKEG-ICVEYRLCFS-IASG-----EKSTAI-------------
Ca13    195 LSDKTQFPAFLRTMPSDLFQVGALVQLVKYFGWTWVGVIAGDDAYGRGG-----------------AAIFANEVRRLG-ACIALYEMIP-KTQSQ----AAISSI-------------
611619  164 LSDKRKYPNFFRVIPNDDYQVKAIAQLVHFNWTWVGLLLCERDYGRFA-----------------AEGLLRELKSTK-VCVAYQEIIP-LVYNQ----LRVQAI-------------
611624  212 LSDKSTYPSFLRTVPSDLFQVRGLVQMVTFMSWLWVGTIGTTDDYSHYG-----------------IQAFSHQLRQQG-GCVEFQLTIP-KSPTA----AELKEM-------------
611613  152 KCPPGTRKVLQKGKPVCCYDCLRCAEGEISNSTDSISCVRCHSDFWSNERRDTCIKKKEEFLSYEEMMGALLTAASLLG-TCLTAVVMFIFFRYRR----TPIVRANNSELSFLLLFSLT
735220  179 LKSKFQFPTFLRTIPNDQHQSTAMAKLVIHFGWTWVGTISADDDYGKYG-----------------IKDFKEQVEEAG-VCISFSETLP-KVNSP----ENIQRI-------------
179742  141 LSDKTHFPAFMRTIPNDKYQTTAMITLLSHYGWNWVGIITTDGSYGLSA-----------------LDQFVSQASAKG-ICVAFKSILP-QSVSSQDTSSAITKT-------------
```

```
                                ++                                       *+
594197   262 ------IDII-KSS-------TSKVIVTFLSPGDLNVLLQEFSQHNLT----------GYQWVGSESWISDSHIAA-MDVHHILDGAVGLSIPKAHVTGMKEFIMDVK-QL---SSSS-K
744222   260 ------VEVI-RRG-------TARVIVGFLAYVEMNNLLQQLSLHNVT----------GLQFVGVEAWITANSLVT-PTSFGVLGGSLGFAVEKAAISDLDDFL----------------
594233   256 ------VDII-KKS-------TSKVIVAFVDGTDLDILVKELHAQSVT----------GLQWVGSEGWITYRFIAS-PENYAVVQGAVGFAALNTHLPGLQEFLADSR-PS---TTPG-N
Ca12     261 ------VTVI-KSG-------SARVLVAFLAQSEMSALLEEAVKQNLT----------GLQWVGSESWITAGHLAL-KKYSAILTGSLGFTIRKTKITGLQEFLLQVN-PS---QNPQ-N
Ca02.1   286 ------ADVI-RRS-------TAVVVVAFTASTEMMILLEELSHEPSP----------PRQWIGSESWVTDPDLLR----FSFCAGTIGFAIQRSVIPGLRDFLLDLS-PS---KVAS-S
581784   286 ------ADVI-RRS-------TAIIVVAFTSSGDLRILLEELSREPSP----------PRQWIGSESWVTDLDLLR----FSFCAGTIGFAIQRSVIPGLRDFLLDLS-PS---KVAS-S
584633   287 ------ADVI-RRS-------TAIIVVAFTSSGDLRILLEELSREPSP----------PRQWIGSESWVTDSEILR----FSFCAGTIGFAIQRSVIPGLRDFLLDLS-PS---KVAS-S
Ca09     268 ------VHVI-KES-------TARVLMVFAHQSHMIHLMEEVVRQKVT----------GLQWLASEAWTGTTFLQT-PDFMPYLNGTLGIAIRRGEITGLRDFLLRIR-PGQSSNNTS-Y
589261   182 ------VHVI-KES-------TARVLMVFAHEIHMIQLMDEVVGQNVT----------GRQWVASEALTTAAVLQV-PHFMPYLRGMLGIAIRRGEIPGLREFLRQVR-PDRHSRDTE-T
716738   228 ------VEVM-KKS-------TARVVIVFAHQIHMIQLMEEVVKQNLT----------GLQWIASEAWTAAAVLQT-TELMPYLGGTLGIAIRRGEIPGLRDFLLGLH-PDLHEISNR-N
Ca15.1   276 ------ARTI-QAS-------TARVILVFSWYTDVGHLFRQLQKINVT----------DRQFLASEAWSTSEVLLKDPDTSTVASGVVGVAIASQHIPGFDRFLRGLN-PS---LRPS-D
571614   257 ------ALTI-QAA-------TARVILIFCWYIDAKEILLELAKRNIT----------GRQFLASEALSTSEELLQELAIAEVANGVLGVAVQSSTIPGFEHFLRSLN-PV---QRPD-D
744432   249 ------VKAL-QES-------SSRVVLLFMSMFKTKAFLNEMENNNIT----------DKQWLGSESWITQADLAS-SKRQHILQGAIGFALPQMVIPGLGDFLLSLK-PS---DEPQ-S
Ca13     277 ------ISNI-RSS-------GARVVLVFAVEQDVARLFDEAVRQKLT----------GIQWLASEAWSTAAILSTPKRYHHILQGSMGFAIRRADIPGLQDFLLRLH-PSSA-EADD-D
611619   246 ------MQVM-RTS-------SAKVVVVFSAEVEMIPLLRDYMKQNIT----------GIQWIASEAWVTASVFTG-SKYYPYLGGTIGLGIRKGHIPRLSDYLLTVN-PQ---VYPN-N
611624   294 ------ADRL-QSS-------TARVVVVFATEGQLLEFFLELIYRNMT----------GIQWVASEAWVTASLLTT-PRFHALLEGTLGFSFPGAEIPGLKEFLLNIC-PS---PKPG-M
611613   267 LCFLCSLSFIGRPSGWSCMLRHAAFGITFVLCISIKLLAQELYSQNIT----------GLQWVGSDAWITDHSLTD-SSGHSILLGSLGFTVSKAKILGLEEHLRGLH-PA---QFPT-S
735220   261 ------IQTL-VKS-------TAKIIVVFSSDVDLSPLITELLRHNIT----------NRTWIASEAWVTSALMLK-PGASLLLGGTLGFAVKRASIPGLQHYLLDLD-PY------G-D
179742   227 ------ARTIYKNP-------KVQVIISFAKPSQMKFLFHKLKSMMLKPGETNGEGRMRRVWVASDSWSTSRYIYG-NLTLEDIGYVLGFTFKSGNVSSFREYLEQLGAPE---ENIKIN

                                                            ++
594197   352 ELFREFWEALFDCKFEDSVST----TTENQRECSGHEDLAGVKNIFTDMSLMPIFYNIYKGVYAVAHALHDILSC--------NNTCNKTAQLDPFTILQHIRSTHFKTK-EGDEVYFNE
744222   339 --IGDFWETEFECKETIED------GMAGPATCQENGDLAAFKGYADDVAELRYSGNIYKAVYAVAHSLHSTLQCSI------SGVCDKTVKVTHQQILQHIKRIRFKTK-EGDEVYFNE
594233   346 NGLVELWETVFKCTLSPRA-------QSPLAACTGKESLRDASTRFTDVSDASLLNNVYKATYAIAHALHLLLTCKEGEGPFENNTCADRHNVQPWQVLHYLTLVNFTTK-IGDNVHFDE
Ca12     351 NLLKEFWETTFGCSFQSDV--------HGATQCSGVEKLKDIQNPFTDVSELRISNNVYKAVYAVAHAMHSMLKCGQ-SGEAVNQSCTTKKDFELKQVVEHLQSVNFTLQ-SGERVYFDD
Ca02.1   373 PVLTEFWEDSFNCRLG-----------KGERMCDGSEDIMTLQSPYTDTSELRITNMVYKAVYAIAHAIHNAV-CQDTNA---TTRCSKFTTINPKKVLTQLKTVNFSQ--NGYAVSFDA
581784   373 PVLTEFWEDSFNCRLG-----------KGERMCDGSEDIMTLQSPYTDTSELRITNMVYKAVYAIAHAIHNAV-CQDTNA---TTRCSKFTTINPKKVLTQLKTVNFSQ--NGYAVSFDA
584633   374 PVLTEFWEDSFNCRLG-----------KGERMCDGSEDIMTLQSPYTDTSELRITNMVYKAVYAIAHAIHNAV-CQDTNA---TTRCSKFTTINPKKVLAQLKTVNFSQ--NGYAVSFDA
Ca09     361 DMVQQFWEYSFQCKFGAS---------GSAEACTGDENIQQVDAEFLDVSNLRPEYNIYKAVYALAYALDDMLQCEPGRGPFSGGSCADIHKLEPWQFVHYLQHVNFTTT-FGDQVSFDE
589261   275 SMVRQFWEYIFQCKFDPPGL-----VEAGEVLCTGQEAIEDADTEFLDLSNLRPEYNVYKAVYALAYALDDALRCEPGRGPFSAHSCAENQRLQPWQLVYSLEKVNFTTS-FGDQVSFDE
716738   321 SLVRQFWEYTFQCRFAPAPTGW---VEGGGLLCTGEEELGRVQTEFLDVSNLRPEYNIYKAVYALAHALDEMLHCVPGRGPFDEHRCATLESLKPWQLMHYLQQVNFTTA-FGDEVSFDE
Ca15.1   367 KFLQEFWEEEFGCSPSPPSSETSGDLNASLPPCSGAESLEGVQHPFTDTSHLRVTYNVYLAVYAAANALHSLLSCPIHNSPSGTSHCTSPKGIKTTELLQHLSKVNFTTP-QGKHLYF-R
571614   348 EFLKDFWEMEFKCSPVLQLLHSC--LFASLPPCSGAESLVEMEHPFTDTSKLRVAHNVYLAVYAAAHALHSLLSCPGQDSPPGKSNCSSPNHIRPIDVLQHLNRVNFSTP-RGETFYF-Q
744432   339 DIVKAMWGDFFNCSFS---------PSSTSAACTGTEDLRTVTNDYTEVTHFRAENNVYKAVYLVAYALQGLLQCENGSNPTTGKPCVNKKEVQPKLVLEHIKYVNFTTK-YGSKVFFDK
Ca13     370 PFLIPFWEEVFQCSLDPHG------HSEAKRPCSGTEELRSVKNIYSDVSQLRISYNVYKAVYALAYAIKAMRSCEKGSGPFSQQACPDLDNIHPWQLHHYIKQVNYTNR-FGDEIKFDE
611619   336 PLVKELWETLYGCRSSLSS-------SSHVPLCTGQEPLLEQHSAFMNTSSPRVTYNVYKAVYAIAHSLHNLFLCQPGPGPFKNNTCAQRNNVQPWQLQHSLQEVAFKIS--GEVVDFDQ
611624   384 EFVNMFWEDLFGCKLNSDTK-----SADESPVCTGSEDLRYTQSSYTDVSQVRISYNVYKAVYAIAHALHSLLNCESPGSN--VGTCKKREPFTSKQLLQHLKSVNFTNQ-FKEKVYFDA
611613   371 AFMTEFWEDRFACSLNITG-------STGRRPCSGSESLQNDTSPFTDVTELRFTNNVYKSVYVVAHALDNLMKCEKGEGPFSEGSCADPKHIQPWQVLHYINTVRFNTS-EGETVYFER
735220   348 TLSEEFWETVHG-------------------LCTGLETVAQLKNTYSDVSQLRITYSVYKAVYAVAHALHNLEHCEQGRGPFNGRDCTNISSFEPWQLMYYLKNVRYTVPHTGEEIYFD-
179742   330 PFLQEFYMHMNATA----------------VGSGEDKHVPEALRSLWEHVHADLIFSVEMAVSAITQAVATICR---------RTDCKTLGSVQPWQVLAALWMQEF-KL-RQKSYKFDS

594197   459 NGDPPAKYEIINWQPTENGNVEFVPVGLYDASLPAD-RQLTLTNR-TLVWAQKKSLQ-VPVSVCSEKCPPGTRKVLQKGKPVCCYDCLRCADGEISNSTDSISCVRCHSE-FWSNERRDA
744222   444 NGDPPAKYEIINWQPTENGNVEFVPVGLYDASLPAD-RQLTLTNR-TLVWAQN-SEQ-VPVSVCSEKCPPGTRKVLQKGKPVCCYDCLRCADGEISNSTDSISCVRCHSE-FWSNERRDA
594233   458 MGDPVPRYALVNWQMDEAGYVLFETIGDYDASRPEG-HQFQMNDGVKALWAGE-NLE-VPRSVCSESCLPGTRRAFVKGRPICCFDCIACADGKFSNSTNAVKCDKCPPE-YKSNEERNN
Ca12     461 YGDPAATYELVNWQRSPEGNTVFVVVGNYDASQPNG-RQFTMNNI-NITWAAR-LQK-RPLSVCSQSCIPGFRQAVIKGKPICCFTCVACAAGEISNSSNSAECLQCPLE-FWSNEDHSQ
Ca02.1   476 NGDPVASYELVNWKKSGSGSIEVVPVGYYDASLPEG-QEFRIFR--DITWVDG-RKQ-VPVSVCSDSCPQGTRKVLQKGKPICCYDCVQCPEGEISNVTDSPECIPCLDD-FWPNPERNA
581784   476 NGDPVASYELINWKKSGSGSIEVVPVGYYDASLPEG-QEFRIFR--DITWVDG-RKQ-VPVSVCSDSCPQGTRKVLQKGKPICCYDCVQCPEGEISNVTDSPECIPCLDD-FWPNPERNA
584633   477 NGDPVASYELVNWKKSGSGSIEVVPVGYYDASLPEG-QEFRIFR--DITWVDG-RKQ-VPVSVCSDSCPQGTRKVLQKGKPICCYDCVQCPEGEISNVTDSPECIPCLDD-FWPNPERNA
Ca09     471 NGDVLPIYDILNWQWLPDGRTQVQNVGEVKRSPSRG-EELQIHED-KIFWNFE-SNK-PPHSVCSESCPPGTRMSRKKGQPVCCFDCLLCSEGKISNTTDSMECTSCPED-FWSSPQRDH
589261   389 NGDVLPIYDIMNWLWLPDGRTKVQNVGEVKGSPFRG-EELTLYED-KIFWNFE-SNK-PPQSVCSESCPPGTRMSRKKGQPVCCFDCLPCSEGEISNTTDSMECTSCPED-FWSSPQRDH
716738   437 NGDALPIYDIMNWLHGHNSAYKI----------------CSRDEE-NLRWRKE-EEE-SSLSA--------------------ALTVFLGSEGEISNTTDSMECTSCPED-FWSSPQRDH
Ca15.1   485 GADIPAMYDLINWQSGTDGTLQLVLIGAVAG------FDLQLNES-EIEWSAK-YNQ-VPVSVCSESCPPGTRKANRKGEPLCCFDCIPCADGEISNTSGSLQCDRCPPE-FWSNDGRTA
571614   464 GSDMTARYDLVNWQKTPNGPLKLVLVGRVDG------FDLILNES-AIQWSTG-LNQVVPVSVCSESCPPGTRKANRKGEPLCCFDCIPCADGEISNTSGSLQCDRCPPE-FWSNDGRTA
744432   449 NENSVAQYDLVNWQMREDGSVDIVTIGQYDTSFKGE-ELKVADDA-KIVWGGK-YKQ-VPRSVCREPCPPGTRKAINKLKPVCCFDCFECPDGTFSNQTDSLDCFSCPQE-LWPNDQKNL
Ca13     483 NGDPAAMYDLINWQLTPGGDMDFVTVGKFDDIAGTGRKNLHIEEE-KIVWNGN-NTQ-VPLSVCSSICPPGTRKAIRPNYPICCHDCVVCTAGEISNQTDAIECARCLPE-FWSNADRTA
611619   447 RGDSIPYYDIINWQRGVDGSIEFVNVGLFDGSKPTG-KELVIQED-RIVWAGH-QRE-ASLSICSASCLPGTRKAIRKEVPVCCFDCIPCDTGKISNETDSVDCTFCPED-FWPNPNRTV
611624   496 KGEPVPLYDIINWQKDSVSNIRFVKVGSYDGSAPLK-QQLQMDQN-AIVWTGG-KSK-VPVSLCAAPCPPGSRQARRPGQPHCCFDCLPCADGQISNQTGSTECQRCPEY-YWPDKDKVK
611613   483 SGDSPARYELVNIQLTDKGTLEGRTVGIYDASLPED-HQFIMSDE-PIVWGNG-EME-APVSVCTQRCLPGTHKVLQKGKPVCCFDCVPCPPGEISNSTNSIHCIKCPNL-FWSNQERDA
735220   448 DGDVEGFYDIINWQFNSDGEISYVKVGHFNATAVPE-EQMTIMNG-SIVWNSD-ALE-PPRSVCSDNCQPGTRKGIRQGEPVCCFDCIPCADGEISNTTNARECILCGED-DWSNEAHDA
179742   423 SGDINMGYEIVMWTSVTSEISVHHVVAEYHPLYSNI-TFMEQHNF-TTKQLLD-DLK-QVVSKCSNSCIPGQFKKTSEGQHTCCYECINCTENYYSNSTDMDQCLSCDADTEWSPKGSSS
```

```
TM1 TM2 TM3
594197  575 CIKKKEEFLSYEEMMGALLTAASLLGTCLTAVVMFIFFRYRQTPIVRANNSELSFLLLFSLTLCFLCSLTFIGRPSGWSCMLRHTAFGITFVLCISCVLGKTMVVLMAFRATLP-GSNVM
744222  559 CIKKKEEFLSYEEMMGALLTAASLLGTCLTAVVMFIFFRYRRTPIVRANNSELSFLLLFSLTLCFLCSLTFIGRPSGWSCMLRHAAFGITFVICISCVLGKTMVVLMAFRATLP-GSNVM
594233  574 CNLKAIEFLTFRELMGILLVAFSVFGACLSTTIALIFFHFRQTPIVRANNSELSFLLLFSLTLCFLCSLTFIGRPSEWSCMLRHTAFGITFVLCISCILGKTMVVLMAFRATLP-GSNMM
Ca12    576 CVPKVIEFLSFEETMGALLAAVSLFGAALTSLVFCVFFRFRHTPLVKASNSELSFLLLFSLTLCFLCSLTFIGRPSRWSCVLRHTAFGITFALCMSCVLAKTVAVLFAFTAKRP-GNTVF
Ca02.1  590 CFPKPVEFLSFNEVLGIILAVFSVGGACLAVITAAVFFHHRTSPIVRANNSELSFLLLFSLTLCFLCSLTFIGAPSHLSCMLRHTAFGITFVLCISCVLGKTVVVLMAFRATLP-GSNVM
581784  590 CFLKPVEFLSFNEVLGIILAVFSVGGACLAVITAAVFFHHRASPIVRANNSELSFLLLFSLTLCFLCSLTFIGAPSHLSCMLRHTAFGITFVLCISCILGKTVVVLMAFRATLP-GSNVM
584633  591 CFLKPVEFLSFNEVLGIILAVFSVGGACLAVITAAVFFHHRTSPIVRANNSELSFLLLFSLTLCFLCSLTFIGAPSHLSCMLRHTAFGITFVLCISCVLGKTVVVLMAFRATLP-GSNVM
Ca09    586 CVPKKTEFLSYHEPLGICLTAASLLGTVISVVVLGIFIHHRSTPVVRANNSELSFLLLVSLKLCFLCSLLFIGRPRLWTCQLRHAAFGISFVLCVSCILVKTMVVLAVFRASKPGGGATL
589261  504 CVPKKTEFLSYHEPLGICLTAASLLGTVISAVVLGIFIHHRSTPVVRANNSELSYQILVSLKLCFLCSLLFIGRPRLWTCQLRHAAFGISFVLCVSCILVKTMVVLAVFRASKPGGGATL
716738  517 CVPKKTEFLSYHEPLGICLTAASLLGTVISAVVLGIFIHHRSTPVVRANNSELSFLLLVSLKLCFLCSLLFIGRPRLWTCQLRHAAFGISFVLCVSCILVKTMVVLAVFRASKPGGGTTL
Ca15.1  595 CVPRQLDFLSFNETLGVALTAVAVSGAVVTTAVFVVFLHYRHTPMVRANNSELSFLLLLSLKLCFLCSLVFIGRPSVWSCRFQQAAFGISFVLCVSCLQVKTIVVLAAFRSARPGAGALM
571614  575 CVPRQLDFLSFNETLGVALTAVAVSGAVVTTAVFVVFLHYRHTPMVRANNSELSFLLLLSLKLCFLCSLVFIGRPSVWSCRFQQAAFGISFVLCVSCILVKTLVVLAVFRSAQPDCKATM
744432  564 CLPKPSEYLSYREITGALLSGFGCLGVFLSLLTTLIFLVHKETPIVKANNSELSFLLLLSLKLCFLCSLTFIGRPSEWSCMLRHTAFGITFVLCISCVLGKTVVVLMAFRATLP-GSKVM
Ca13    599 CVPKQVEFLSFGDTIGIALLVVSLIGSFLTCAVALVFFYHRTSPIVRANNSDLSFLLLFSLTLCFLCSLTFISPPSQWSCMLRHTAFGITFVLCISCILGKTIVVLMAFRATLP-GSDVM
611619  562 CIPKKVEYLAY-DSLGIALMVTSVVGACATIATFAVFFYHRNTAIVRVNNAELSFFILLSLVLCFLCSLVFIGEPTTWSCMLRHTAFSITFSLCFSCILGKTLVVLAAFTATRP-GHNIM
611624  611 CLPGIEEFLSFSETMGIILVILTLVGVLLTFSLTIIFLHFRSTPIVKANNSEISFLLLLSLKLCFLCSLLFIGQPSLWKCRLRQAAFGISFVLCLSCLLVKTIVVLFAFRTNLRAGCCAL
611613  598 CIPKSIEFLAHKELLGTLLVLFSLLGVFLTTFMFLIFYCHKETPIVRANNSELSFLLLFSLTLCFLCSLTFIGRPSGWSCMLRHTAFGITFVLCISCVLGKTMVVLMAFRATLP-GSNML
735220  563 CVPKIIEFLAFGEPLGITLIVISAFGALVTIAVGVVFIVNVGTPLVEANDAVLSLSLLFSLVVTFLCSIVFLGEPQNWSCMTSQVALALGFALCLSCIMSDLF---QPFRL----HNEKC
179742  539 CISKEQLFFSWNDIFAVVLLAFSALGILLCLLTSALFLYQRDTPVVKAAGGPLSQAILFSLVVSYISAMLFVGEPSSLQCKARQVLFGISFTLCVSCILVKTLQILLAFQFNPA-LQNML

TM4 TM5 TM6
594197  694 KWFGPAQQRLCVLGFTLIQ--VIICILWLSISPPSPNKNFNVA--KDRIILECALGSALGFWAVLGYIGLLAMFCFILAFLARKLPDNFNEAKFITFSMLIFCAVWVTFIPAYVSSPGKF
744222  678 KWFGPAQQRLSVLGFTLIQ--AFICLLWLTISPPFPFKNIKDF--KDRIILECALGSAVGFWAVLGYIGLLAMLCFFLAFLARKLPDNFNEAKFITFSMLIFCAVWVTFIPAYVSSPGKF
594233  693 KWFGAAQQRLSVLTFTLVQ--VLICILWLTINPPFPFKNTKHY--KDKIILECALGSALGFWAVLGYIGLLAVLCFVLAFLARKLPDNFNEAKFITFSMLIFCAVWITFIPAYASSPGKF
Ca12    695 YCSVPL-QRTSVFACITLQ--VIICVLWLTLAPPHPHKNTAHA--KERIILECNLGSPVWFWVVLGYIGLLAVICFILAFLARKLPDNFNEAKFITFSMLIFCAVWVTFIPAYVSSPGKF
Ca02.1  709 KWFGPPQQRMTVVTFTSIQ--VLICIVWLVVNPPFPVRNLTTY--KERIILECALGSSVGFWAVLGYIGLLAAVCLVLAVLARKLPDNFNEAKMITFSMLIFCAVWITFIPAYVSSPGKF
581784  709 KWFGPPQQRMTVVTFTSIQ--VLICIVWLVVSPPFPVRNLTTY--KERIILECALGSSVGFWAVLGYIGLLAAVCLVLAVLARKLPDNFNEAKMITFSMLIFCAVWITFIPAYVSSPGKF
584633  710 KWFGPPQQRMTVVTFTSIQ--VLICIVWLVVSPPFPVRNLTTY--KERIILECALGSSVGFWAVLGYIGLLAAVCLVLAVLARKLPDNFNEAKMITFSMLIFCAVWITFIPAYVSSPGKF
Ca09    706 KWFGAVQQRGTVLGLTSIQ--AAICFAWLLSSSPKPHKNIQYH--KDKIVFECVVGSTVGFAVLLSYIGLLAILSFLLAFLARNLPDNFNEAKLITFSMLIFCAVWVAFVPAYINSPGKY
589261  624 KWFGAVQQRMTVIILTSIQ--AAICLSWILLASPFPHKNTQYS--NERIIYECAVGSTVGFILLLSYIGLLATLSFLIAFYSRKLPDSFNEAKLITFSMLIFCAVWVAFVPAYISSPGKY
716738  637 KWFGAVQQRGTVLGLTSVQ--AAICTAWLVSYSPEPHKNTQYY--NYKIVYECVVGSPIGFSVLMGYIGLLAVLSFQLAFLARNLPDNFNEAKLITFSMLIFCAVWVAFVPAYINSPGKY
Ca15.1  715 KWFGPSQQRGSVCIFTCVQARVIICIVWLSLSPPVPQADLDVP--GLQVTLECAMASVVGFSLVLGYIGLLACTCLLLAFLARKLPDNFNEAKLITFSMLIFCAVWVAFVPAYISSPGKY
571614  695 KWFGPSQQRGSVGLFTSIQ--IVICGIWLSVSPPKPERDLGFQ--GSKVTLECAMASVVGFSLVLGYIGLLACTCLLLAFLARKLPDNFNEAKLITFSMLIFCAVWVAFVPAYVSSPGKY
744432  683 KWFGPKQQRLTVLTFTLIQ--VVICILWLTTNPPFPMRNMTYY--KDKIILECALGSAVGFWAVLSYIGVLAILCFILAFLARKLPDTFNEAKLITFSMLIFCAVWITFIPAYISSPGKF
Ca13    718 KWFGPGKQKAIITFSTLVQ--VVICTVWLVVAPPTPRQYMPRE--SAIIILLCDEGSTIAFSLVLGYIGVLACMCFLLAFLARKLPDNFNEARLIAFSMLIFCAVWVAFVPAYISSPGKY
611619  680 KWLGPKQQRTIIFSCTLVQ--VVICTAWLIDAPPAPFRNTEYE--HSKIILECSVGSSVGFWCVLGYIGLQACLSFILAFLARKLPGNFNEAKFITFSMLIFCAVWLAFIPAYVSSPGNY
611624  731 KLFGPSRQRTLILCTTAPQ--VCLCAGWLLAAPSFPVRNPAYQALTGKIVLECKEPWPPGFYLVLGYIGLLAFLCLLLAFVGRKLPNTFNEAKLITFSLLIFWAVWISFIPAYVSSPGKF
611613  717 KWFGPAQQRLCVVAFTFVQ--VLICILWLTISPPYPFENMDYY--NDRIILECALGSAVGFWAVLGYIGLLAMFCFILAFLARKLPNNFNEAKFITFSMLIFCAVWVTFIPAYVSSPGKF
735220  676 NFNLCLHCRCSFVL----Q--AVACTVWLILLPPHAIKNTSAQ--NIKIILECDEGSIVFICCVFAYDILLALIAFIFAFMARKLEDHFSEGKCMTFGMLVFFIVWISFVPAYLSTRGKF
179742  658 R--KIYQPYAIITICVALQ--TATCICWLVLLSPYAHIIK--Q--PTTLLQYCHEGSYVAFGVMLGYIAILAFVCFICAFKGRKLPEQYNEAKFITFSMLLYLISWLLFVPIYVTTSGVY

TM7 clip
594197  810 SVAVEIFAILSSSFGLLICIFIPKCYIILLKPERNTKKNLMGKEGPTH-----------------------------
744222  794 SVAVEIFAILSSGFGLLICIFIPKCYIILLKPERNTKRNLMGKEGPTH-----------------------------
594233  809 TVAVEIFAILASSYGMLFCIFLPKCYVILLKPENNTKKKLMGKV-MPRAL---------------------------
Ca12    810 TVAVEIFAILASSFGLLFCIFAPKCYILILKPEKNTKKHMMGRN-HQN-----------------------------
Ca02.1  825 TVAVEIFAILASSFGLILCIFAPKCFIILFKPEKNSKKHLMNKK---------------------------------
581784  825 TVAVEIFAILASSFGLILCIFAPKCFIILFKPEKNSKKHLMNKK---------------------------------
584633  826 TVAVEIFAILASSFGLILCIFAPKCFIILFKPEKNSKKHLMNKK---------------------------------
Ca09    822 ADAVEVFAILTSSFGLLVALFGPKCYIILFRPERNTKRAIMAR----------------------------------
589261  740 ADAVEVFAILASSFGLLAALFGPKCYIVLLRPEKNTKNAIMGRVK--------------------------------
716738  753 ADAVEVFAILASSFGLLVALFGPKCYIILLRPERNTKKAIMDRD-TRK-----------------------------
Ca15.1  833 SVAVEIFAILASSYGLLFCIFAPKCFIILLRPEKNTKKHLMMR----------------------------------
571614  811 VVAVEIFAILASSYGLLFCIFAPKCFIILLRPEKNTKKHLMSRN-KA------------------------------
744432  799 TVAVEIFAILASSFGLLLCIFFPKCYIIIFRPQKNCRKSLLGNP-QTRTL---------------------------
Ca13    834 STLTEIFAILASSYGLLGCIFAPKCYIILMKSEKNTRKHLMSKS-ERF-----------------------------
611619  796 SDAVESFAILASSFGLLFCMFAPKCYIILLKPERNTKQHLMGKE-NKSHAVS--------------------------
611624  849 TVAVEVFAILASSFGLLLCIFLPKCFIILLRPERNVKKGMTGKCLR-------------------------------
611613  833 LVAVEIFAILASSYGLLFCIFLPKCFIILFRPELNTKKHIIGKT---------------------------------
735220  788 MVAVQIFAILASSFGLLTCIFLPKCYILLIKPERNKEDMMRPRT-KPRDGTSTGTSASLATAATEVGTAVASSAIDD
179742  770 LPAVEMVVILISNYGILSCHFFPKCYIIFFKKEQNTRSAFRKNL-YEYSSKHMESSGSSGSN---------------
```
